# ECMpy, a Simplified Workflow for Constructing Enzymatic Constrained Metabolic Network Model

**DOI:** 10.3390/biom12010065

**Published:** 2022-01-02

**Authors:** Zhitao Mao, Xin Zhao, Xue Yang, Peiji Zhang, Jiawei Du, Qianqian Yuan, Hongwu Ma

**Affiliations:** Biodesign Center, Key Laboratory of Systems Microbial Biotechnology, Tianjin Institute of Industrial Biotechnology, Chinese Academy of Sciences, Tianjin 300308, China; mao_zt@tib.cas.cn (Z.M.); zhaox@tib.cas.cn (X.Z.); yang_x@tib.cas.cn (X.Y.); zhangpj@tib.cas.cn (P.Z.); dujw@tib.cas.cn (J.D.); yuan_qq@tib.cas.cn (Q.Y.)

**Keywords:** enzyme-constrained model, *Escherichia coli*, enzyme kinetics, protein subunit, overflow metabolism

## Abstract

Genome-scale metabolic models (GEMs) have been widely used for the phenotypic prediction of microorganisms. However, the lack of other constraints in the stoichiometric model often leads to a large metabolic solution space being inaccessible. Inspired by previous studies that take an allocation of macromolecule resources into account, we developed a simplified Python-based workflow for constructing enzymatic constrained metabolic network model (ECMpy) and constructed an enzyme-constrained model for *Escherichia coli* (ec*i*ML1515) by directly adding a total enzyme amount constraint in the latest version of GEM for *E. coli* (*i*ML1515), considering the protein subunit composition in the reaction, and automated calibration of enzyme kinetic parameters. Using ec*i*ML1515, we predicted the overflow metabolism of *E. coli* and revealed that redox balance was the key reason for the difference between *E. coli* and *Saccharomyces cerevisiae* in overflow metabolism. The growth rate predictions on 24 single-carbon sources were improved significantly when compared with other enzyme-constrained models of *E. coli.* Finally, we revealed the tradeoff between enzyme usage efficiency and biomass yield by exploring the metabolic behaviours under different substrate consumption rates. Enzyme-constrained models can improve simulation accuracy and thus can predict cellular phenotypes under various genetic perturbations more precisely, providing reliable guidance for metabolic engineering.

## 1. Introduction

Accurate prediction of metabolic phenotypes of an organism is a key goal of computational biology and has attracted more and more attention from researchers. For this purpose, many genome-scale metabolic models have been developed [1,2] and successfully applied for guiding metabolic engineering based on flux balance analysis (FBA) and other stoichiometry-based methods [3,4]. However, in many cases, a microorganism shows suboptimal metabolism [5,6] that is inconsistent with the optimal solution of FBA [7], implying that the metabolic capacity of an organism is also constrained by other factors. For example, overflow metabolism, involving incomplete oxidation of glucose to fermentation byproducts such as acetate and ethanol instead of using respiratory pathway even in the presence of oxygen [8] cannot be properly explained by models only considering reaction stoichiometries. Studies suggested that it is likely to be caused by the limited amount of protein molecules within the cell [9].

In recent years, researchers proposed several new methods that introduced new constraints such as cell volume limitation [10], protein resource allocation [11], enzyme activity and total protein mass [12,13], thermodynamics [14] into the model along with the stoichiometric constraints. FBA with molecular crowding (FBAwMC) [10] introduced both the crowding coefficient and cell volume constraint to limit the space occupied by enzymes. With the new constraints, the method successfully simulated the substrate hierarchy utilization in *E. coli* [10]. Adadi et al. further extended FBAwMC by introducing known enzyme kinetic parameters and proposed a new method called MOMENT (metabolic modeling with enzyme kinetics), which improved the prediction accuracy of intracellular fluxes and enzyme gene expression values [15]. In 2017, Sanchez et al. proposed a new construction workflow of the enzyme-constrained model (GECKO, genome-scale model to account for enzyme constraints, using kinetics and omics), which used an average enzyme saturation coefficient and determined the fraction of enzyme proteins from proteomics data [16]. They developed an enzyme-constrained model for *S. cerevisiae* using GECKO and made an accurate prediction of several metabolic phenotypes [16]. However, introducing the enzyme constraints into the original metabolic model using GECKO needs to be extensively revised by modifying every metabolic reaction with a pseudo-metabolite representing an enzyme and adding hundreds of exchange reactions for enzymes, which is complex and significant increases the model size. Bekiaris et al. further provided an automatic workflow (AutoPACMEN) for the construction of enzyme-constrained models inspired by MOMENT and GECKO, which only introduced one pseudo-reaction and pseudo-metabolite [17]. These two construction processes, GECKO and AutoPACMEN, have greatly facilitated the construction of enzyme-constrained models for each species, and successfully constructed for *S. cerevisiae* [16]*, Bacillus subtilis* [18], *Bacillus coagulans* [19], *E. coli* [20] and *Streptomyces coelicolor* [21], which have successfully applied to target prediction for enhancing the yield of products [18,20,21].

In the current study, we propose a simpler workflow called ECMpy by explicitly introducing an enzyme constraint without modifying existing metabolic reactions or adding new reactions. Using ECMpy workflow, we constructed a high-quality enzyme-constrained model for *E. coli* (ec*i*ML1515) based on its latest metabolic model *i*ML1515 [22], high coverage of enzyme kinetics data gathering from the literature [23], and automated enzyme kinetic parameter calibration process. We demonstrated that ec*i*ML1515 could simulate the sub-optimal metabolism such as overflow metabolism and the maximal growth rates under different carbon sources. The whole process for model construction and simulation is available at GitHub (https://github.com/tibbdc/ECMpy, accessed on 25 December 2021) for users to easily reproduce the results and use it as a reference to build enzyme-constrained models for other organisms.

## 2. Materials and Methods

### 2.1. The Workflow of ECMpy

**Figure 1:**
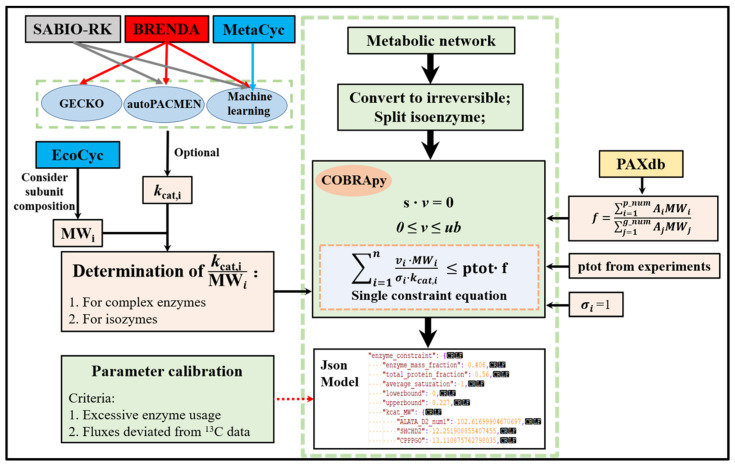
The ECMpy Workflow for Construction of Enzyme-constrained Models.

A metabolic network (like *i*ML1515 model in this study) was used as the initial model for the construction of enzyme-constrained models according to the workflow shown in Figure 1. Firstly, reversible reactions in the model were divided into two irreversible reactions because of different *k_cat_* values. The stoichiometric constraints (Equation (1)) and reversibility constraints (Equation (2)) used were the same as in flux balance analysis [24]. A new enzymatic constraint (Equation (3)) was introduced into the model, where *ptot* and *f* represent the total protein fraction in *E. coli* and the mass fraction of enzymes, respectively. The enzyme mass fraction *f* was calculated based on Equation (4) where *A_i_* and *A_j_* represented the abundances (mole ratio) of the *i*-th protein (*p_num* represented proteins expressed in the model) and *j*-th protein (*g_num* represented proteins expressed in the whole proteome). *MW_i_* and *k_cat_*_,*i*_ were molecular weight and turnover number of an enzyme catalyzing reaction *i*. For reactions catalyzed by multiple isoenzymes, a reaction can be split into multiple reactions. For reactions catalyzed by enzyme complex, using the minimum value of protein in complex ($\frac{k_{cat,i}}{MW_{i}}=min\left(\frac{k_{cat,ij}}{MW_{ij}},j\in m\right)$, *m* is the number of proteins in complex). $\sigma_{i}$ was the saturation coefficient of *i*-th enzyme.

(1) S·v=0 

(2) $$v_{lb}\;\leq \;v\;\leq \;v_{ub}$$

(3) $$\overset{n}{\underset{i=1}{\sum }}\frac{v_{i}\cdot MW_{i}}{\sigma_{i}\cdot k_{cat,i}}\leq ptot\cdot f$$

(4) $$f=\overset{p\_num}{\underset{i=1}{\sum }}A_{i}MW_{i}/\overset{g\_num}{\underset{j=1}{\sum }}A_{j}MW_{j}$$

### 2.2. Calibration of the Original k_cat_ Values

Generally, enzyme-constrained models need model validation (e.g., adjust the original *k_cat_* values to some extent to improve the agreement of model predictions with experimental data), similar to the way in GECKO and AutoPACMEN [17]. We proposed two principles (enzyme usage and ^13^C flux consistency) to adjust the original *k_cat_* values, as follows: First, a reaction with an enzyme usage exceeding 1% of the total enzyme content requires parameter correction; Second, a reaction with the *k_cat_* multiplied by 10% of the total enzyme amount ($v_{i}=\frac{10\%\times E_{total\;}\times \sigma_{i}\times k_{cat,i}}{MW_{i}}$) is less than the flux determined by ^13^C experiment needs to be corrected. All the *k_cat_* data used for correction comes from BRaunschweig ENzyme DAta base (BRENDA) and System for the Analysis of Biochemical Pathways - Reaction Kinetics databases (SABIO-RK) (using the maximum *k_cat_* value).

### 2.3. Simulation

We stored enzyme constraint information and metabolic network into JavaScript Object Notation (JSON) format, as the Systems Biology Markup Language (SBML) format cannot save enzyme constraints due to COBRApy [25] limitations. Then, we directly read the JSON file to obtain the enzyme-constrained model using the ‘get_enzyme_constraint_model’ function written by us. This transformed enzyme-constrained model is consistent with classical constraint-based models in format, which means that functions in COBRApy can be used directly on this model.

To evaluate ec*i*ML1515′s ability to predict growth rates, we compared the predicted results of *i*ML1515 and ec*i*ML1515 with experimental results performed by Adadi et al. [15], respectively. Specially, we set the upper bound of substrate uptake rate to 10 mmol/gDW/h and measured *E. coli*’s growth rates on 24 single carbon sources (e.g., acetate, fructose, fumarate and et al.). For comparison of each method on 24 single carbon sources, the model and experimental results were used to calculate the estimation error of the growth rate (Equation (5)) [26] and normalized flux error (Equation (6)) [27].

(5) $$\mathrm{estimation}\;\mathrm{error}=\frac{\left|v_{growth,sim}-v_{growth,exp}\right|}{v_{growth,exp}}\;$$

(6) $$\mathrm{normalized}\;\mathrm{flux}\;\mathrm{error}=\frac{\sqrt{\sum_{i}^{n}{\left(v_{growth,sim_{i}}-v_{growth,exp_{i}}\right)}^{2}}}{\sum_{i}^{n}{\left(v_{growth,exp_{i}}\right)}^{2}}\;$$

In addition to the maximal growth rates under different carbon sources, we also explored the overflow metabolic behaviours of *E. coli.* Especially, the growth rate is fixed (from 0.1 h^−1^ to 0.65 h^−1^) and glucose is supplied infinitely. Besides, we calculated the reaction enzyme cost (Equation (7)), energy synthesis enzyme cost (Equation (8)) and oxidative phosphorylation ratio (Equation (9)) to explore the adjustment strategy of *E. coli*’s overflow metabolic pathway.

(7) $${\mathrm{reaction}\;\mathrm{enzyme}\;\cos t}_{i}=\frac{v_{i}\cdot MW_{i}}{\sigma_{i}\cdot k_{cat,i}}$$

(8) $${\mathrm{energy}\;\mathrm{synthesis}\;\mathrm{enzyme}\;\cos t}_{i}=\overset{n}{\underset{i=1}{\sum }}{\mathrm{reaction}\;\mathrm{enzyme}\;\cos t}_{i}/v_{net\_generated\_ATP}\;$$

(9) $$\mathrm{oxidative}\;\mathrm{phosphorylation}\;\mathrm{ratio}=\frac{v_{O_{2}}}{v_{glucose}}\;$$

To obtain the trade-off between yield ($\frac{v_{biomass}}{v_{glucose}*MW_{glucose}}$) and enzyme usage efficiency ($\frac{v_{biomass}}{E_{min}}$), we developed a new method (Equations (10)–(14)) to calculate the minimum enzyme amount ($E_{min}$) inspired by pFBA (parsimonious FBA) [28]. When simulation, we set the concentration of glucose from 1 mmol/gDW/h to 10 mmol/gDW/h).

(10) $$obj:minimize\;\overset{n}{\underset{i=1}{\sum }}\frac{v_{i}\cdot MW_{i}}{\sigma_{i}\cdot k_{cat,i}}\;$$

(11) S·v=0

(12) $$v_{lb}\;\leq \;v\;\leq \;v_{ub}$$

(13) $$\overset{n}{\underset{i=1}{\sum }}\frac{v_{i}\cdot MW_{i}}{\sigma_{i}\cdot k_{cat,i}}\leq ptot\cdot f\;$$

(14) $$v_{biomass}=max\left(\mathrm{growth}\;\mathrm{rate}\right)$$

## 3. Results

### 3.1. Construction of the Enzyme-Constrained Model of iML1515 by ECMpy

The *i*ML1515 model was used as the initial model for the construction of the enzyme-constrained model. During the process, we observed that some errors in *i*ML1515 (e.g., GPR relationships, reaction direction and EC number, et al.) and corrected them based on information from the Encyclopedia of *Escherichia coli* genes and metabolism database (EcoCyc) [29] (see Table S1 for details). Then, we divided reversible reactions in *i*ML1515 into two irreversible reactions and split reactions catalyzed by multiple isoenzymes into different reactions (append num in reaction ID, e.g., ALATA_D2_num1). We found that the subunit composition of different proteins in *E. coli* differed significantly (Figure S1), so we took the subunit composition of proteins into account when calculating protein molecular weights. The molecular weights, subunit composition of enzymes in *i*ML1515 were obtained from EcoCyc. GECKO and sMOMENT (AutoPACMEN for *E. coli*) used the in vitro *k_cat_* which was obtained in labour-intensive, low-throughput in vitro assays and resulted in only a small fraction of cellular enzymes having a measured *k_cat_* even in model organisms [30]. That is why we used the *k_cat_* values derived from machine learning methods performed by Heckmann et al. [23]. In the model, *k_cat_* values were assigned to 2432 enzymatic reactions, and the coverage exceeds 60% (including isozyme split reactions and reversible split reactions, exclude exchange reactions), which is larger than the GECKO and sMOMENT (the number of reactions that matched EC number and substrate at the same time was only about 387). The protein fraction *ptot* was set at 0.56 g gDW^−1^ based on the experimentally measured macromolecular composition of *E. coli* cells [31,32]. The *E. coli* protein abundance values were obtained from the Protein abundance database (PAXdb) (https://pax-db.org/, accessed on 25 December 2021) [33] and the ‘whole organism (integrated)’ dataset with the highest coverage and credibility was selected. According to Equation (4), *f* was calculated to be 0.406 g enzyme/g protein.

**Figure 2:**
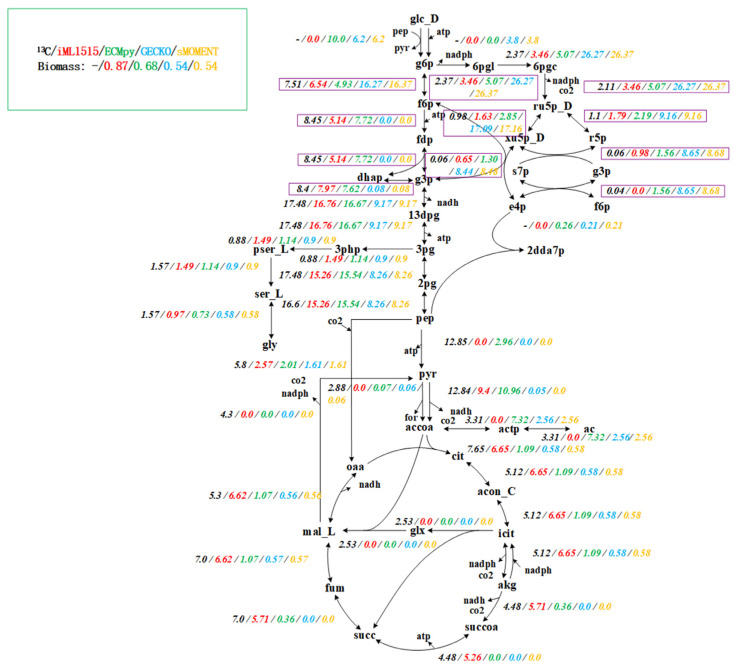
Flux Comparison of *i*ML1515, ECMpy, GECKO and sMOMENT. From left to right: ^13^C experimental data (black), prediction results of *i*ML1515 model (red), prediction results of ec*i*ML1515 constructed by ECMpy (green), prediction results of ec*i*ML1515 constructed by GECKO (blue), and prediction results of ec*i*ML1515 constructed by sMOMENT (yelllow).

However, the flux of growth rate predicted by this initial model is low and the conversion of phosphoenolpyruvate to the TCA pathway is abnormal (Figure S2). We first calibrated the reaction according to the enzyme usage, and changed 14 reactions (See Table S2 for details). The flux of growth rate predicted by the calibrated model increased to 0.5594 h^−1^, but the conversion of phosphoenolpyruvate to the TCA pathway was still abnormal (Figure S2). Subsequently, we compared with the ^13^C experimental data [34] and found that the *k_cat_* value of two reactions (PDH: pyruvate to acetyl-CoA and AKGDH: 2-oxoglutarate to succinyl-CoA) is low, which is mainly caused by the subunit composition of these two reactions is complicated and the protein molecular weight is very large. After calibration using ^13^C data (changed two reactions, Table S2), the growth rate increased to 0.6802 h^−1^, and the consistency with the pathway obtained by ^13^C data reached 92.1% (Figure S3). Different from other methods for constructing enzyme-constrained models, our method considers the composition of protein subunits and realizes enzyme constraint by simply adding the total enzyme amount equation (Table 1). Therefore, the enzyme-constrained model we constructed does not change the stoichiometric matrix format (because the isoenzyme reaction and reversible reaction were split, the number of reactions increased), and the solution and subsequent operations of the entire model are consistent with the classical constraint-based model. We used AutoPACMEN to build the GECKO and sMOMENT model of *i*ML1515, and compared them with ECMpy. We found that when considering the subunit composition of protein, the growth rate predicted by GECKO and sMOMENT model is lower, and the flux distribution of the pathway is abnormal from the ^13^C data, especially the EMP pathway (Figure 2, purple boxes).

### 3.2. Overflow Metabolism of E. coli

**Figure 3:**
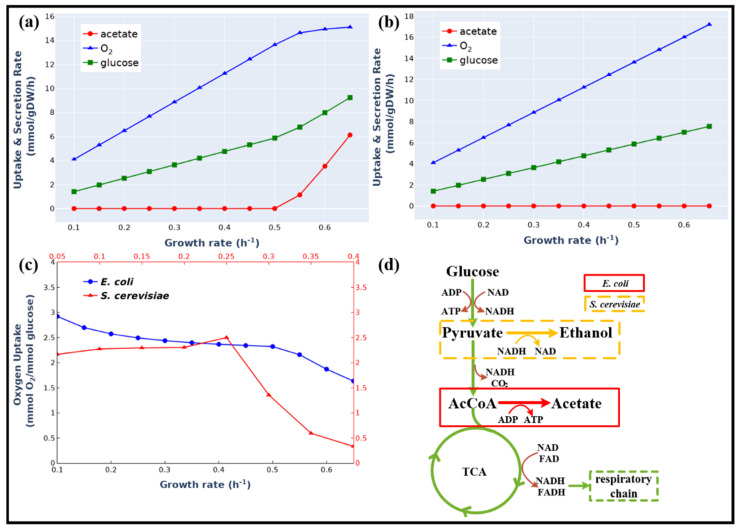
Comparison of Simulation Results of the Enzyme-constrained Model ec*i*ML1515 and the Stoichiometric Model *i*ML1515. Simulation of overflow metabolism at different growth rates using ec*i*ML1515 (**a**) and *i*ML1515 (**b**). (**c**) Simulated different overflow metabolism of *E. coli* and *S. cerevisiae*. (**d**) The different overflow metabolic pathways of *E. coli* and *S. cerevisiae*.

Overflow metabolism describes a phenomenon in which cells produce fermentation products even in the presence of oxygen that led to the waste of carbon sources [9]. Enzyme-constrained metabolic models have been used to simulate the overflow metabolism in *S. cerevisiae* [16,35]. To test our model, we applied it to simulate the overflow metabolism reported by literature [36], in which *E. coli* secreted acetate at high growth rates (above 0.5 h^−1^). As shown in Figure 3a,b, the ec*i*ML1515 model (the kinetic parameters for each reaction see Table S3) could precisely simulate the switch point where acetate production started. The simulation results indicated that at high growth rates, the acetate producing fermentation pathway was activated due to its low enzyme cost in comparison with the energetically-efficient oxidative respiratory pathway (0.62 g vs. 2.38 g enzyme for 1 mol ATP/h, Table S4).

The model also predicted a notable difference in the overflow metabolism between *E. coli* and *S. cerevisiae* (Figure 3c). In *S. cerevisiae*, the oxygen-consuming high-yield respiratory pathway was decreased to a very low value [37], whereas in *E. coli* the respiratory pathway was maintained at a high level (Figure 3a) even though the acetate production pathway was activated. A logical explanation for this is that the fermentation products of these two organisms are different. In *S. cerevisiae*, ethanol was produced and NADH was balanced in the fermentation pathway. However, in *E. coli*, acetate was produced and the excess NADH produced in the fermentation pathway needs to be balanced through the oxidative respiratory pathway (Figure 3d). This result was in agreement with the finding of a previous study [38].

### 3.3. Maximum Growth Rate of E. coli on Different Carbon Sources

**Figure 4:**
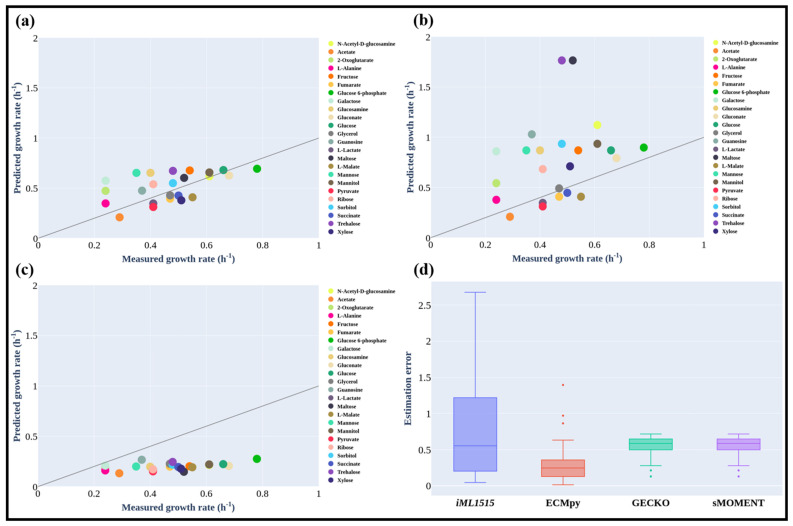
Predicted *E. coli* Growth Rates on Different Carbon Sources Using ECMpy (**a**), *i*ML1515 (**b**), GECKO and sMOMENT (**c**). (**d**) Distribution of prediction errors of internal fluxes from different models (GECKO and sMOMENT with consideration of protein subunits).

We simulated the maximum growth rates of *E. coli* on 24 different carbon sources and observed that certain other fermentation byproducts (e.g., pyruvate and fumarate) in addition to acetate could also be produced at the maximal growth rates. The predicted results were in good agreement with previously reported experimental results [15] as shown in Figure 4a (the normalized flux error is 0.062) and Table S5. On the other hand, the calculated growth rates using *i*ML1515 (the substrate uptake rates were set as the same as those for ec*i*ML1515) were significantly higher than the measured values (the standard flux error is 0.205, Figure 4b). The prediction results for most of the substrates (e.g., *N*-Acetyl-d-glucosamine and glucose) from ec*i*ML1515 were closer to (estimation error is 0.01 and 0.03) experimental values than those from the *i*ML1515 model. In a stoichiometric model such as *i*ML1515, the substrate uptake rate needs to be preset to calculate the growth rate and there is a linear relationship between the growth rate and substrate consumption rate. Whereas in the enzyme-constrained model, the maximal growth rate is limited by enzyme resources and thus there is no need to preset a substrate consumption rate. This means that at the maximal growth rate, a considerable quantity of substrates was utilized through the fermentation pathways with the secretion of fermentation products. Therefore, the predicted growth rates from the enzyme-constrained model were significantly lower than those from *i*ML1515 but much closer to the experimental findings. One exception for acetate as the carbon source is that the predicted results were the same for both models as no acetate producing fermentation pathway was activated in this case. From the results shown in Figure 4a, we can also see that for most carbon sources the predicted growth rates were still higher than the experimentally measured rates. This may imply that there are other constraints along with enzyme constraints limiting cellular growth, such as the regulatory or thermodynamic constraints. New models integrating these new constraints in a proper formula can further improve the prediction accuracy [39]. For xylose and glycerol, the predicted rates were smaller than the experimental values, implying that the *k_cat_* values of enzymes in the uptake pathways of these two substrates may be underestimated. Besides, we found that ECMpy is better than GECKO and sMOMENT for the simulation of growth rate on 24 different carbon sources (all consider protein subunits, but ECMpy corrected for enzyme kinetic parameters), and the simulation results of all enzyme-constrained models are also better than non-enzyme-constrained models (Figure 4a–d). This may also mean more precise measurement of the enzyme kinetic parameters could improve model prediction.

### 3.4. Simulation of the Trade-Off between Enzyme Usage Efficiency and Biomass Yield

**Figure 5:**
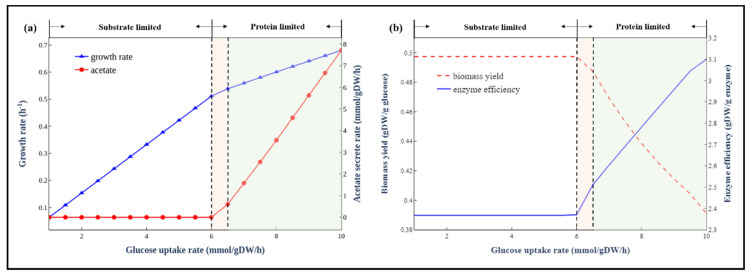
The metabolic behaviours of *E. coli* at different glucose uptake rates. (**a**) Simulated growth rates at different glucose uptake rates. (**b**) The trade-off between biomass yield and enzyme efficiency.

In addition to the maximal growth rates under different carbon sources, we also explored the metabolic behaviours of *E. coli* at different substrate (glucose as an example) uptake rates. As shown in Figure 5a,b, the metabolism processes can be divided into three stages: substrate-limited stage, overflow switching phase and overflow stage. At the first stage, the glucose uptake rate is low and has a linear relationship with growth rates. The biomass yield is almost constant (not the same as a small number of substrates are used for non-growth-related maintenance). At the second stage, the cell redistributes the intracellular fluxes toward pathways with high enzyme usage efficiency but low biomass yield, and acetate gradually becomes a byproduct of the newly activated pathways. In contrast, at the overflow stage, the organism has to activate the less energy efficient but higher enzyme usage efficiency fermentation pathway to produce the energy required for growth, leading to a sharp drop of biomass yield due to a big fraction of substrates used in the fermentation pathway. There was a clear trade-off between yield and enzyme usage efficiency (Figure 5b). These predicted metabolic behaviours were consistent with long-standing empirical models of microbial growth [40]. This trade-off phenomenon was also predicted by the *E. coli* ME-model [41], indicating that the enzyme-constrained model could accurately predict the same phenomenon as ME-model but without introducing thousands of new reactions involved in the transcription and translation process in the model.

## 4. Discussion

We constructed a genome-scale enzyme-constrained model ec*i*ML1515 for *E. coli* using the simplified Python-based ECMpy workflow. The new model was validated with various experimental data from literature including metabolic overflow data and the growth rates under different carbon sources. The prediction results were better than GECKO and sMOMENT, and those enzyme-constrained models were also better than the original *i*ML1515, indicating in these conditions enzyme availability rather than network stoichiometry is the key constraint. The enzyme-constrained model also showed a clear trade-off between biomass yield and enzyme usage efficiency. Switching from a high yield pathway to a high-rate pathway could be a general principle in metabolic regulation. This provides new insight into engineering organisms for the production of valuable biochemicals. In organisms using a high yield and high enzyme cost biosynthesis pathway, improving enzyme-specific activity could be more effective than enzyme overexpression.

Different from GECKO and sMOMENT, our method for enzyme constrained model construction just adds a constraint on the total amount of enzyme does not need to modify the reaction equations (e.g., introduce enzymes as reactants) and introduce over a thousand new enzyme exchange reactions (like GECKO). This greatly reduces the complexity in model construction and the model can be solved using COBRApy or other freely available python packages for constrained optimization. Besides, compared to the existing *E. coli* enzyme constraint model (using GECKO or sMOMENT), which set the number of each of the protein subunits to one, we defined the number of subunits for each protein in detail based on the EcoCyc. For example, pyruvate dehydrogenase consists of three subunits, AceE, AceF and Lpd, and its molecular weight is about 216.43 kD if the number of subunits is 1:1:1. However, the true number of subunits is 24:24:12 in EcoCyc, which means that the final molecular weight is 4586.16 kD. The large difference between the molecular weight of the same reaction would certainly cause a big difference in the flux results. The whole model construction and simulation processes were written in Jupyter Notebook files available from GitHub. This enables people from anywhere to reproduce the work and construct their enzyme constrained models for other organisms.

As we have shown that the quality of the enzyme constrained model depended largely on the quantity and accuracy of enzyme parameters. Even for *E. coli*, the enzyme kinetic data coverage is low in databases such as BRENDA and kinetic parameters from different researchers are often inconsistent. In this study, we make use of the predicted data from machine learning [23] to improve the data coverage. Besides, enzyme-constrained models need model validation to adjust the original *k_cat_* values to some extent to improve the agreement of model predictions with experimental data [17]. A system kinetic parameter correction method has been presented in the sMOMENT workflow [17], which helps identify such unreliable parameters and improve model prediction accuracy. However, this calibration workflow is time-consuming, going through protein pool calibration, manual *k_cat_* adjustment and automated *k_cat_* calibration, and there are some unreasonable places, such as the manual correction is simply expanded by 10 times or reduced by 10 times. In recently, GECKO 2.0 (https://doi.org/10.1101/2021.03.05.433259, accessed on 25 December 2021) provided an automatic procedure, in which the top enzymatic limitation on growth rate is identified and its correspondent *k_cat_* is then iteratively replaced by the highest one available in BRENDA for the given enzyme class until the growth rate fit is normal [42]. Currently, we propose a simpler calibration process that requires only two steps (enzyme usage and ^13^C flux consistency, see method) to update the *k_cat_* for a small number of reactions to achieve a better growth rate fit. This new calibration process will facilitate the construction of high-quality enzyme constraint models.

The ECMpy also has some areas that need improvement. First, ECMpy currently obtains protein subunit composition data manually, while a large amount of protein composition data are distributed in databases, such as BioCyc [43], Uniprot [44] and Complex Portal [45], so an automated tool to obtain them is urgently needed. Second, the rationale of the model needs to be further developed to consider more factors affecting the cost of the enzyme (e.g., thermodynamics and regulation).

## 5. Conclusions

We presented ECMpy, a simple open-source Python-based workflow, for constructing enzyme-constrained models based on enzyme kinetic parameters and proteomics data. Using this method, we constructed an enzyme constrained model ec*i*ML1515 for *E. coli*. By introducing the enzyme constraints, the model can predict the overflow metabolism and growth under different carbon sources more precisely than the stoichiometric model *i*ML1515. The construction method can be applied to construct enzyme constrained models for other organisms and the optimization framework can be extended to integrate other constraints such as thermodynamic feasibility to further reduce the solution space and subsequently improve model prediction accuracy.

## Figures and Tables

**Table 1:** Comparison of the Construction Methods of Enzyme-constrained Model.

Items	MOMENT	GECKO	AutoPACMEN	ECMpy
Subunit number	(not consider) ×	(consider) √	× (provide interface)	√
Proteomics	×	√	√	√
Saturation	1	0.46	1	1
Mass fraction of enzymes	0.56	0.448	0.095	0.227
Adding methods of enzyme constraints	add enzyme concentrations for each reaction and add the enzymes solvent capacity constraint	change stoichiometric matrix, and introduce a large number of pseudo-reaction and pseudo-metabolite	change stoichiometric matrix, and introduce one pseudo-reaction and pseudo-metabolite	only add a total enzyme constraint
Reaction reversibility	not split	split	part split	split
Isozyme	a reaction can be catalyzed by multiple enzymes	a reaction can be catalyzed by multiple enzymes	always assumes that the enzyme with the minimal cost is used	a reaction can be catalyzed by multiple enzymes
Filling method of missing *k_cat_*	the median turnover number across all reactions	match the *k*cat value to other substrates, organisms, or even introduce wild cards in the EC number.	Similar to GECKO	enzyme cost=0
Model calibration	×	√	√	√
Model type	Not provided	XML	XML	JSON

## Data Availability

The scripts and datasets generated during and/or analyzed during the current study can be found at: https://github.com/tibbdc/ECMpy (accessed on 25 December 2021).

## Supplementary Materials

The following are available online at https://www.mdpi.com/article/10.3390/biom12010065/s1, Figure S1: Number of subunits per complex (only consider complex with two or more subunits) in *E. coli*. Figure S2: Flux comparison of ec*i*ML1515_ori and ec*i*ML1515_adj_round1. Figure S3: Flux comparison of ec*i*ML1515_ori and ec*i*ML1515_adj_round2. Table S1: The modified genes. Table S2: The corrected kinetic parameters. Table S3: The kinetic parameters. Table S4: Enzyme cost of energy metabolism in *E. coli.* Table S5: The maximum growth rates.
