# Supplementary material for: ECMpy, a Simplified Workflow for Constructing Enzymatic Constrained Metabolic Network Model
Source: Biomolecules. 2022 Jan 2;12(1):65. doi: 10.3390/biom12010065 (PMC8773657; doi:10.3390/biom12010065)
Supplement: Supplementary file 1 [file biomolecules-12-00065-s001.zip › biomolecules-1511087-supp-update.pdf]

Supplementary Material

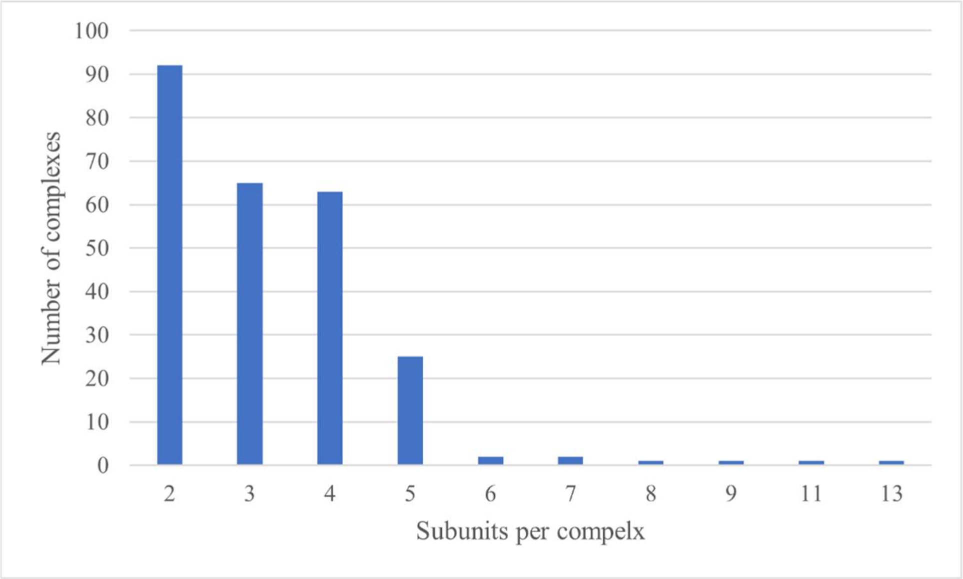

Figure S1 Number of subunits per complex (only consider complex with two or more subunits) in *E. coli*

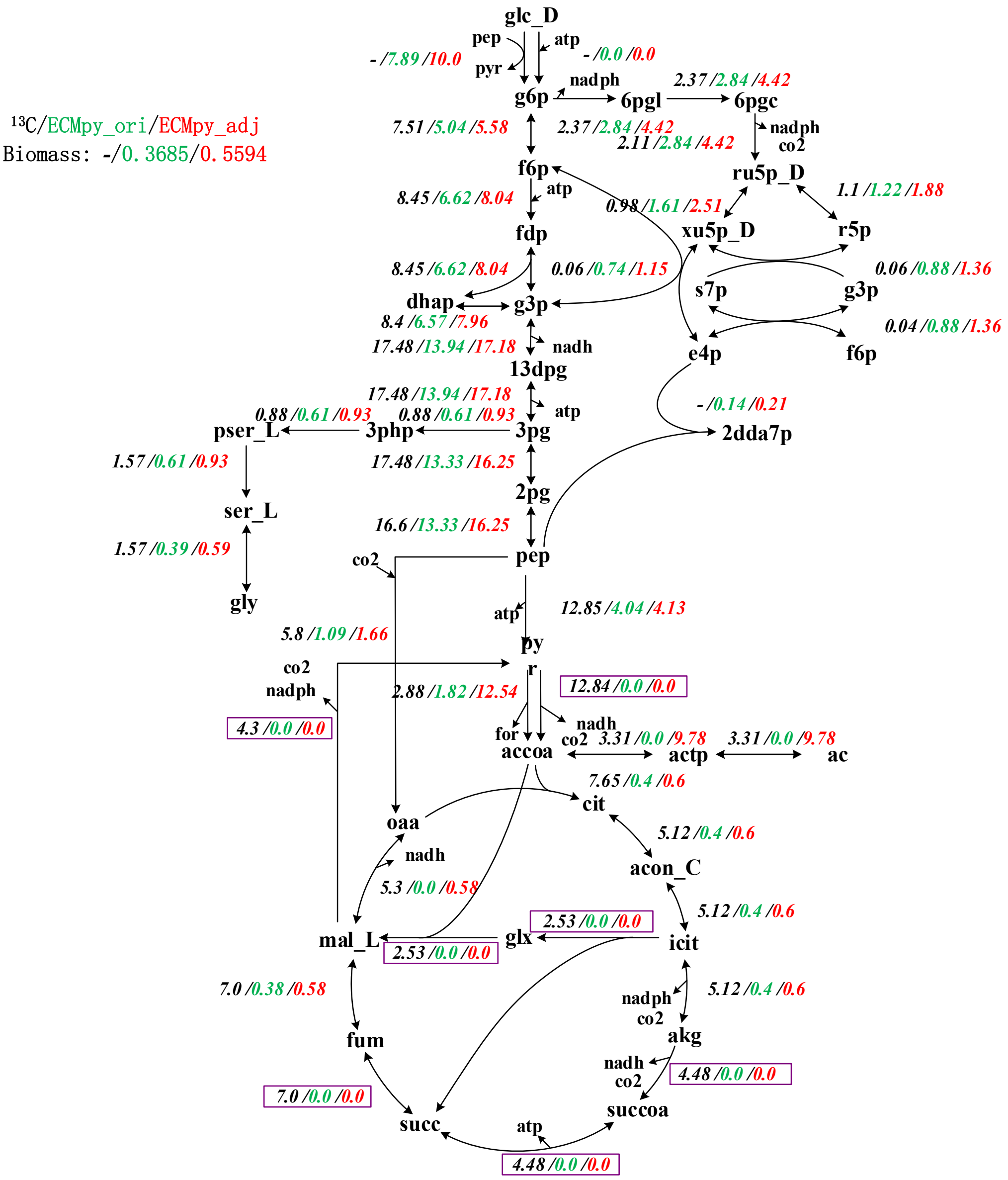

Figure S2. Flux comparison of ec*i*ML1515_ori and ec*i*ML1515_adj_round1. From left to right: ^{13}C experimental data, prediction results of original ECMpy model, and prediction results of calibrated ECMpy model (using enzyme amount).

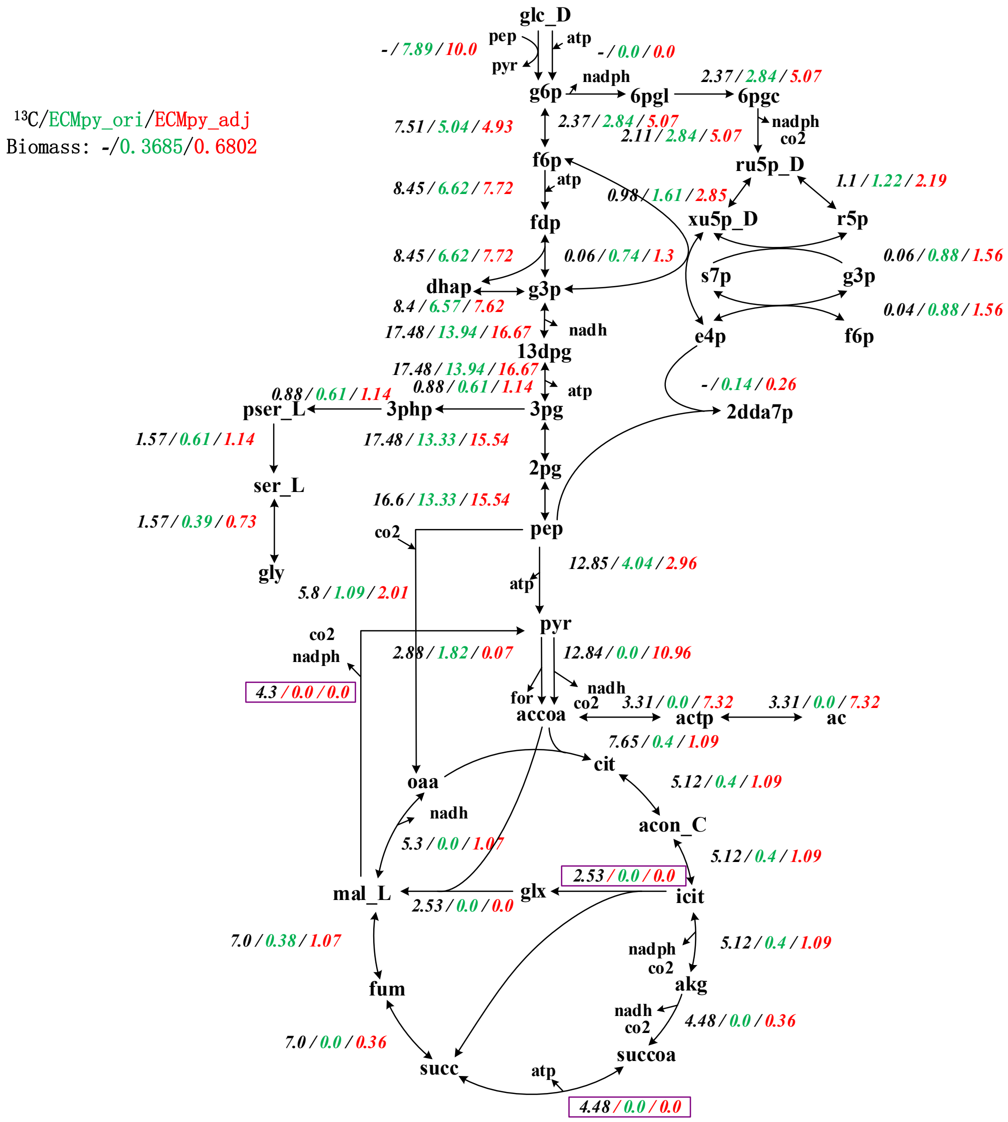

Figure S3. Flux comparison of ec*i*ML1515_ori and ec*i*ML1515_adj_round2. From left to right: ^{13}C experimental data, prediction results of original ECMpy model, and prediction results of calibrated ECMpy model (using enzyme amount and ^{13}C data).

Table S1 The modified genes

ID	Name	Equation	Gene_reaction_rule	Modified genes	Reason	Reference
FMNRx2	FMN reductase	fmn_c + h_c + nadph_c --> fmnh2_c + nadp_c	b0937 or b3844 or (b2764 and b2763)	b0937 or b3844 or b2764	CysIJ (b2763 and b2764) are the subunit of sulfite reductase, for this reaction only need b2764	[1]
ARGabcpp	L-arginine transport via ABC system (periplasm)	arg__L_p + atp_c + h2o_c --> adp_c + arg__L_c + h_c + pi_c	(b2310 and b2308 and b2306 and b2307) or (b0862 and b0860 and b0864 and b0863 and b0861)	(b2310 and b2308 and b2306 and b2307) or (b0862 and b0860 and b0864 and b0861) or (b0862 and b0864 and b0863 and b0861)	ArtI (b0863) is similar to the ArtJ (b0860) arginine binding periplasmic protein of *E. coli*	[2]
ECAP1pp	Enterobacterial common antigen polymerase (periplasm)	2.0 unagamuf_p --> eca2und_p + h_p + udcpdp_p	b3785 and b3793	b3785 and b3793 and b3792	WzxE (b3792) is a "flippase" responsible for movement of lipid III across the membrane bilayer	[3]
ECAP2pp	Enterobacterial common antigen polymerase (periplasm)	eca2und_p + unagamuf_p --> eca3und_p + h_p + udcpdp_p	b3785 and b3793	b3785 and b3793 and b3792	WzxE (b3792) is a "flippase" responsible for movement of lipid III across the membrane bilayer	[3]

FADRx2	FAD reductase	fad_c + h_c + nadph_c --> fadh2_c + nadp_c	b2764 and b2763	b2764	CysIJ (b2763 and b2764) are the subunit of sulfite reductase, not for this reaction	[1]
ECAP3pp	Enterobacterial common antigen polymerase (periplasm)	eca3und_p + unagamuf_p --> eca4und_p + h_p + udcpdp_p	b3785 and b3793	b3785 and b3793 and b3792	WzxE (b3792) is a "flippase" responsible for movement of lipid III across the membrane bilayer	[3]
BWCOGDS1	Tungsten bispterin guanine dinucleotide synthase (single GDP)	bwco_c + gtp_c + h_c --> bwco1gdp_c + ppi_c	(b3857 and b3856) or b3857	b3857	MobB (b3856) is only an adaptor protein, not a enzyme	[4]
BWCOGDS2	Tungsten bispterin guanine dinucleotide synthase	bwco1gdp_c + gtp_c + h_c --> bwcogdp_c + ppi_c	(b3857 and b3856) or b3857	b3857	MobB (b3856) is only an adaptor protein, not a enzyme	[4]
THZPSN3	Thiazole phosphate synthesis	atp_c + dhgly_c + dxyl5p_c + h_c + iscssh_c + nadph_c --> 4mpetz_c + amp_c + co2_c + 2.0 h2o_c + iscs_c + nadp_c + ppi_c	b3992 and b3990 and b0423 and b2530 and b4407	b3992 and b3990 and b0423 and b2530 and b4407 and b3991	ThiG (b3991) participates in the synthesis of the thiazole moiety of thiamine	[5]

BMOGDS1	Bis-molybdopterin guanine dinucleotide synthase (single GDP)	bmoco_c + gtp_c + h_c --> bmoco1gdp_c + ppi_c	b3857 or (b3857 and b3856)	b3857	MobB (b3856) is only an adaptor protein, not a enzyme	[5]
BMOGDS2	Bis-molybdopterin guanine dinucleotide synthase	bmoco1gdp_c + gtp_c + h_c --> bmocogdp_c + ppi_c	(b3857 and b3856) or b3857	b3857	MobB (b3856) is only an adaptor protein, not a enzyme	[4]
MOGDS	Molybdopterin guanine dinucleotide synthase	gtp_c + h_c + moco_c --> mocogdp_c + ppi_c	(b3857 and b3856) or b3857	b3857	MobB (b3856) is only an adaptor protein, not a enzyme	[4]
3NTD4pp	3'-nucleotidase (CMP) (periplasm)	3cmp_p + h2o_p --> cytd_p + pi_p	b0383 or b4213 or b0383	b0383 or b4213 or b2744	repeat	
GLUDy	Glutamate dehydrogenase (NADP)	glu__L_c + h2o_c + nadp_c <=> akg_c + h_c + nadph_c + nh4_c	b1761 or (b3213 and b3212)	b1761	b3213 and b3212 encode glutamate synthase, the corresponding reaction is GLUSy	

References

[1]. Siegel, L. M., D. C. 3. Rueger, M. J. Barber, R. J. Krueger, N. R. Orme-Johnson and W. H. Orme-Johnson (1982). "Escherichia coli sulfite reductase hemoprotein subunit. Prosthetic groups, catalytic parameters, and ligand complexes." J Biol Chem 257(11): 6343-6350.

[2]. Wissenbach, U., S. Six, J. Bongaerts, D. Ternes, S. Steinwachs and G. Unden (1995). "A third periplasmic transport system for L-arginine in Escherichia coli: molecular characterization of the artPIQMJ genes, arginine binding and transport." Mol Microbiol 17(4): 675-686.

[3]. Rick, P. D., K. Barr, K. Sankaran, J. Kajimura, J. S. Rush and C. J. Waechter (2003). "Evidence that the wzxE gene of Escherichia coli K-12 encodes a protein involved in the transbilayer movement of a trisaccharide-lipid intermediate in the assembly of enterobacterial. common antigen." J Biol Chem 278(19): 16534-16542
[4]. Palmer, T., C. L. Santini, C. Iobbi-Nivol, D. J. Eaves, D. H. Boxer and G. Giordano (1996). "Involvement of the narJ and mob gene products in distinct steps in the biosynthesis of the molybdoenzyme nitrate reductase in Escherichia coli." Mol Microbiol 20(4): 875-884.
[5]. Vander Horn, P. B., A. D. Backstrom, V. Stewart and T. P. Begley (1993). "Structural genes for thiamine biosynthetic enzymes (thiCEFGH) in Escherichia coli K-12." J Bacteriol 175(4): 982-992.

Table S2 The corrected kinetic parameters

Model	ID	Equation	k_{cat}(original)	MW	k_{cat}/MW (original)	k_{cat}(1/h) (corrected)	k_{cat}/MW (corrected)	growth rate
iML1515	-	-	-	-	-	-	-	0.8698
eciML1515	-	-	-	-	-	-	-	0.3685
eciML1515_round1	GAPD	g3p_c + nad_c + pi_c --> 13dpg_c + h_c + nadh_c	104400	142.13	734.54	3596400	25303.60	0.5594
	ENO	2pg_c --> h2o_c + pep_c	86400	91.31	946.23	828000	9068.02	
	FBA_num1	fdp_c --> dhap_c + g3p_c	37445.93	78.29	478.27	313200	4000.28	
	NADH16pp	4.0 h_c + nadh_c + q8_c --> 3.0 h_p + nad_c + q8h2_c	700176.04	540.41	1295.63	5400000	9992.34	
	RBFSa	4r5au_c + db4p_c --> dmlz_c + 2.0 h2o_c + pi_c	36	969.39	0.037	7200	7.43	
	KARA2	2ahbut_c + h_c + nadph_c --> 23dhmp_c + nadp_c	2700	216.28	12.48	281880	1303.33	
	ACCOAC	accoa_c + atp_c + hco3_c --> adp_c + h_c + malcoa_c + pi_c	28564.89	252.46	113.15	132480	524.77	

	METS_num2	5mthf_c + hcys__L_c --> h_c + met__L_c + thf_c	1116	84.67	13.18	3596400	42473.74	
	GND	6pgc_c + nadp_c --> co2_c + nadph_c + ru5p__D_c	79200	102.96	769.21	1170000	11363.35	
	GHMT2r	ser__L_c + thf_c --> gly_c + h2o_c + mlthf_c	11520	90.63	127.11	3596400	39680.82	
	GLNS	atp_c + glu__L_c + nh4_c --> adp_c + gln__L_c + h_c + pi_c	119723.28	622.85	192.22	3596400	5774.15	
	PPC	co2_c + h2o_c + pep_c --> h_c + oaa_c + pi_c	165600	396.25	417.92	3596400	9076.08	
	METAT	atp_c + h2o_c + met__L_c --> amet_c + pi_c + ppi_c	21.6	167.81	0.13	27000	160.90	
	PGI	g6p_c --> f6p_c	255600	123.06	2077.04	41112000	334082.29	
eciML1515_round2	AKGDH	akg_c + coa_c + nad_c --> co2_c + nadh_c + succoa_c	112697.45	2418.39	46.60	3596400	1487.10	0.6802
	PDH	coa_c + nad_c + pyr_c --> accoa_c + co2_c + nadh_c	270261.52	4586.61	58.92	3596400	784.11	

Table S3 The corrected kinetic parameters

ID	MW(kDa)	kcat(1/h)(original)	kcat/MW(original)	kcat(1/h)(corrected)	kcat/MW(corrected)
ALATA_D2_num1	90.6332	9300.506998	102.616999	9300.506998	102.616999
ALATA_D2_num2	145.9788	9300.506998	63.71135396	9300.506998	63.71135396
SHCHD2	99.9028	1224	12.25190886	1224	12.25190886
CPPPGO	68.6453	900	13.11087576	900	13.11087576
GTHOr	97.545	25901.61614	265.5350468	25901.61614	265.5350468
DHORD5	36.7745	38657.17404	1051.195096	38657.17404	1051.195096
GLYCTO2	137.2828	50485.46818	367.7479493	50485.46818	367.7479493
GLYCTO3	137.2828	57086.12156	415.8286513	57086.12156	415.8286513
GLYCTO4	137.2828	117077.842	852.8223641	117077.842	852.8223641
PFK_3	139.3682	208800	1498.189687	208800	1498.189687
TRPS2	143.4143	9309.205094	64.9112752	9309.205094	64.9112752
G3PD5_num1	148.4235	60326.17992	406.4462832	60326.17992	406.4462832
G3PD5_num2	113.5011	60326.17992	531.503042	60326.17992	531.503042
LEUTAi_num1	204.5619	54098.62261	264.4608923	54098.62261	264.4608923
LEUTAi_num2	87.0756	54098.62261	621.2833746	54098.62261	621.2833746
SHK3Dr	29.4136	46104.63948	1567.459933	46104.63948	1567.459933
SHK3Dr_reverse	62.4556	29504.42482	472.4063946	29504.42482	472.4063946
G5SD	178.5202	50400	282.3209922	50400	282.3209922
ALATA_L2_num1	145.9788	22488.04715	154.0500891	22488.04715	154.0500891
ALATA_L2_num2	90.6332	22488.04715	248.1215178	22488.04715	248.1215178
MAN6PI	42.85	39462.13397	920.9366154	39462.13397	920.9366154

APRAUR	80.6766	1152	14.27923338	1152	14.27923338
DB4PS	46.7069	1224	26.20597813	1224	26.20597813
RBFK	34.7343	324	9.327955364	324	9.327955364
ACP1_FMN_num1	30.3709	23467.23572	772.688189	23467.23572	772.688189
ACP1_FMN_num2	27.1218	23467.23572	865.2536233	23467.23572	865.2536233
ACP1_FMN_num3	30.1962	23467.23572	777.1585736	23467.23572	777.1585736
RBFSb	70.3347	108	1.535515187	108	1.535515187
DMATT	32.1596	432	13.4330029	432	13.4330029
GRTT	32.1596	432	13.4330029	432	13.4330029
UPP3S	27.7979	3600	129.5061857	3600	129.5061857
UPPDC1	39.2481	2916	74.29659015	2916	74.29659015
TMDPP	94.4142	30479.00062	322.8222092	30479.00062	322.8222092
HCO3E_num1	100.3871	32760	326.3367504	32760	326.3367504
HCO3E_num2	47.5285	32760	689.2706481	32760	689.2706481
IMPC	57.3292	17248.07481	300.8602041	17248.07481	300.8602041
IMPD	208.0898	6480	31.14040188	6480	31.14040188
ECOAH4_num1	77.0722	41618.87183	539.9984927	41618.87183	539.9984927
ECOAH4_num2	159.1878	41618.87183	261.4451097	41618.87183	261.4451097
GLUTRS	53.8157	324	6.020547907	324	6.020547907
TMPPP	23.0153	72	3.128353747	72	3.128353747
URIK1	24.3531	19423.5383	797.5797043	19423.5383	797.5797043
URIK2	24.3531	9433.623334	387.3684801	9433.623334	387.3684801

CYTDK1	24.3531	9981.4541	409.8637997	9981.4541	409.8637997
CYTDK2	24.3531	9559.089353	392.5204328	9559.089353	392.5204328
XPPT	67.8824	10439.90187	153.7939417	10439.90187	153.7939417
HXPRT_num1	67.8824	17665.84585	260.2419161	17665.84585	260.2419161
HXPRT_num2	80.461	17665.84585	219.5578709	17665.84585	219.5578709
NDPK5_num1	23.586	9864.913493	418.2529252	9864.913493	418.2529252
NDPK5_num2	61.8538	9864.913493	159.48759	9864.913493	159.48759
DHORTS	77.6548	37265.83384	479.8909254	37265.83384	479.8909254
OMPDC	52.7005	86400	1639.453136	86400	1639.453136
CS	288.0899	104400	362.3868799	104400	362.3868799
ICDHyr	91.5134	39600	432.7235137	39600	432.7235137
ACALD_num1	33.4425	347838.2822	10401.08491	347838.2822	10401.08491
ACALD_num2	3845.0896	347838.2822	90.46298484	347838.2822	90.46298484
ACS	72.0936	32028.81133	444.2670546	32028.81133	444.2670546
PPA_num1	116.2719	303342.6722	2608.907846	303342.6722	2608.907846
PPA_num2	26.9004	303342.6722	11276.51158	303342.6722	11276.51158
PPA_num3	118.2214	303342.6722	2565.886313	303342.6722	2565.886313
PPCK	59.6435	97061.54587	1627.361672	97061.54587	1627.361672
ME1	252.7893	60940.10038	241.0707272	60940.10038	241.0707272
ALAR_num1	77.6892	113967.7232	1466.969968	113967.7232	1466.969968
ALAR_num2	78.3059	113967.7232	1455.416811	113967.7232	1455.416811
ALATA_L_num1	91.0347	63159.7104	693.7981934	63159.7104	693.7981934
ALATA_L_num2	92.432	63159.7104	683.3070482	63159.7104	683.3070482

	4				
XYLK_num1	122.1788	24801.49779	202.9934636	24801.49779	202.9934636
XYLK_num2	105.2365	24801.49779	235.6739135	24801.49779	235.6739135
RBK	64.581	59351.15383	919.018811	59351.15383	919.018811
GLYK	224.9231	45670.44197	203.0491398	45670.44197	203.0491398
PPM	44.37	158919.1397	3581.679958	158919.1397	3581.679958
ASPTA_num1	87.1467	13921.64902	159.7495834	13921.64902	159.7495834
ASPTA_num2	87.0756	13921.64902	159.880024	13921.64902	159.880024
FBP_num1	68.6469	274394.2568	3997.183511	274394.2568	3997.183511
FBP_num2	147.3357	274394.2568	1862.374542	274394.2568	1862.374542
FBP_num3	71.7046	274394.2568	3826.731573	274394.2568	3826.731573
FBP_num4	29.7211	274394.2568	9232.304887	274394.2568	9232.304887
GLGC	194.7906	18829.61562	96.66593572	18829.61562	96.66593572
PYK_num1	205.4286	427444.7782	2080.746197	427444.7782	2080.746197
PYK_num2	202.9177	427444.7782	2106.493313	427444.7782	2106.493313
A5PISO_num1	140.7843	55549.16712	394.5693314	55549.16712	394.5693314
A5PISO_num2	136.1257	55549.16712	408.0725911	55549.16712	408.0725911
IPPMIb	72.3693	23255.09457	321.3392222	23255.09457	321.3392222
ACHBS_num1	143.0927	67064.87156	468.6812924	67064.87156	468.6812924
ACHBS_num2	161.9222	67064.87156	414.1795971	67064.87156	414.1795971
ACLS_num1	161.9222	241177.3205	1489.464202	241177.3205	1489.464202
ACLS_num2	143.0927	241177.3205	1685.462085	241177.3205	1685.462085
PSCVT	46.0958	75600	1640.062652	75600	1640.062652

ANS	228.7288	17526.9833	76.62779371	17526.9833	76.62779371
ANPRT	113.7399	18000	158.25581	18000	158.25581
CHORM_num1	86.2227	123098.1309	1427.676596	123098.1309	1427.676596
CHORM_num2	84.085	123098.1309	1463.972538	123098.1309	1463.972538
CHORS	156.5498	151200	965.8268487	151200	965.8268487
IGPS	49.4923	18000	363.6929381	18000	363.6929381
ECOAH6_num1	77.0722	36593.98049	474.8012966	36593.98049	474.8012966
ECOAH6_num2	159.1878	36593.98049	229.8793029	36593.98049	229.8793029
ENO	91.3099	86400	946.2281746	828000	9068.020007
FBA_num1	78.2945	37445.93179	478.2702718	313200	4000.28099
FBA_num2	381.0919	37445.93179	98.25958461	37445.93179	98.25958461
MGSA	101.5115	10091.64245	99.41378514	10091.64245	99.41378514
PPS	174.8702	370888.9427	2120.938517	370888.9427	2120.938517
PGI	123.0595	255600	2077.044032	41112000	334082.2935
PGK	41.1182	81612.68645	1984.831205	81612.68645	1984.831205
EDA	66.8521	44337.53138	663.2182293	44337.53138	663.2182293
PGL	36.3076	662400	18244.11418	662400	18244.11418
RPE	24.5542	198533.1876	8085.508288	198533.1876	8085.508288
TALA_num1	70.4385	49937.71597	708.9548467	49937.71597	708.9548467
TALA_num2	35.6588	49937.71597	1400.431758	49937.71597	1400.431758
TKT1_num1	144.4235	182904.1236	1266.442951	182904.1236	1266.442951
TKT1_num2	73.0427	182904.1236	2504.07123	182904.1236	2504.07123

G6PDA	178.6452	151224.2552	846.5061206	151224.2552	846.5061206
ALCD2x_num1	3845.0896	65388.1289	17.00561904	65388.1289	17.00561904
ALCD2x_num2	141.5183	65388.1289	462.0471621	65388.1289	462.0471621
ALDD2y_num1	29.4102	22093.1682	751.2076831	22093.1682	751.2076831
ALDD2y_num2	84.194	22093.1682	262.4078699	22093.1682	262.4078699
ALDD2y_num3	225.2254	22093.1682	98.09359069	22093.1682	98.09359069
ALDD2y_num4	31.488	22093.1682	701.6377096	22093.1682	701.6377096
ALDD2y_num5	36.5018	22093.1682	605.2624309	22093.1682	605.2624309
RMI	188.7978	34888.97517	184.7954541	34888.97517	184.7954541
FMNAT	34.7343	324	9.327955364	324	9.327955364
NADDP_num1	59.3779	10638.00544	179.157657	10638.00544	179.157657
NADDP_num2	73.6061	10638.00544	144.526139	10638.00544	144.526139
NMNAT_num1	189.3833	9306.715666	49.14221933	9306.715666	49.14221933
NMNAT_num2	24.528	9306.715666	379.4323086	9306.715666	379.4323086
HMPK1_num1	114.5344	15927.76481	139.065336	15927.76481	139.065336
HMPK1_num2	61.6948	15927.76481	258.1702966	15927.76481	258.1702966
NDPK6_num1	23.586	9855.575546	417.8570146	9855.575546	417.8570146
NDPK6_num2	61.8538	9855.575546	159.3366219	9855.575546	159.3366219
NDPK8_num1	23.586	15171.87038	643.2574568	15171.87038	643.2574568
NDPK8_num2	61.8538	15171.87038	245.2859869	15171.87038	245.2859869
PYNP2r	162.9545	44972.73011	275.983358	44972.73011	275.983358
DURIPP_num1	94.4142	31920.23239	338.0871987	31920.23239	338.0871987
DURIPP_num2	155.6995	31920.23239	205.0117848	31920.23239	205.0117848
PTRCTA	99.3227	36125.7943	363.7214282	36125.7943	363.7214282

CYSDS_num1	172.8495	13182.37802	76.26506305	13182.37802	76.26506305
CYSDS_num2	211.0938	13182.37802	62.44796396	13182.37802	62.44796396
TRPAS2	211.0938	23909.43164	113.2644902	23909.43164	113.2644902
NDP3_num1	17.4329	34101.47047	1956.155916	34101.47047	1956.155916
NDP3_num2	35.3527	34101.47047	964.6072427	34101.47047	964.6072427
CDPPH	35.3527	28855.36431	816.2138763	28855.36431	816.2138763
NDP7	35.3527	33721.60938	953.8623465	33721.60938	953.8623465
PMDPHT_num1	30.1962	9471.620437	313.6692841	9471.620437	313.6692841
PMDPHT_num2	27.1218	9471.620437	349.2253625	9471.620437	349.2253625
ILETA	204.5619	22229.68578	108.6697268	22229.68578	108.6697268
VALTA	204.5619	9473.113026	46.30927375	9473.113026	46.30927375
ORPT	47.134	11843.91592	251.2817906	11843.91592	251.2817906
RBFSa	969.3906	36	0.037136733	7200	7.427346624
DHAD2	131.0631	500400	3818.008272	500400	3818.008272
G1PP_num1	22.7318	9921.660322	436.4661101	9921.660322	436.4661101
G1PP_num2	23.0075	9921.660322	431.2359153	9921.660322	431.2359153
G1PP_num3	29.7211	9921.660322	333.8254749	9921.660322	333.8254749
PFL_num1	118.3894	66265.40938	559.7241761	66265.40938	559.7241761
PFL_num2	198.9187	66265.40938	333.128104	66265.40938	333.128104
PFL_num3	114.1398	66265.40938	580.5635666	66265.40938	580.5635666
PFL_num4	213.2029	66265.40938	310.8091371	66265.40938	310.8091371
FRD2	121.2166	39705.41401	327.5575623	39705.41401	327.5575623

FRD3	121.2166	40135.54568	331.1060175	40135.54568	331.1060175
POX	248.0455	11451.84521	46.1683248	11451.84521	46.1683248
PTAr_num1	463.0325	547821.7643	1183.117307	547821.7643	1183.117307
PTAr_num2	72.1335	547821.7643	7594.554046	547821.7643	7594.554046
ACKr_num1	42.4337	168823.2518	3978.518295	168823.2518	3978.518295
ACKr_num2	43.2904	168823.2518	3899.784982	168823.2518	3899.784982
ACKr_num3	43.3841	168823.2518	3891.362315	168823.2518	3891.362315
MOAT	47.2911	212400	4491.33135	212400	4491.33135
UDCPDPS	56.8883	504	8.859466709	504	8.859466709
USHD	26.8942	20475.11943	761.3210071	20475.11943	761.3210071
LPADSS	84.7649	187200	2208.461285	187200	2208.461285
TDSK	35.5893	26884.71438	755.4156554	26884.71438	755.4156554
NTPP1_num1	14.9271	29218.07367	1957.384466	29218.07367	1957.384466
NTPP1_num2	60.8246	29218.07367	480.3660635	29218.07367	480.3660635
NTPP2_num1	14.9271	10172.32877	681.4671817	10172.32877	681.4671817
NTPP2_num2	60.8246	10172.32877	167.2403726	10172.32877	167.2403726
NTPTP1	356.2976	28199.90188	79.14704417	28199.90188	79.14704417
NTPTP2	356.2976	58754.90664	164.9040202	58754.90664	164.9040202
IPMD_num1	79.034	17640	223.1950806	17640	223.1950806
IPMD_num2	40.3149	17640	437.5553455	17640	437.5553455
AIRC3	142.2426	11996.22974	84.33640654	11996.22974	84.33640654
ADSL2r	206.1712	54000	261.9182505	54000	261.9182505

PUNP5_num1	179.0077	18776.87584	104.8942355	18776.87584	104.8942355
PUNP5_num2	155.6995	18776.87584	120.5968924	18776.87584	120.5968924
PUNP6_num1	155.6995	19606.60291	125.9259208	19606.60291	125.9259208
PUNP6_num2	179.0077	19606.60291	109.5293829	19606.60291	109.5293829
PUNP7	179.0077	19138.3919	106.9137914	19138.3919	106.9137914
RNDR1_num1	274.1399	9882.90527	36.05059048	9882.90527	36.05059048
RNDR1_num2	270.3917	9882.90527	36.5503278	9882.90527	36.5503278
RNDR3_num1	270.3917	12566.47479	46.47507593	12566.47479	46.47507593
RNDR3_num2	274.1399	12566.47479	45.83964169	12566.47479	45.83964169
RNDR4_num1	270.3917	10859.48639	40.16205523	10859.48639	40.16205523
RNDR4_num2	274.1399	10859.48639	39.61293628	10859.48639	39.61293628
TMDS	60.9594	26640	437.0121753	26640	437.0121753
TMDK1	23.4566	9983.48333	425.6151075	9983.48333	425.6151075
PPCDC	521.2577	540	1.03595592	540	1.03595592
FTHFD	191.6083	11273.54495	58.83641234	11273.54495	58.83641234
EDD	64.639	48328.78694	747.6722558	48328.78694	747.6722558
DHQTi	54.9333	212400	3866.507201	212400	3866.507201
PUNP1	155.6995	17754.49068	114.0304926	17754.49068	114.0304926
PUNP2	155.6995	16381.18715	105.2102746	16381.18715	105.2102746
PUNP3_num1	179.0077	16041.29791	89.61233461	16041.29791	89.61233461
PUNP3_num2	155.6995	16041.29791	103.0272924	16041.29791	103.0272924
LYSAM	38.7496	26321.80499	679.2793988	26321.80499	679.2793988

DHAD1	131.0631	1512000	11536.42787	1512000	11536.42787
IPPMIa	72.3693	11963.94288	165.3179301	11963.94288	165.3179301
G3PD7	148.4235	97058.71559	653.9309179	97058.71559	653.9309179
NADH9_num1	40.34	10293.42646	255.1667441	10293.42646	255.1667441
NADH9_num2	47.3587	10293.42646	217.3502748	10293.42646	217.3502748
NADH9_num3	83.3822	10293.42646	123.4487272	10293.42646	123.4487272
NADH9_num4	40.7511	10293.42646	252.5926038	10293.42646	252.5926038
NADH10_num1	40.34	21675.04155	537.3089131	21675.04155	537.3089131
NADH10_num2	47.3587	21675.04155	457.6781363	21675.04155	457.6781363
NADH10_num3	40.7511	21675.04155	531.8885025	21675.04155	531.8885025
NADH10_num4	83.3822	21675.04155	259.9480651	21675.04155	259.9480651
MDH	64.6746	25920	400.775575	25920	400.775575
FUM_num1	120.2106	104334.9147	867.9343976	104334.9147	867.9343976
FUM_num2	120.5972	104334.9147	865.1520491	104334.9147	865.1520491
FUM_num3	201.9555	104334.9147	516.6232893	104334.9147	516.6232893
PYDXNK	61.6948	9300.850736	150.7558293	9300.850736	150.7558293
PYAM5PO	51.0903	9300.846143	182.0472016	9300.846143	182.0472016
OHPBAT	79.5666	3.6	0.045245115	3.6	0.045245115
GLUTRR	92.6136	9680.98199	104.5308895	9680.98199	104.5308895
GLCS1	52.8224	12943.08398	245.030214	12943.08398	245.030214
GLCP_num1	186.3451	26911.79951	144.4191423	26911.79951	144.4191423
GLCP_num2	181.0448	26911.79951	148.647183	26911.79951	148.647183
HEMEOS	32.248	10296.6826	319.2918305	10296.6826	319.2918305

	5				
ADMDC	30.3846	99039.6036	3259.532908	99039.6036	3259.532908
AGMT	67.1141	34948.09724	520.726602	34948.09724	520.726602
ARGDC	844.2524	10396.28729	12.31419335	10396.28729	12.31419335
ARGSS	199.5936	10080	50.50262133	10080	50.50262133
AGPR	35.9521	27140.71295	754.9131469	27140.71295	754.9131469
SPMS	64.6428	20729.78166	320.6819888	20729.78166	320.6819888
G1SAT	90.7322	324	3.570948351	324	3.570948351
KDOCT2	27.6144	15120	547.5404137	15120	547.5404137
OAADC	66.8521	17454.56417	261.0922344	17454.56417	261.0922344
MNNH	44.838	54841.49543	1223.103069	54841.49543	1223.103069
ALTRH	54.0931	69858.52128	1291.449765	69858.52128	1291.449765
GUI1	53.9872	86159.1663	1595.918408	86159.1663	1595.918408
PGAMT	47.5436	36035.4739	757.9458412	36035.4739	757.9458412
UAGDP	147.5702	57600	390.3227074	57600	390.3227074
UAGCVT	44.8176	11880	265.074435	11880	265.074435
GLUR	31.002	88419.50993	2852.058252	88419.50993	2852.058252
UAG2E	84.4892	57209.15714	677.1179884	57209.15714	677.1179884
MACPD	85.2266	17858.85608	209.5455653	17858.85608	209.5455653
DMQMT	53.1109	22755.67142	428.4557674	22755.67142	428.4557674
ICHORS_copy1	42.9316	50074.22462	1166.372197	50074.22462	1166.372197
HACD5_num1	159.1878	40209.19967	252.5897064	40209.19967	252.5897064
HACD5_num2	77.0722	40209.19967	521.7082121	40209.19967	521.7082121

HPPK2	18.0788	576	31.86052172	576	31.86052172
PTPATi	107.0198	1980	18.5012493	1980	18.5012493
HEX7	32.4998	31530.95853	970.1893098	31530.95853	970.1893098
DXPRIi	86.776	11880	136.9042131	11880	136.9042131
OPHBDC	333.6225	9504.978952	28.49022159	9504.978952	28.49022159
OHPHM	53.1109	24071.53907	453.2316166	24071.53907	453.2316166
GHMT2r	90.6332	11520	127.1057405	3596400	39680.82336
DHORD2	36.7745	72000	1957.878421	72000	1957.878421
ADK4	23.586	9420.738358	399.4207732	9420.738358	399.4207732
INSK	96.8976	38426.53993	396.5685418	38426.53993	396.5685418
ABTA_num1	91.5493	33853.97349	369.7895395	33853.97349	369.7895395
ABTA_num2	44.7291	33853.97349	756.8668605	33853.97349	756.8668605
SSALx	99.4357	21752.97002	218.7641865	21752.97002	218.7641865
ASPT	209.4245	20574.12224	98.24123843	20574.12224	98.24123843
SERD_L_num1	211.0938	78829.0727	373.4314921	78829.0727	373.4314921
SERD_L_num2	48.9066	78829.0727	1611.828929	78829.0727	1611.828929
SERD_L_num3	97.0433	78829.0727	812.3082449	78829.0727	812.3082449
SERD_L_num4	48.7529	78829.0727	1616.910434	78829.0727	1616.910434
SERD_L_num5	140.9296	78829.0727	559.3507163	78829.0727	559.3507163
THRD_num1	148.9562	74588.90951	500.7439067	74588.90951	500.7439067
THRD_num2	40.3513	74588.90951	1848.488388	74588.90951	1848.488388
KARA1	216.2761	15966.4598	73.82442999	15966.4598	73.82442999
KARA2	216.27	2700	12.48404239	281880	1303.334025

	61				
ACCOAC	252.4556	28564.88644	113.1481593	132480	524.7655429
NADK	195.3983	3960	20.2662971	3960	20.2662971
ICHORS_copy2	97.5291	24753.81242	253.8095032	24753.81242	253.8095032
SUCBZS	35.4766	25537.98661	719.8544001	25537.98661	719.8544001
DHBD	104.9986	14589.98073	138.9540502	14589.98073	138.9540502
ICHORT	32.5543	13917.92539	427.529555	13917.92539	427.529555
RBK_L1	122.1788	26478.88451	216.7224143	26478.88451	216.7224143
RBP4E_num1	25.2777	84320.75084	3335.776231	84320.75084	3335.776231
RBP4E_num2	102.0756	84320.75084	826.0617703	84320.75084	826.0617703
RBP4E_num3	25.5609	84320.75084	3298.817759	84320.75084	3298.817759
NTD2_num1	25.3006	9722.766341	384.2899513	9722.766341	384.2899513
NTD2_num2	26.9004	9722.766341	361.4357534	9722.766341	361.4357534
NTD2_num3	27.1631	9722.766341	357.9402329	9722.766341	357.9402329
NTD4_num1	26.9004	25068.67115	931.9070034	25068.67115	931.9070034
NTD4_num2	27.1631	25068.67115	922.8943366	25068.67115	922.8943366
NTD5_num1	45.4161	10199.23647	224.5731463	10199.23647	224.5731463
NTD5_num2	26.9004	10199.23647	379.1481343	10199.23647	379.1481343
NTD5_num3	25.3006	10199.23647	403.1223161	10199.23647	403.1223161
NTD5_num4	20.2554	10199.23647	503.5317235	10199.23647	503.5317235
NTD6_num1	45.4161	14096.05511	310.3757282	14096.05511	310.3757282
NTD6_num2	26.9004	14096.05511	524.0091266	14096.05511	524.0091266

NTD6_num3	20.2554	14096.05511	695.9159094	14096.05511	695.9159094
LGTHL	29.8397	9557.43858	320.2927167	9557.43858	320.2927167
PYDXPP_num1	30.2013	29369.90364	972.4715042	29369.90364	972.4715042
PYDXPP_num2	30.3709	29369.90364	967.0409386	29369.90364	967.0409386
PYDXPP_num3	29.7077	29369.90364	988.6293332	29369.90364	988.6293332
PYDXPP_num4	27.1631	29369.90364	1081.242702	29369.90364	1081.242702
PDXPP	30.2013	22698.00031	751.5570624	22698.00031	751.5570624
HACD6_num1	159.1878	31487.30375	197.7997293	31487.30375	197.7997293
HACD6_num2	77.0722	31487.30375	408.5429474	31487.30375	408.5429474
ECOAH1_num1	77.0722	43149.26455	559.8551041	43149.26455	559.8551041
ECOAH1_num2	159.1878	43149.26455	271.058866	43149.26455	271.058866
ECOAH2_num1	77.0722	48500.51292	629.2867327	48500.51292	629.2867327
ECOAH2_num2	159.1878	48500.51292	304.6748113	48500.51292	304.6748113
ECOAH3_num1	77.0722	44125.78766	572.525342	44125.78766	572.525342
ECOAH3_num2	159.1878	44125.78766	277.1932753	44125.78766	277.1932753
UGLT	79.2914	67891.37389	856.2261972	67891.37389	856.2261972
DXPS	135.2339	3960	29.28259852	3960	29.28259852
CDPMEK	61.8507	4680	75.66607977	4680	75.66607977
MECDPS	50.6926	324	6.391465421	324	6.391465421
DDGLK	33.9624	171581.3307	5052.096752	171581.3307	5052.096752
XYLI2	198.968	37166.12269	186.7944729	37166.12269	186.7944729
PMANM	50.4626	28985.11675	574.3880964	28985.11675	574.3880964

ICL	190.0863	58476.22074	307.6298541	58476.22074	307.6298541
GLYCL	364.0876	36542.3134	100.3668167	36542.3134	100.3668167
HCYSMT	33.4228	39542.54746	1183.100981	39542.54746	1183.100981
UDPG4E	74.5302	13617.81289	182.7153677	13617.81289	182.7153677
LACZ	465.9314	74153.44238	159.1509874	74153.44238	159.1509874
TRE6PH	63.8377	31368.07446	491.3722528	31368.07446	491.3722528
XYLI1	198.968	23950.64821	120.3743728	23950.64821	120.3743728
PPC	396.2504	165600	417.9175592	3596400	9076.079166
G3PT_num1	30.4128	9873.825329	324.6601868	9873.825329	324.6601868
G3PT_num2	27.1631	9873.825329	363.5014166	9873.825329	363.5014166
CINNDO	126.9954	34531.83732	271.9140797	34531.83732	271.9140797
DHPPD	28.4996	16735.64013	587.2236848	16735.64013	587.2236848
DHCIND	28.4996	14890.46441	522.4797684	14890.46441	522.4797684
CITL	614.1003	144633.9099	235.5216401	144633.9099	235.5216401
GOFUCR	72.2823	10022.30239	138.6550012	10022.30239	138.6550012
PYDXK_num1	61.6948	9301.044856	150.7589757	9301.044856	150.7589757
PYDXK_num2	62.6444	9301.044856	148.4736841	9301.044856	148.4736841
GTPCII2	43.672	432	9.891921597	432	9.891921597
SADT2	350.9871	102441.8063	291.8677247	102441.8063	291.8677247
LCARS	81.0267	12525.20682	154.5812285	12525.20682	154.5812285
ALCD19_num1	141.5183	66988.94494	473.3588867	66988.94494	473.3588867
ALCD19_num2	3845.0896	66988.94494	17.42194641	66988.94494	17.42194641

RMPA	120.582	25917.542	214.9370719	25917.542	214.9370719
SBTPD	111.4315	16357.75901	146.7965433	16357.75901	146.7965433
PFK_2	139.3682	28729.97065	206.1443762	28729.97065	206.1443762
GLTPD	74.7803	22429.04508	299.9325368	22429.04508	299.9325368
TGBPA_num1	172.3671	31362.76788	181.9533303	31362.76788	181.9533303
TGBPA_num2	77.9206	31362.76788	402.4964885	31362.76788	402.4964885
MANAO	53.5802	42550.17941	794.1399884	42550.17941	794.1399884
TAGURr	54.8083	28666.34973	523.0293538	28666.34973	523.0293538
PGLYCP	27.3892	10590.84588	386.6796359	10590.84588	386.6796359
GLXCL	129.4635	50871.83922	392.9434877	50871.83922	392.9434877
TARTD	110.826	40271.4679	363.3756329	40271.4679	363.3756329
GLCRAL	164.305	24765.1176	150.7265001	24765.1176	150.7265001
KAS14_num1	85.2266	9362.983381	109.8598722	9362.983381	109.8598722
KAS14_num2	86.0915	9362.983381	108.7561883	9362.983381	108.7561883
EDTXS1	35.4068	12691.75649	358.4553387	12691.75649	358.4553387
EDTXS2	37.4104	22707.38756	606.980614	22707.38756	606.980614
EDTXS3	35.4934	16672.68023	469.7402963	16672.68023	469.7402963
EDTXS4	37.4104	63673.51745	1702.027176	63673.51745	1702.027176
NNDPR	65.5241	1656	25.27314377	1656	25.27314377
HACD4_num1	159.1878	38552.38416	242.1817762	38552.38416	242.1817762
HACD4_num2	77.0722	38552.38416	500.2112845	38552.38416	500.2112845
HACD7_num1	77.0722	34363.09202	445.8558601	34363.09202	445.8558601

HACD7_num2	159.1878	34363.09202	215.8651104	34363.09202	215.8651104
AACPS1	89.3395	9300.085484	104.0982486	9300.085484	104.0982486
SDPDS	82.538	1540800	18667.76515	1540800	18667.76515
DMSOR1_num1	233.0425	51527.57551	221.1080619	51527.57551	221.1080619
DMSOR1_num2	144.0938	51527.57551	357.5974505	51527.57551	357.5974505
DKGLCNR2y	70.791	17120.40057	241.8443103	17120.40057	241.8443103
2DGULRy	70.791	19562.41623	276.340442	19562.41623	276.340442
RZ5PP	23.3085	14207.16538	609.5272273	14207.16538	609.5272273
NTD9_num1	20.2554	9725.196348	480.1285755	9725.196348	480.1285755
NTD9_num2	26.9004	9725.196348	361.5260869	9725.196348	361.5260869
NTD9_num3	25.3988	9725.196348	382.8998357	9725.196348	382.8998357
NTD9_num4	27.1631	9725.196348	358.0296928	9725.196348	358.0296928
NTD10	26.9004	9327.137796	346.7285912	9327.137796	346.7285912
ADPT	39.7178	10116.3237	254.7050365	10116.3237	254.7050365
ADK1	23.586	111600	4731.620453	111600	4731.620453
OCTDPS	70.4339	1080	15.33352548	1080	15.33352548
AACPS2	89.3395	9300.082612	104.0982165	9300.082612	104.0982165
AACPS3	89.3395	20818.49733	233.0267948	20818.49733	233.0267948
AACPS4	89.3395	19801.47101	221.6429576	19801.47101	221.6429576
AMANK	29.6439	18940.18921	638.9236642	18940.18921	638.9236642
ACNML	130.3739	22534.19094	172.8428078	22534.19094	172.8428078
CAT_num1	336.6504	190740.2702	566.5826335	190740.2702	566.5826335
CAT_num2	320.0953	190740.2702	595.8858821	190740.2702	595.8858821
SERASr	141.99	58615.06414	412.8083057	58615.06414	412.8083057

	1				
DMSOR2	144.09 38	94768.54027	657.6864534	94768.54027	657.6864534
ACMAMUT	27.928 2	20034.19254	717.3463574	20034.19254	717.3463574
DDGALK	31.373 6	25598.32552	815.9192928	25598.32552	815.9192928
AB6PGH_num1	53.161 3	42497.06141	799.3984611	42497.06141	799.3984611
AB6PGH_num2	55.360 8	42497.06141	767.6381376	42497.06141	767.6381376
AB6PGH_num3	53.934 9	42497.06141	787.9325151	42497.06141	787.9325151
AMALT1	78.503 5	57278.02975	729.623899	57278.02975	729.623899
MLTP2	181.04 48	27243.36347	150.4785747	27243.36347	150.4785747
PACCOAL	48.952 9	21124.09373	431.51874	21124.09373	431.51874
GP4GH	31.296 7	10828.74747	346.0028522	10828.74747	346.0028522
ACGAMT	81.914 5	10386.84518	126.801057	10386.84518	126.801057
AADDGT	40.639 6	231887.7737	5705.956103	231887.7737	5705.956103
GMHEPK	102.10 12	1502082.994	14711.70754	1502082.994	14711.70754
TRE6PS	53.611 2	26430.17295	492.9972272	26430.17295	492.9972272
UDCPDP_num1	29.021 3	15096.38615	520.1829743	15096.38615	520.1829743
UDCPDP_num2	22.398 6	15096.38615	673.9879346	15096.38615	673.9879346
UDCPDP_num3	29.758 8	15096.38615	507.2914954	15096.38615	507.2914954
TREH	63.696 7	19724.51893	309.6631211	19724.51893	309.6631211
CYTK1	24.746 3	86400	3491.431042	86400	3491.431042
CYTK2	24.746 3	25750.10172	1040.563709	25750.10172	1040.563709
GK1	47.185 4	151200	3204.381016	151200	3204.381016

HACD1_num1	159.1878	38780.61833	243.6155178	38780.61833	243.6155178
HACD1_num2	77.0722	38780.61833	503.1725879	38780.61833	503.1725879
HACD2_num1	159.1878	39035.14214	245.214408	39035.14214	245.214408
HACD2_num2	77.0722	39035.14214	506.4749954	39035.14214	506.4749954
ACACCT_num1	92.9717	27123.73889	291.7418838	27123.73889	291.7418838
ACACCT_num2	57.5623	27123.73889	471.206656	27123.73889	471.206656
BUTCT	92.9717	46897.97526	504.4328033	46897.97526	504.4328033
2DGULRx	70.791	20094.4938	283.8566174	20094.4938	283.8566174
G1PTT_num1	130.7742	10400.149	79.52752914	10400.149	79.52752914
G1PTT_num2	130.9376	10400.149	79.42828493	10400.149	79.42828493
TDPGDH_num1	39.7538	24678.10401	620.7734608	24678.10401	620.7734608
TDPGDH_num2	81.1167	24678.10401	304.2296347	24678.10401	304.2296347
UDPGALM	42.9659	651626.1936	15166.12462	651626.1936	15166.12462
TDPDRE	21.2701	18583.91096	873.7105588	18583.91096	873.7105588
TDPDRR	32.6941	14288.31244	437.0303032	14288.31244	437.0303032
G3PD6	148.4235	29478.25323	198.6090695	29478.25323	198.6090695
ALLTN	198.4064	150250.7634	757.2878869	150250.7634	757.2878869
UAPGR	37.851	154800	4089.720219	154800	4089.720219
NDPK7_num1	23.586	9921.020926	420.6317699	9921.020926	420.6317699
NDPK7_num2	61.8538	9921.020926	160.3946876	9921.020926	160.3946876
GLYAT	86.2341	10460.27884	121.3009568	10460.27884	121.3009568
ADNUC	32.5608	45598.68659	1400.416654	45598.68659	1400.416654
GMHEPPA	21.2942	283427.0473	13310.05848	283427.0473	13310.05848

S7PI	83.259	32534.6772	390.7646885	32534.6772	390.7646885
TRPS3	143.4143	35739.90722	249.2074167	35739.90722	249.2074167
CRNBTCT	90.2535	21541.32361	238.6757701	21541.32361	238.6757701
CRNCBCT	90.2535	22065.27	244.4810451	22065.27	244.4810451
CRNCDH	28.1902	39436.11997	1398.930124	39436.11997	1398.930124
FADRx	26.242	1090800	41566.95374	1090800	41566.95374
GTPDPK	83.8756	21923.85937	261.3854252	21923.85937	261.3854252
NDPK2_num1	23.586	24602.14215	1043.082428	24602.14215	1043.082428
NDPK2_num2	61.8538	24602.14215	397.7466566	24602.14215	397.7466566
NDPK3_num1	61.8538	17317.19681	279.9698129	17317.19681	279.9698129
NDPK3_num2	23.586	17317.19681	734.2150773	17317.19681	734.2150773
NDPK4_num1	23.586	12739.59929	540.1339477	12739.59929	540.1339477
NDPK4_num2	61.8538	12739.59929	205.9630822	12739.59929	205.9630822
FCLT_num1	33.0523	9524.036689	288.1504975	9524.036689	288.1504975
FCLT_num2	35.8844	9524.036689	265.4088319	9524.036689	265.4088319
FCLT_num3	93.5084	9524.036689	101.8522046	9524.036689	101.8522046
HBZOPT	32.5118	9461.03602	291.0031441	9461.03602	291.0031441
PNTK	72.7196	972	13.36641016	972	13.36641016
DPCOAK	22.6217	2304	101.8491095	2304	101.8491095
HETZK	54.6783	11542.76602	211.1032351	11542.76602	211.1032351
KDOPS	123.3308	2592	21.01664791	2592	21.01664791
KDOPP	79.9884	36000	450.0652595	36000	450.0652595
AST	38.4559	24033.07158	624.9514789	24033.07158	624.9514789
SGDS	35.8002	76498.5343	2136.818629	76498.5343	2136.818629

CYNTAH	170.4884	18024.11929	105.7205023	18024.11929	105.7205023
GSPMDA	141.0639	57857.65322	410.1520887	57857.65322	410.1520887
GSPMDS	141.0639	48666.39977	344.9954224	48666.39977	344.9954224
GUAD	50.2442	142271.6549	2831.603546	142271.6549	2831.603546
TMAOR1_num1	144.0938	100063.9026	694.4358644	100063.9026	694.4358644
TMAOR1_num2	233.0425	100063.9026	429.3804888	100063.9026	429.3804888
TMAOR2	144.0938	42205.22924	292.9010772	42205.22924	292.9010772
DGK1	47.1854	9898.287581	209.7743705	9898.287581	209.7743705
DTMPK	23.7831	64800	2724.623787	64800	2724.623787
NDPK1_num1	23.586	22045.90831	934.7031422	22045.90831	934.7031422
NDPK1_num2	61.8538	22045.90831	356.4196268	22045.90831	356.4196268
TRDR_num1	81.0527	36024.44087	444.4570121	36024.44087	444.4570121
TRDR_num2	84.8008	36024.44087	424.812512	36024.44087	424.812512
PGMT_num1	58.361	111558.8515	1911.530842	111558.8515	1911.530842
PGMT_num2	20.7798	111558.8515	5368.620078	111558.8515	5368.620078
PGMT_num3	23.5649	111558.8515	4734.110966	111558.8515	4734.110966
FRUK	67.5114	65166.15067	965.2614325	65166.15067	965.2614325
RHCCE	19.4162	10.8	0.556236545	10.8	0.556236545
KG6PDC_num1	47.1562	21707.18087	460.3250658	21707.18087	460.3250658
KG6PDC_num2	23.4448	21707.18087	925.8846681	21707.18087	925.8846681
X5PL3E_num1	32.0066	240986.0357	7529.260705	240986.0357	7529.260705
X5PL3E_num2	32.4552	240986.0357	7425.190284	240986.0357	7425.190284
DOGULNR	73.145	22733.58578	310.7999379	22733.58578	310.7999379

	4				
BETALDHx	211.6445	18043.37702	85.25322897	18043.37702	85.25322897
BETALDHy	211.6445	21034.4954	99.38597696	21034.4954	99.38597696
MLTG3	69.041	48889.46887	708.1222588	48889.46887	708.1222588
DKGLCNR1_num1	31.1096	10057.9245	323.3061338	10057.9245	323.3061338
DKGLCNR1_num2	29.4368	10057.9245	341.6785962	10057.9245	341.6785962
NADTRHD	412.4831	94594.50652	229.3294114	94594.50652	229.3294114
FMNRx2_num1	42.5067	17654.45437	415.3334504	17654.45437	415.3334504
FMNRx2_num2	26.242	17654.45437	672.7556731	17654.45437	672.7556731
FMNRx2_num3	530.1587	17654.45437	33.30032003	17654.45437	33.30032003
ACPS1_num1	28.1046	21613.67893	769.0441755	21613.67893	769.0441755
ACPS1_num2	21.7678	21613.67893	992.9197684	21613.67893	992.9197684
GMPS2	117.3584	18000	153.3763241	18000	153.3763241
AMPN	323.9695	22311.57137	68.86935766	22311.57137	68.86935766
GSNK	96.8976	69004.44076	712.1377697	69004.44076	712.1377697
ADCS	71.7414	9527.577685	132.8044572	9527.577685	132.8044572
ADCL_num1	59.4301	5040	84.80551101	5040	84.80551101
ADCL_num2	18.7769	5040	268.4149141	5040	268.4149141
DHAPT	360.8342	81935.07541	227.0712571	81935.07541	227.0712571
UMPK_num1	24.7463	42025.02012	1698.234488	42025.02012	1698.234488
UMPK_num2	155.8216	42025.02012	269.6995803	42025.02012	269.6995803
DDPGALA	21.3906	25821.72449	1207.152884	25821.72449	1207.152884
DURIK1	23.4566	13012.21128	554.7356087	13012.21128	554.7356087

UPPRT	67.5998	10152.73567	150.1888418	10152.73567	150.1888418
CTPS2	241.4966	39600	163.9774639	39600	163.9774639
NTD1_num1	45.4161	18381.74158	404.7406443	18381.74158	404.7406443
NTD1_num2	26.9004	18381.74158	683.3259571	18381.74158	683.3259571
NTD1_num3	25.3006	18381.74158	726.5338204	18381.74158	726.5338204
NTD1_num4	20.2554	18381.74158	907.4983252	18381.74158	907.4983252
NTD7_num1	25.3988	9716.829343	382.5704105	9716.829343	382.5704105
NTD7_num2	26.9004	9716.829343	361.2150505	9716.829343	361.2150505
NTD7_num3	20.2554	9716.829343	479.7155002	9716.829343	479.7155002
NTD7_num4	27.1631	9716.829343	357.7216644	9716.829343	357.7216644
PAPPT3	39.8748	252000	6319.780914	252000	6319.780914
UAGPT3	37.8147	61200	1618.418234	61200	1618.418234
F6PA_num1	229.9682	67194.11527	292.1887255	67194.11527	292.1887255
F6PA_num2	23.5552	67194.11527	2852.623424	67194.11527	2852.623424
AGMHE	174.4658	38346.39454	219.7931889	38346.39454	219.7931889
GMHEPAT	102.1012	348334.2784	3411.657046	348334.2784	3411.657046
IDOND	37.1466	19621.3609	528.2141812	19621.3609	528.2141812
IDOND2	37.1466	9973.965222	268.5027761	9973.965222	268.5027761
GLYCLTDx_num1	70.791	20514.68636	289.792295	20514.68636	289.792295
GLYCLTDx_num2	35.3434	20514.68636	580.4389605	20514.68636	580.4389605
HPYRRy_num1	35.3434	9598.25048	271.5712263	9598.25048	271.5712263
HPYRRy_num2	70.791	9598.25048	135.5857451	9598.25048	135.5857451

ACODA	84.6947	93600	1105.145895	93600	1105.145895
ADA	36.3975	38158.03206	1048.369587	38158.03206	1048.369587
DADA	36.3975	37137.1001	1020.32008	37137.1001	1020.32008
ADD	127.4787	96905.20187	760.1677917	96905.20187	760.1677917
ADSS	94.6899	11880	125.4621665	11880	125.4621665
AGDC	163.7964	112916.4612	689.3708358	112916.4612	689.3708358
ASNN_num1	148.509	12284.90508	82.7216201	12284.90508	82.7216201
ASNN_num2	33.394	12284.90508	367.8776151	12284.90508	367.8776151
ASNS2_num1	73.3011	68400	933.137429	68400	933.137429
ASNS2_num2	125.318	68400	545.8114557	68400	545.8114557
ASPCT_num1	309.2879	34791.89775	112.4903294	34791.89775	112.4903294
ASPCT_num2	206.5642	34791.89775	168.4314017	34791.89775	168.4314017
DMPPS	69.5491	396	5.693819187	396	5.693819187
MCOATA	41.0567	17706.54298	431.2704864	17706.54298	431.2704864
IPDPS	69.5491	3096	44.51531364	3096	44.51531364
DAPE	60.4171	302400	5005.20548	302400	5005.20548
DHDPRy	115.0264	50400	438.1602832	50400	438.1602832
HSK	67.2473	291600	4336.233574	291600	4336.233574
SDPTA_num1	91.5493	17299.46712	188.9634013	17299.46712	188.9634013
SDPTA_num2	44.7291	17299.46712	386.7609032	17299.46712	386.7609032
SDPTA_num3	87.5339	17299.46712	197.6316275	17299.46712	197.6316275
SDPTA_num4	87.3307	17299.46712	198.0914743	17299.46712	198.0914743

SDPTA_num5	79.5666	17299.46712	217.4212184	17299.46712	217.4212184
THRS	47.1138	14400	305.6429326	14400	305.6429326
HSST	71.4546	28800	403.0531274	28800	403.0531274
PPNDH	86.2227	43200	501.0281515	43200	501.0281515
DDPA_num1	152.0381	140719.9588	925.5572045	140719.9588	925.5572045
DDPA_num2	77.6079	140719.9588	1813.216938	140719.9588	1813.216938
DDPA_num3	77.4701	140719.9588	1816.442199	140719.9588	1816.442199
NMNDA	35.1634	23640.84149	672.3138686	23640.84149	672.3138686
NNAM	23.362	11380.49961	487.1372148	11380.49961	487.1372148
P5CD	287.6303	176721.467	614.4049046	176721.467	614.4049046
SPMDAT1	87.548	32401.65086	370.1015541	32401.65086	370.1015541
SPMDAT2	87.548	18678.29696	213.3492136	18678.29696	213.3492136
TDPADGAT	24.2204	27137.47252	1120.43866	27137.47252	1120.43866
MALS_num1	60.2736	84473.14158	1401.494876	84473.14158	1401.494876
MALS_num2	80.4885	84473.14158	1049.505725	84473.14158	1049.505725
TRE6PP	29.1753	9577.301641	328.2674605	9577.301641	328.2674605
ADPRDP_num1	42.3063	13031.08768	308.0176637	13031.08768	308.0176637
ADPRDP_num2	47.3336	13031.08768	275.3031184	13031.08768	275.3031184
ARAI	336.4439	46557.9562	138.3825244	46557.9562	138.3825244
M1PD	41.139	21414.05704	520.5293526	21414.05704	520.5293526
G3PD2	72.7232	68644.24348	943.9112068	68644.24348	943.9112068
GALS3	101.3141	27314.25764	269.5997659	27314.25764	269.5997659
FCI	389.86	38459.23614	98.6488384	38459.23614	98.6488384
FCLK	52.2587	68205.67506	1305.154454	68205.67506	1305.154454

FCLPA_num1	23.2225	24648.80337	1061.419028	24648.80337	1061.419028
FCLPA_num2	95.1013	24648.80337	259.1847154	24648.80337	259.1847154
ACCOAL_num1	72.0936	16156.30072	224.1017332	16156.30072	224.1017332
ACCOAL_num2	69.3509	16156.30072	232.9645429	16156.30072	232.9645429
GALUi	131.769	10809.57201	82.034257	10809.57201	82.034257
U23GAAT	108.1154	129600	1198.719146	129600	1198.719146
UAAGDS	53.3436	28800	539.89607	28800	539.89607
UAMAS	107.2519	50400	469.921745	50400	469.921745
UAMAGS	46.9736	104400	2222.524993	104400	2222.524993
UGLYCH	18.1696	91417.25945	5031.330324	91417.25945	5031.330324
OP4ENH	144.4494	26173.1843	181.1927519	26173.1843	181.1927519
HOPNTAL	36.4704	50874.6321	1394.95679	50874.6321	1394.95679
3HPPPNH	62.1857	16534.68041	265.8920042	16534.68041	265.8920042
AKGDH	2418.391	112697.4534	46.60017897	3596400	1487.104443
HPYRRx_num1	35.3434	16797.03153	475.2522829	16797.03153	475.2522829
HPYRRx_num2	70.791	16797.03153	237.2763704	16797.03153	237.2763704
GLYCDx	309.6976	19473.16795	62.87800728	19473.16795	62.87800728
ASP1DC	13.8337	252	18.21638463	252	18.21638463
UGMDDS	47.4472	57600	1213.981015	57600	1213.981015
UHGADA	33.9559	75600	2226.417206	75600	2226.417206
UAGAAT	92.8795	17547.89693	188.9318625	17547.89693	188.9318625
ATPPRT	66.7334	7920	118.6812001	7920	118.6812001

HISTD	92.2207	11160	121.0140457	11160	121.0140457
IGPDH	80.5559	13680	169.8199635	13680	169.8199635
PRPPS	34.2183	28800	841.654904	28800	841.654904
PRATPP	22.7558	79200	3480.431363	79200	3480.431363
PRMICI	26.0326	36000	1382.881464	36000	1382.881464
SERAT	175.8998	136800	777.7154948	136800	777.7154948
CYSS_num1	65.3283	47534.80306	727.6295733	47534.80306	727.6295733
CYSS_num2	68.9793	47534.80306	689.1169243	47534.80306	689.1169243
BPNT	27.1759	79200	2914.346903	79200	2914.346903
PAPSR_num1	71.5064	32950.32997	460.8025291	32950.32997	460.8025291
PAPSR_num2	67.7583	32950.32997	486.2921586	32950.32997	486.2921586
ASPK_num1	177.7754	631361.1827	3551.454153	631361.1827	3551.454153
ASPK_num2	356.481	631361.1827	1771.093502	631361.1827	1771.093502
ASPK_num3	97.0635	631361.1827	6504.619993	631361.1827	6504.619993
ASAD	80.0358	29917.33841	373.7994549	29917.33841	373.7994549
DAPDC	92.3545	144000	1559.209351	144000	1559.209351
METAT	167.8065	21.6	0.128719686	27000	160.8996076
ASCBPL	240.364	38846.75555	161.6163633	38846.75555	161.6163633
ACBIPGT	40.328	9379.708193	232.5855037	9379.708193	232.5855037
ADOCBLS	26.3856	10035.97058	380.3578686	10035.97058	380.3578686
ADOCBIK	40.328	9323.058784	231.1807871	9323.058784	231.1807871
TMPK	35.0708	828	23.60938445	828	23.60938445
HPPPNDO	136.78	35492.10059	259.4751484	35492.10059	259.4751484

	42				
DHCINDO	136.78<br>42	19495.60768	142.5282137	19495.60768	142.5282137
HKNDDH	63.874<br>9	38363.58101	600.6049482	38363.58101	600.6049482
HKNTDH	63.874<br>9	18327.29297	286.924801	18327.29297	286.924801
GLYCK2	39.104<br>3	61745.26025	1578.989018	61745.26025	1578.989018
ENTCS	23.258<br>6	10772.53122	463.1633554	10772.53122	463.1633554
SULR	786.15<br>12	71599.20646	91.07561809	71599.20646	91.07561809
MANPGH	100.01<br>5	80637.61097	806.2551714	80637.61097	806.2551714
URDGLYCD	75.934<br>2	10345.53972	136.2434808	10345.53972	136.2434808
CBMKr	31.644<br>1	37048.79484	1170.796289	37048.79484	1170.796289
AMANAPEr	24.073<br>7	25940.03066	1077.525709	25940.03066	1077.525709
ACM6PH	62.439<br>8	12910.85677	206.772872	12910.85677	206.772872
NNATr	24.528	15120	616.4383562	15120	616.4383562
CYTDH_num1	33.822<br>9	23757.87457	702.4197975	23757.87457	702.4197975
CYTDH_num2	134.99<br>2	23757.87457	175.9946854	23757.87457	175.9946854
CYTDH_num3	32.560<br>8	23757.87457	729.6465249	23757.87457	729.6465249
PSERT	79.566<br>6	29880	375.5344579	29880	375.5344579
AIRC2	78.922<br>2	554400	7024.639455	554400	7024.639455
GAPD	142.13	104400	734.5388025	3596400	25303.5953
HEX1	34.723<br>1	47562.8567	1369.775645	47562.8567	1369.775645
GND	102.96<br>26	79200	769.2113447	1170000	11363.34941
TAUDO	129.63<br>81	9422.360968	72.68203536	9422.360968	72.68203536
ME2	494.50<br>43	502505.1652	1016.179566	502505.1652	1016.179566

ARGSL	50.3182	39600	786.9915855	39600	786.9915855
CBPS	318.5455	12764.65483	40.07168466	12764.65483	40.07168466
GLU5K	156.2261	90000	576.0881184	90000	576.0881184
IPPS	57.2979	36000	628.2952778	36000	628.2952778
ACOATA	75.6698	32149.38905	424.8642001	32149.38905	424.8642001
MLTG2	69.041	44577.3078	645.6642835	44577.3078	645.6642835
MLTG1	69.041	64611.20243	935.8381603	64611.20243	935.8381603
AMALT3	78.5035	26398.09512	336.2664737	26398.09512	336.2664737
MLTP3	181.0448	21419.81586	118.3122402	21419.81586	118.3122402
SPODM_num1	46.1942	43146.08622	934.0152275	43146.08622	934.0152275
SPODM_num2	42.5316	43146.08622	1014.447757	43146.08622	1014.447757
AHCYSNS	48.7079	108	2.217299452	108	2.217299452
TMK	32.3973	9301.67635	287.1127023	9301.67635	287.1127023
NTPP4_num1	15.0461	9555.079144	635.053545	9555.079144	635.053545
NTPP4_num2	60.8246	9555.079144	157.0923466	9555.079144	157.0923466
NTPP3_num1	60.8246	50618.48846	832.2042145	50618.48846	832.2042145
NTPP3_num2	16.3707	50618.48846	3092.017352	50618.48846	3092.017352
NTPP3_num3	15.0461	50618.48846	3364.226508	50618.48846	3364.226508
NTPP6_num1	60.8246	10261.67914	168.7093568	10261.67914	168.7093568
NTPP6_num2	65.6516	10261.67914	156.3050884	10261.67914	156.3050884
NTPP7_num1	16.3707	9517.851461	581.3955091	9517.851461	581.3955091
NTPP7_num2	60.8246	9517.851461	156.4802968	9517.851461	156.4802968
NTPP7_num3	43.030	9517.851461	221.1864372	9517.851461	221.1864372

	9				
NTPP8_num1	60.824 6	9521.20381	156.5354118	9521.20381	156.5354118
NTPP8_num2	43.030 9	9521.20381	221.2643428	9521.20381	221.2643428
P5CR	281.44 88	115200	409.310681	115200	409.310681
CYANST_num1	37.277 5	331771.8227	8900.055601	331771.8227	8900.055601
CYANST_num2	24.164 9	331771.8227	13729.49289	331771.8227	13729.49289
CYANST_num3	30.811 8	331771.8227	10767.68714	331771.8227	10767.68714
DHPPDA2	80.676 6	1152	14.27923338	1152	14.27923338
GMAND	84.093 7	18347.65327	218.181068	18347.65327	218.181068
PPCSCT	53.824 4	15747.6871	292.5752466	15747.6871	292.5752466
CYTD	63.079 7	65944.35572	1045.413274	65944.35572	1045.413274
DCYTD	63.079 7	58808.63711	932.2910082	58808.63711	932.2910082
DBTS	48.279 2	1.8	0.037283136	1.8	0.037283136
DCTPD	63.747 8	10867.18117	170.47147	10867.18117	170.47147
DHDPS	125.07 99	39600	316.5976308	39600	316.5976308
G1PACT	147.57 02	57600	390.3227074	57600	390.3227074
GART	42.433 7	432	10.1805876	432	10.1805876
CSND	285.54 59	15341.80556	53.72798405	15341.80556	53.72798405
GF6PTA	133.78 87	7920	59.19782463	7920	59.19782463
GLUCYS	58.269 3	24739.04104	424.5638962	24739.04104	424.5638962
GLUN_num1	131.61 33	43731.73602	332.2744435	43731.73602	332.2744435
GLUN_num2	67.031 2	43731.73602	652.4086697	43731.73602	652.4086697

GLUPRT	225.9522	219600	971.8869743	219600	971.8869743
GMPR	149.5347	11043.44409	73.85204965	11043.44409	73.85204965
NADS1	61.2737	180	2.937638824	180	2.937638824
NTRIR2x	105.4053	194253.4062	1842.918773	194253.4062	1842.918773
OCBT_num1	110.7213	45832.3358	413.9432594	45832.3358	413.9432594
OCBT_num2	110.4814	45832.3358	414.8420983	45832.3358	414.8420983
GARFT	23.2383	403200	17350.66679	403200	17350.66679
GPDDA1	27.4096	10438.37512	380.8291664	10438.37512	380.8291664
GPDDA2	27.4096	9300.301081	339.3081651	9300.301081	339.3081651
PHETA1_num1	87.1467	163144.9555	1872.072672	163144.9555	1872.072672
PHETA1_num2	87.0756	163144.9555	1873.601279	163144.9555	1873.601279
PHETA1_num3	204.5619	163144.9555	797.5334386	163144.9555	797.5334386
PPND	84.085	79200	941.9040257	79200	941.9040257
TRPS1	143.4143	10429.37243	72.72198397	10429.37243	72.72198397
PRAMPC	22.7558	79200	3480.431363	79200	3480.431363
MCITL2	128.5386	38608.8206	300.3675208	38608.8206	300.3675208
DXYLK	105.2365	37749.01216	358.7064579	37749.01216	358.7064579
MDH3	60.2296	139494.2586	2316.04159	139494.2586	2316.04159
SOTA	87.3307	24429.86623	279.7397276	24429.86623	279.7397276
SGSAD	53.0263	28118.21406	530.2692072	28118.21406	530.2692072
PTA2	463.0325	89932.12319	194.2242136	89932.12319	194.2242136
MDH2	60.2296	42664.64483	708.3667304	42664.64483	708.3667304

ACOTA_num1	91.5493	17996.38411	196.5758789	17996.38411	196.5758789
ACOTA_num2	87.3307	17996.38411	206.0716805	17996.38411	206.0716805
ACOTA_num3	87.5339	17996.38411	205.5933085	17996.38411	205.5933085
ACOTA_num4	44.7291	17996.38411	402.3417442	17996.38411	402.3417442
GPDDA5	27.4096	10984.70162	400.7611064	10984.70162	400.7611064
PDX5PS	281.3019	9443.526638	33.57078867	9443.526638	33.57078867
PPBNGS	284.9983	864	3.031597031	864	3.031597031
PPNCL2	521.2577	540	1.03595592	540	1.03595592
PRAGSr	45.9404	72000	1567.248	72000	1567.248
PRFGS	141.4027	50400	356.4288376	50400	356.4288376
ETHAAL	487.1078	71428.12398	146.6372002	71428.12398	146.6372002
MAN1PT2	53.0163	174021.8972	3282.422524	174021.8972	3282.422524
AP4AH	31.2967	77148.74714	2465.076099	77148.74714	2465.076099
AP5AH	31.2967	42259.28724	1350.279334	42259.28724	1350.279334
ADNCYC	97.586	31838.44056	326.2603299	31838.44056	326.2603299
MI1PP	29.1721	404041.0784	13850.25687	404041.0784	13850.25687
MCITS	86.2044	35244.93913	408.8531343	35244.93913	408.8531343
PPAKr	43.3841	12000.38922	276.6080021	12000.38922	276.6080021
NTP3_num1	68.8674	24885.86102	361.3590904	24885.86102	361.3590904
NTP3_num2	36.7039	24885.86102	678.0168054	24885.86102	678.0168054
NTP3_num3	35.6597	24885.86102	697.8707343	24885.86102	697.8707343
NTP3_num4	39.1933	24885.86102	634.9519184	24885.86102	634.9519184

NTP3_num5	31.565	24885.86102	788.4004759	24885.86102	788.4004759
NTP3_num6	36.1132	24885.86102	689.1070584	24885.86102	689.1070584
NTP3_num7	48.3271	24885.86102	514.9462935	24885.86102	514.9462935
PPM2	44.37	146884.0402	3310.435884	146884.0402	3310.435884
MOAT2	47.2911	212400	4491.33135	212400	4491.33135
G6PDH2r	55.7044	558000	10017.16202	558000	10017.16202
NADH5_num1	40.7511	19111.97638	468.9928953	19111.97638	468.9928953
NADH5_num2	47.3587	19111.97638	403.5578759	19111.97638	403.5578759
NADH5_num3	40.34	19111.97638	473.7723445	19111.97638	473.7723445
NADH5_num4	83.3822	19111.97638	229.2093082	19111.97638	229.2093082
MOHMT	282.3744	36	0.127490311	36	0.127490311
GMPS	117.3584	24356.93407	207.5431675	24356.93407	207.5431675
UDPGD	87.3138	49328.26859	564.9538628	49328.26859	564.9538628
GLCRD_num1	48.8496	16806.78677	344.0516763	16806.78677	344.0516763
GLCRD_num2	49.141	16806.78677	342.0114928	16806.78677	342.0114928
PPPNDO	126.9954	20380.61275	160.4830785	20380.61275	160.4830785
CHRPL_num1	18.7769	828	44.09673588	828	44.09673588
CHRPL_num2	59.4301	828	13.93233395	828	13.93233395
TDPAGTA	167.6041	14344.14992	85.58352642	14344.14992	85.58352642
UACMAMO	91.6775	34516.73316	376.5016843	34516.73316	376.5016843
FBA3	78.2945	14400	183.9209651	14400	183.9209651
PMPK	114.5344	1800	15.71580241	1800	15.71580241
LCADi	209.0912	22726.49816	108.6917965	22726.49816	108.6917965
ADK3	23.586	10020.99778	424.8705918	10020.99778	424.8705918

GLUDC_num1	316.111	63196.41049	199.9184163	63196.41049	199.9184163
GLUDC_num2	316.0088	63196.41049	199.9830716	63196.41049	199.9830716
BSORy	85.8507	52282.98518	608.9989387	52282.98518	608.9989387
ADSK	44.6426	79200	1774.090219	79200	1774.090219
HSDy_num1	177.7754	70415.01259	396.0897435	70415.01259	396.0897435
HSDy_num2	356.481	70415.01259	197.5280943	70415.01259	197.5280943
CYSTL_num1	172.8495	16792.14941	97.14896145	16792.14941	97.14896145
CYSTL_num2	87.2837	16792.14941	192.3858568	16792.14941	192.3858568
SHSL1	166.2016	36000	216.604413	36000	216.604413
SSALy_num1	99.4357	30829.92795	310.0488854	30829.92795	310.0488854
SSALy_num2	206.8803	30829.92795	149.0230242	30829.92795	149.0230242
3HCINNMH	62.1857	13931.45469	224.0298764	13931.45469	224.0298764
BSORx	85.8507	11186.78545	130.3051164	11186.78545	130.3051164
FACOAL160t2pp_num1	60.7731	20734.33515	341.1761973	20734.33515	341.1761973
FACOAL160t2pp_num2	124.664	20734.33515	166.3217541	20734.33515	166.3217541
FACOAL180t2pp_num1	60.7731	19696.91639	324.1058361	19696.91639	324.1058361
FACOAL180t2pp_num2	124.664	19696.91639	158.0000352	19696.91639	158.0000352
SPODMpp	17.681	10789.89295	610.2535462	10789.89295	610.2535462
NO3R1bpp	189.2373	174653.5775	922.9342077	174653.5775	922.9342077
PHYTSpp	94.1137	38198.7126	405.8783429	38198.7126	405.8783429
PPTHpp	98.8772	6160798.267	62307.57209	6160798.267	62307.57209
NO3R2pp_num1	250.963	1547926.092	6167.945442	1547926.092	6167.945442

NO3R2pp_num2	474.5552	1547926.092	3261.846234	1547926.092	3261.846234
NTP3pp	94.1137	9514.245618	101.0930993	9514.245618	101.0930993
NO3R2bpp	132.4391	22948.30134	173.2743679	22948.30134	173.2743679
THD2pp	206.6929	2171332.143	10505.11238	2171332.143	10505.11238
TMAOR1pp_num1	138.0625	26365.14013	190.9652522	26365.14013	190.9652522
TMAOR1pp_num2	129.2504	26365.14013	203.984979	26365.14013	203.984979
TMAOR2pp_num1	138.0625	18727.86361	135.6477219	18727.86361	135.6477219
TMAOR2pp_num2	129.2504	18727.86361	144.8959818	18727.86361	144.8959818
ACACT7r_num1	46.5305	36389.7463	782.0622236	36389.7463	782.0622236
ACACT7r_num2	81.7524	36389.7463	445.1214435	36389.7463	445.1214435
PSP_L	35.0427	5331600	152145.8107	5331600	152145.8107
ACACT2r_num1	46.5305	45028.02067	967.7097962	45028.02067	967.7097962
ACACT2r_num2	81.7524	45028.02067	550.7853063	45028.02067	550.7853063
ACACT3r_num1	46.5305	35394.18648	760.666369	35394.18648	760.666369
ACACT3r_num2	81.7524	35394.18648	432.9436993	35394.18648	432.9436993
ACACT4r_num1	46.5305	41966.54604	901.9147879	41966.54604	901.9147879
ACACT4r_num2	81.7524	41966.54604	513.3371747	41966.54604	513.3371747
AHGDx	176.7031	23135.45055	130.9283796	23135.45055	130.9283796
GTPCI	248.3062	28.8	0.115985827	28.8	0.115985827
FDMO	166.9451	9726.121987	58.25940376	9726.121987	58.25940376
CHOLD	61.8776	32331.58284	522.5086759	32331.58284	522.5086759
DRPA	27.7338	57740.0409	2081.937596	57740.0409	2081.937596

TKT2_num1	144.4235	324065.5255	2243.85592	324065.5255	2243.85592
TKT2_num2	73.0427	324065.5255	4436.658632	324065.5255	4436.658632
DMSOR1pp	129.2504	32437.32175	250.9649622	32437.32175	250.9649622
NTD8pp_num1	104.4142	15863.73439	151.9308139	15863.73439	151.9308139
NTD8pp_num2	60.824	15863.73439	260.8137313	15863.73439	260.8137313
TREHpp	63.6367	42399.81511	666.2792871	42399.81511	666.2792871
CYTBDpp_num1	100.3437	49127.83924	489.5956521	49127.83924	489.5956521
CYTBDpp_num2	104.6999	49127.83924	469.2252737	49127.83924	469.2252737
CYTBD2pp	100.3437	28476.71077	283.7917156	28476.71077	283.7917156
ATPS4rpp_num1	543.6444	6754830.271	12425.0894	6754830.271	12425.0894
ATPS4rpp_num2	530.0127	6754830.271	12744.65739	6754830.271	12744.65739
ECOAH8_num1	77.0722	18863.03522	244.7449952	18863.03522	244.7449952
ECOAH8_num2	159.1878	18863.03522	118.4954828	18863.03522	118.4954828
GLCDpp_num1	86.7474	43571.8008	502.2836512	43571.8008	502.2836512
GLCDpp_num2	41.0545	43571.8008	1061.316075	43571.8008	1061.316075
HYD2pp_num1	482.9335	7210616.458	14930.86824	7210616.458	14930.86824
HYD2pp_num2	181.7482	7210616.458	39673.66091	7210616.458	39673.66091
ECOAH5_num1	77.0722	45024.42384	584.1850089	45024.42384	584.1850089
ECOAH5_num2	159.1878	45024.42384	282.8384075	45024.42384	282.8384075
DHFS	45.4057	828	18.23559597	828	18.23559597
ACGAMK	33.0427	21792.22839	659.5171821	21792.22839	659.5171821
METS_num1	135.997	1116	8.206063369	1116	8.206063369

METS_num2	84.6735	1116	13.18003862	3596400	42473.73736
PRASCSi	80.985	12600	155.5843675	12600	155.5843675
TRSARr_num1	123.2023	53910.27997	437.5752723	53910.27997	437.5752723
TRSARr_num2	30.4274	53910.27997	1771.767551	53910.27997	1771.767551
LYXI	188.7978	47448.98608	251.3217107	47448.98608	251.3217107
URIH_num1	33.8229	12358.14336	365.3779942	12358.14336	365.3779942
URIH_num2	134.992	12358.14336	91.54722769	12358.14336	91.54722769
URIH_num3	32.5608	12358.14336	379.5405322	12358.14336	379.5405322
XTSNH	32.5608	482024.5754	14803.83085	482024.5754	14803.83085
INSH	32.5608	36111.155	1109.037708	36111.155	1109.037708
PPK2	160.863	39038.03057	242.6787426	39038.03057	242.6787426
MTHFR2	132.4109	9720	73.40785389	9720	73.40785389
ASPO3	60.3374	10440.93368	173.0424857	10440.93368	173.0424857
ASPO5	60.3374	9646.312734	159.8728605	9646.312734	159.8728605
ASPO4	60.3374	9316.275055	154.4029914	9316.275055	154.4029914
ASPO6	60.3374	15120	250.5908442	15120	250.5908442
ACACT5r_num1	81.7524	48979.49998	599.1200255	48979.49998	599.1200255
ACACT5r_num2	46.5305	48979.49998	1052.632144	48979.49998	1052.632144
MALDDH	40.3149	21531.68437	534.0875054	21531.68437	534.0875054
DHPS2	61.2302	720	11.75890329	720	11.75890329
MTHFC	62.0875	248400	4000.805315	248400	4000.805315
MTHFD	62.0875	248400	4000.805315	248400	4000.805315

QULNS	76.4816	1656	21.65226669	1656	21.65226669
ALKP	20.7798	10071.37347	484.6713383	10071.37347	484.6713383
L_LACD3_num1	104.2689	61053.04188	585.534535	61053.04188	585.534535
L_LACD3_num2	42.7282	61053.04188	1428.869971	61053.04188	1428.869971
L_LACD2_num1	104.2689	93550.12704	897.200671	93550.12704	897.200671
L_LACD2_num2	42.7282	93550.12704	2189.423543	93550.12704	2189.423543
ACACT6r_num1	46.5305	40632.79266	873.2507207	40632.79266	873.2507207
ACACT6r_num2	81.7524	40632.79266	497.0226276	40632.79266	497.0226276
GLCTR1	42.2841	19615.95287	463.9084872	19615.95287	463.9084872
LIPAabcpp	128.9214	14115.54858	109.4895695	14115.54858	109.4895695
LIPACabcpp	128.9214	10250.91946	79.51293939	10250.91946	79.51293939
GGGABADr	53.4186	20624.58608	386.0937217	20624.58608	386.0937217
GLYOX3	62.3809	9301.150228	149.1025334	9301.150228	149.1025334
FDMO3	166.9451	19785.56126	118.5153758	19785.56126	118.5153758
FDMO4	166.9451	14910.81158	89.31565875	14910.81158	89.31565875
O16GLCT1	43.1882	28037.51536	649.1938854	28037.51536	649.1938854
O16AP2pp	81.199	12811.16978	157.7749699	12811.16978	157.7749699
O16AP3pp	81.199	16964.83849	208.9291554	16964.83849	208.9291554
O16A4Lpp	46.8777	21752.96452	464.0365146	21752.96452	464.0365146
MALTATr	60.288	33857.47486	561.5955889	33857.47486	561.5955889
ENTERES	45.6519	11061.15945	242.293518	11061.15945	242.293518
LADGMDH	53.116	25938.68088	488.340253	25938.68088	488.340253
ALAGLUE	34.674	73466.26981	2118.77112	73466.26981	2118.77112
GGGABAH	56.0261	5814339.581	103779.124	5814339.581	103779.124

FRULYSK	28.3318	26674.19432	941.493104	26674.19432	941.493104
FDMO6	166.9451	9703.843927	58.12595834	9703.843927	58.12595834
CYANSTpp	11.4753	10162.25352	885.5762833	10162.25352	885.5762833
MCPST	30.8118	11568.74394	375.4647225	11568.74394	375.4647225
ECAP1pp	135.966	53500.34426	393.4832551	53500.34426	393.4832551
UDCPDPpp_num1	29.7588	22302.93351	749.4567492	22302.93351	749.4567492
UDCPDPpp_num2	29.0213	22302.93351	768.5022211	22302.93351	768.5022211
UDCPDPpp_num3	22.3986	22302.93351	995.7289076	22302.93351	995.7289076
ECAP2pp	135.966	113152.0132	832.2081489	113152.0132	832.2081489
ECA4OALpp	46.8777	9951.766805	212.2921305	9951.766805	212.2921305
FE3Ri	26.242	41567.57582	1584.009444	41567.57582	1584.009444
NODOy	43.8677	18721.97778	426.782753	18721.97778	426.782753
GALM2pp	38.1905	52658.36885	1378.834235	52658.36885	1378.834235
GLCTR3	39.0403	14212.74192	364.0530919	14212.74192	364.0530919
HEPT4	41.7295	66050.8024	1582.832346	66050.8024	1582.832346
LA4NTpp	62.5426	30335.10919	485.0311498	30335.10919	485.0311498
NODOx	43.8677	34540.48398	787.3785034	34540.48398	787.3785034
NHFRBO	258.3403	76271.50746	295.2365831	76271.50746	295.2365831
42A12BOOXpp	168.7573	12329.96585	73.06330363	12329.96585	73.06330363
TYROXDApp	168.7573	12377.02951	73.34218731	12377.02951	73.34218731
NTRIR4pp	134.0271	752249.2273	5612.665105	752249.2273	5612.665105
NTRIR3pp	134.0271	1003797.469	7489.511216	1003797.469	7489.511216

AGM3PA	20.5362	24213.93207	1179.085326	24213.93207	1179.085326
ACANTHAT	64.5492	14042.58626	217.5485717	14042.58626	217.5485717
METSOXR1_num1	101.4054	9516.09821	93.84212488	9516.09821	93.84212488
METSOXR1_num2	38.8698	9516.09821	244.8198398	9516.09821	244.8198398
METSOXR1_num3	35.1216	9516.09821	270.9471724	9516.09821	270.9471724
METSOXR1_num4	97.6573	9516.09821	97.44379796	9516.09821	97.44379796
METSOXR2_num1	48.0499	9462.184355	196.9241217	9462.184355	196.9241217
METSOXR2_num2	31.006	9462.184355	305.1726877	9462.184355	305.1726877
METSOXR2_num3	27.2578	9462.184355	347.1367592	9462.184355	347.1367592
METSOXR2_num4	51.798	9462.184355	182.6747047	9462.184355	182.6747047
DAPAL	86.6551	33933.61467	391.5939705	33933.61467	391.5939705
AMPTASECG_num1	277.081	81038.05956	292.4706478	81038.05956	292.4706478
AMPTASECG_num2	98.9189	81038.05956	819.2373708	81038.05956	819.2373708
AMPTASECG_num3	329.2789	81038.05956	246.10766	81038.05956	246.10766
AMPTASECG_num4	105.8308	81038.05956	765.7322779	81038.05956	765.7322779
LACZpp	83.46	21454.23258	257.0600597	21454.23258	257.0600597
GPDDA1pp	81.6867	37449.60109	458.4540824	37449.60109	458.4540824
GPDDA5pp	81.6867	28550.56261	349.5129882	28550.56261	349.5129882
FLVR_num1	26.242	11168.07223	425.5800711	11168.07223	425.5800711
FLVR_num2	786.1512	11168.07223	14.20601053	11168.07223	14.20601053
FDMO2	166.9451	9825.639001	58.85550999	9825.639001	58.85550999
LALGP	105.8308	231021.2838	2182.930525	231021.2838	2182.930525
AGM4PApp_num	31.412	24410.50695	777.1052223	24410.50695	777.1052223

1	1				
AGM4PApp_num 2	45.634 4	24410.50695	534.9146029	24410.50695	534.9146029
AGM4PApp_num 3	47.985 2	24410.50695	508.7090802	24410.50695	508.7090802
AGM4PApp_num 4	31.072 4	24410.50695	785.6009498	24410.50695	785.6009498
AGM4PA	20.536 2	20099.44301	978.7323368	20099.44301	978.7323368
RNDR3b_num1	258.19 47	19613.29221	75.96318675	19613.29221	75.96318675
RNDR3b_num2	243.52 94	19613.29221	80.53767723	19613.29221	80.53767723
RNDR3b_num3	242.98 2	19613.29221	80.71911588	19613.29221	80.71911588
RNDR3b_num4	259.60 2	19613.29221	75.55139103	19613.29221	75.55139103
RNDR4b_num1	259.60 2	173713.9787	669.155009	173713.9787	669.155009
RNDR4b_num2	243.52 94	173713.9787	713.3183043	173713.9787	713.3183043
RNDR4b_num3	242.98 2	173713.9787	714.9252976	173713.9787	714.9252976
RNDR4b_num4	258.19 47	173713.9787	672.8022638	173713.9787	672.8022638
UDPGALPpp	60.824	15095.45394	248.1825256	15095.45394	248.1825256
UACGAMPpp	60.824	11672.57338	191.9073619	11672.57338	191.9073619
UACGALPpp	60.824	13846.87087	227.6547229	13846.87087	227.6547229
UGLCURPpp	60.824	14359.62176	236.0847982	14359.62176	236.0847982
FEROpp_num1	56.556 2	9485.933332	167.7257901	9485.933332	167.7257901
FEROpp_num2	443.88 07	9485.933332	21.37045682	9485.933332	21.37045682
AM4PCP	33.567 5	54473.38639	1622.801412	54473.38639	1622.801412
AM3PA	20.536 2	9367.161455	456.1292476	9367.161455	456.1292476
UM4PCP	33.567 5	9321.330906	277.6891608	9321.330906	277.6891608
CBLAT	21.998 9	9949.384616	452.2673687	9949.384616	452.2673687
TDP	17.432 9	157841.5063	9054.231155	157841.5063	9054.231155

3PEPTabcpp_num1	206.8469	26082.63514	126.0963309	26082.63514	126.0963309
3PEPTabcpp_num2	200.7513	26082.63514	129.925112	26082.63514	129.925112
3PEPTabcpp_num3	201.75	26082.63514	129.2819586	26082.63514	129.2819586
4PEPTabcpp_num1	201.75	49599.60815	245.8468805	49599.60815	245.8468805
4PEPTabcpp_num2	206.8469	49599.60815	239.7889847	49599.60815	239.7889847
AGM3Pt2pp	53.2452	14196.18975	266.619146	14196.18975	266.619146
AGM4Pt2pp	53.2452	37678.09115	707.6335735	37678.09115	707.6335735
AGMt2pp	53.2452	34185.31625	642.0356436	34185.31625	642.0356436
FEOXAMR1	30.1128	26079.92347	866.0743429	26079.92347	866.0743429
FEOXAMR2	30.1128	10115.68801	335.9265165	10115.68801	335.9265165
FEOXAMR3	30.1128	57714.15197	1916.598655	57714.15197	1916.598655
PTHRpp_num1	104.4142	21548.48395	206.3750328	21548.48395	206.3750328
PTHRpp_num2	98.8772	21548.48395	217.9317775	21548.48395	217.9317775
ARABDI	389.86	86322.63719	221.4195793	86322.63719	221.4195793
R5PPpp_num1	104.4142	23570.47054	225.7400865	23570.47054	225.7400865
R5PPpp_num2	98.8772	23570.47054	238.3812501	23570.47054	238.3812501
PSP_Lpp_num1	104.4142	23226.39009	222.444745	23226.39009	222.444745
PSP_Lpp_num2	98.8772	23226.39009	234.9013736	23226.39009	234.9013736
TYRPpp	104.4142	9537.079284	91.33891065	9537.079284	91.33891065
PAPSR2_num1	80.3019	25460.24801	317.0566078	25460.24801	317.0566078
PAPSR2_num2	65.0891	25460.24801	391.1599333	25460.24801	391.1599333
PAPSR2_num3	65.6365	25460.24801	387.8977096	25460.24801	387.8977096

PAPSR2_num4	81.7092	25460.24801	311.5958547	25460.24801	311.5958547
GRXR_num1	25.7575	11950.26729	463.9529181	11950.26729	463.9529181
GRXR_num2	24.3502	11950.26729	490.7666996	11950.26729	490.7666996
GRXR_num3	9.1375	11950.26729	1307.826789	11950.26729	1307.826789
GRXR_num4	9.6849	11950.26729	1233.907143	11950.26729	1233.907143
RNDR1b_num1	243.5294	22837.66644	93.7778619	22837.66644	93.7778619
RNDR1b_num2	258.1947	22837.66644	88.45133708	22837.66644	88.45133708
RNDR1b_num3	242.982	22837.66644	93.98912859	22837.66644	93.98912859
RNDR1b_num4	259.602	22837.66644	87.97184322	22837.66644	87.97184322
RNDR2b_num1	259.602	27735.00971	106.8366565	27735.00971	106.8366565
RNDR2b_num2	243.5294	27735.00971	113.8877265	27735.00971	113.8877265
RNDR2b_num3	258.1947	27735.00971	107.4189738	27735.00971	107.4189738
RNDR2b_num4	242.982	27735.00971	114.1442975	27735.00971	114.1442975
FACOAE181	127.8653	28297.73827	221.3089733	28297.73827	221.3089733
FACOAE160	127.8653	129600	1013.56662	129600	1013.56662
FACOAE140	127.8653	63347.75856	495.4257219	63347.75856	495.4257219
AAMYL	56.6394	23490.41652	414.7363235	23490.41652	414.7363235
AAMYLpp	75.7127	49598.11858	655.0832103	49598.11858	655.0832103
FACOAL80t2pp_num1	124.664	17459.32014	140.0510183	17459.32014	140.0510183
FACOAL80t2pp_num2	60.7731	17459.32014	287.2869763	17459.32014	287.2869763
PPGPPDP	79.3424	90720.84686	1143.409411	90720.84686	1143.409411
23PDE2pp	70.8322	52628.11949	742.9971043	52628.11949	742.9971043
23PDE4pp	70.832	48310.69378	682.0442366	48310.69378	682.0442366

	2				
23PDE9pp	70.832<br>2	43543.38935	614.7400384	43543.38935	614.7400384
SARCOX	40.902	18240.21953	445.9493307	18240.21953	445.9493307
MN6PP_num1	29.721<br>1	11553.01474	388.7142381	11553.01474	388.7142381
MN6PP_num2	30.412<br>8	11553.01474	379.873433	11553.01474	379.873433
MN6PP_num3	24.330<br>2	11553.01474	474.8425719	11553.01474	474.8425719
MN6PP_num4	29.707<br>7	11553.01474	388.8895722	11553.01474	388.8895722
3KGK	110.30<br>97	46839.28669	424.6162096	46839.28669	424.6162096
F6PP_num1	23.007<br>5	18699.03043	812.7363003	18699.03043	812.7363003
F6PP_num2	29.721<br>1	18699.03043	629.1500122	18699.03043	629.1500122
F6PP_num3	30.412<br>8	18699.03043	614.8408048	18699.03043	614.8408048
F6PP_num4	20.779<br>8	18699.03043	899.8657556	18699.03043	899.8657556
F6PP_num5	29.707<br>7	18699.03043	629.4337976	18699.03043	629.4337976
GDPMNH	36.547	111348.1079	3046.709932	111348.1079	3046.709932
FACOAL60t2pp_<br>num1	124.66<br>4	24310.91002	195.011471	24310.91002	195.011471
FACOAL60t2pp_<br>num2	60.773<br>1	24310.91002	400.0274796	24310.91002	400.0274796
GDPDPK_num1	83.875<br>6	40330.37894	480.8356536	40330.37894	480.8356536
GDPDPK_num2	79.342<br>4	40330.37894	508.3080288	40330.37894	508.3080288
R15BPK	20.729<br>6	17862.19588	861.6758585	17862.19588	861.6758585
GGPTRCS	638.12<br>59	10428.39012	16.34221415	10428.39012	16.34221415
FRULYSDG	462.83<br>08	41650.55762	89.99089435	41650.55762	89.99089435
ASNNpp	147.40<br>28	56027.20568	380.095939	56027.20568	380.095939
GLUNpp	147.40<br>28	20661.07493	140.1674523	20661.07493	140.1674523

NTD12_num1	25.3988	9521.232462	374.8693821	9521.232462	374.8693821
NTD12_num2	26.9004	9521.232462	353.943899	9521.232462	353.943899
NTD12_num3	45.4161	9521.232462	209.6444314	9521.232462	209.6444314
NTD12_num4	20.2554	9521.232462	470.0589701	9521.232462	470.0589701
DNTPPA_num1	17.3058	9493.606192	548.5794469	9493.606192	548.5794469
DNTPPA_num2	14.9271	9493.606192	635.9980299	9493.606192	635.9980299
NTPP9	42.0777	219466.3262	5215.739601	219466.3262	5215.739601
NTPP10	42.0777	25719.40426	611.2359815	25719.40426	611.2359815
NTPP11	42.0777	9764.884213	232.0679175	9764.884213	232.0679175
NTD10pp_num1	104.4142	16669.85067	159.6511841	16669.85067	159.6511841
NTD10pp_num2	60.824	16669.85067	274.0669912	16669.85067	274.0669912
NTD1pp_num1	60.824	18062.0881	296.9565977	18062.0881	296.9565977
NTD1pp_num2	104.4142	18062.0881	172.9849781	18062.0881	172.9849781
NTD2pp_num1	60.824	28742.74164	472.555926	28742.74164	472.555926
NTD2pp_num2	104.4142	28742.74164	275.2761755	28742.74164	275.2761755
NTD2pp_num3	98.8772	28742.74164	290.6912983	28742.74164	290.6912983
NTD3pp_num1	60.824	17056.30725	280.4206769	17056.30725	280.4206769
NTD3pp_num2	104.4142	17056.30725	163.3523721	17056.30725	163.3523721
NTD4pp_num1	104.4142	16503.42699	158.0573044	16503.42699	158.0573044
NTD4pp_num2	60.824	16503.42699	271.3308397	16503.42699	271.3308397
NTD4pp_num3	98.8772	16503.42699	166.9083165	16503.42699	166.9083165
NTD5pp_num1	104.4142	18932.08788	181.3171761	18932.08788	181.3171761
NTD5pp_num2	60.824	18932.08788	311.2601585	18932.08788	311.2601585
NTD6pp_num1	60.824	18118.8497	297.8898083	18118.8497	297.8898083
NTD6pp_num2	104.4142	18118.8497	173.5285976	18118.8497	173.5285976

NTD7pp_num1	104.4142	16283.31909	155.9492779	16283.31909	155.9492779
NTD7pp_num2	60.824	16283.31909	267.7120723	16283.31909	267.7120723
NTD7pp_num3	98.8772	16283.31909	164.6822431	16283.31909	164.6822431
NTD9pp_num1	104.4142	17969.26403	172.0959796	17969.26403	172.0959796
NTD9pp_num2	60.824	17969.26403	295.4304885	17969.26403	295.4304885
NTD9pp_num3	98.8772	17969.26403	181.73314	17969.26403	181.73314
PEAMNOpp	168.7573	25099.97591	148.7341639	25099.97591	148.7341639
GAL1PPpp	91.3658	49931.64198	546.5025423	49931.64198	546.5025423
ARGDCpp	295.5938	11541.49804	39.04512896	11541.49804	39.04512896
ALLPI	32.1466	19201.33747	597.3053906	19201.33747	597.3053906
ALLK	33.8209	23208.86213	686.2284012	23208.86213	686.2284012
ALLULPE	26.1092	28397.97262	1087.661538	28397.97262	1087.661538
FACOAL100t2pp_num1	124.664	19405.05356	155.6588395	19405.05356	155.6588395
FACOAL100t2pp_num2	60.7731	19405.05356	319.3033359	19405.05356	319.3033359
GPDDA3pp	81.6867	36706.06811	449.3518297	36706.06811	449.3518297
GPDDA4pp	81.6867	41144.26043	503.6837138	41144.26043	503.6837138
GPDDA2pp	81.6867	41162.26921	503.9041755	41162.26921	503.9041755
FACOAL120t2pp_num1	60.7731	19622.55997	322.8823274	19622.55997	322.8823274
FACOAL120t2pp_num2	124.664	19622.55997	157.4035806	19622.55997	157.4035806
FACOAL140t2pp_num1	60.7731	15392.79644	253.2830551	15392.79644	253.2830551
FACOAL140t2pp_num2	124.664	15392.79644	123.4742703	15392.79644	123.4742703
HCYSMT2	33.4228	9603.528682	287.3346542	9603.528682	287.3346542
ACGK	54.319	30240	556.7102548	30240	556.7102548

	1				
DHPTPE	112.6563	17759.95224	157.6472176	17759.95224	157.6472176
NADPHQR2	43.7818	27721.05118	633.1638074	27721.05118	633.1638074
NADPHQR3	43.7818	30528.29321	697.2827341	30528.29321	697.2827341
NADPHQR4	43.7818	16809.95379	383.9484394	16809.95379	383.9484394
GLBRAN2	84.3369	221256.5004	2623.483913	221256.5004	2623.483913
ASR	40.2035	16666.55749	414.5548894	16666.55749	414.5548894
ECAtpp	44.9597	86843.56536	1931.586851	86843.56536	1931.586851
O16AT	21.6751	23247.17566	1072.529108	23247.17566	1072.529108
O16GALFT	37.7572	22063.75587	584.3589003	22063.75587	584.3589003
O16AUNDtpp	45.3826	56567.01265	1246.447155	56567.01265	1246.447155
O16AP1pp	81.199	18394.40465	226.5348668	18394.40465	226.5348668
AGM4PCP	33.5675	46441.37344	1383.521961	46441.37344	1383.521961
4PCP	33.5675	27706.1783	825.3870052	27706.1783	825.3870052
4PCPpp	21.0399	37289.77556	1772.33616	37289.77556	1772.33616
FALDH2	78.718	29317.27206	372.4341581	29317.27206	372.4341581
CBIAT	21.9989	17689.46963	804.1070066	17689.46963	804.1070066
PPDOy	37.9784	27609.90566	726.989701	27609.90566	726.989701
NTD12pp_num1	60.824	17007.54339	279.6189561	17007.54339	279.6189561
NTD12pp_num2	104.4142	17007.54339	162.8853488	17007.54339	162.8853488
PPA2_num1	116.2719	9737.917049	83.75125072	9737.917049	83.75125072
PPA2_num2	26.9004	9737.917049	361.9989684	9737.917049	361.9989684
3NTD7pp_num1	70.8322	55966.42332	790.1268536	55966.42332	790.1268536
3NTD7pp_num2	98.877	55966.42332	566.0195001	55966.42332	566.0195001

	2				
3NTD4pp_num1	98.877 2	46917.55912	474.5033144	46917.55912	474.5033144
3NTD4pp_num2	70.832 2	46917.55912	662.3761385	46917.55912	662.3761385
3NTD4pp_num3	98.877 2	46917.55912	474.5033144	46917.55912	474.5033144
3NTD2pp_num1	70.832 2	44836.48256	632.9957641	44836.48256	632.9957641
3NTD2pp_num2	98.877 2	44836.48256	453.4562322	44836.48256	453.4562322
3NTD9pp_num1	70.832 2	39469.58003	557.226516	39469.58003	557.226516
3NTD9pp_num2	98.877 2	39469.58003	399.1777683	39469.58003	399.1777683
23PDE7pp	70.832 2	57434.65448	810.8551546	57434.65448	810.8551546
HSTPT	78.720 3	11880	150.914059	11880	150.914059
THDPS	59.784 2	8640	144.5197895	8640	144.5197895
ACONMT	29.006	176845.203	6096.849032	176845.203	6096.849032
GNK_num1	39.086 6	26754.97352	684.50501	26754.97352	684.50501
GNK_num2	21.004	26754.97352	1273.803729	26754.97352	1273.803729
HMBS	33.851 8	468	13.82496647	468	13.82496647
RMK	54.069	33912.96639	627.216453	33912.96639	627.216453
FHL_num1	467.42 48	166094.0433	355.3385343	166094.0433	355.3385343
FHL_num2	311.49 24	166094.0433	533.2202113	166094.0433	533.2202113
MEPCT	51.474 5	12960	251.7751508	12960	251.7751508
GLUDy	291.48 82	180117.9189	617.9252503	180117.9189	617.9252503
GLYCLTDy_num 1	35.343 4	62636.42448	1772.224078	62636.42448	1772.224078
GLYCLTDy_num 2	70.791	62636.42448	884.8077366	62636.42448	884.8077366
LYSDC_num1	805.90 3	11370.9205	14.10953986	11370.9205	14.10953986
LYSDC_num2	812.60	11370.9205	13.99323617	11370.9205	13.99323617

	12				
DAAD	95.2146	27473.02376	288.5379318	27473.02376	288.5379318
NTPP5_num1	17.3058	21294.56777	1230.487338	21294.56777	1230.487338
NTPP5_num2	60.8246	21294.56777	350.09795	21294.56777	350.09795
NTD3_num1	45.4161	11259.28806	247.9140229	11259.28806	247.9140229
NTD3_num2	26.9004	11259.28806	418.5546705	11259.28806	418.5546705
NTD3_num3	20.2554	11259.28806	555.8659941	11259.28806	555.8659941
GLYOX	28.4344	10623.00336	373.5968884	10623.00336	373.5968884
MTRPOX	40.902	11031.88203	269.714978	11031.88203	269.714978
AGM4PCPpp	21.0399	34105.01658	1620.968568	34105.01658	1620.968568
GTPDPDP	109.7423	11616.65916	105.8539793	11616.65916	105.8539793
RNDR2_num1	270.3917	10997.84578	40.67375506	10997.84578	40.67375506
RNDR2_num2	274.1399	10997.84578	40.11763985	10997.84578	40.11763985
XAND	129.7994	65517.63512	504.7606932	65517.63512	504.7606932
HACD3_num1	159.1878	39593.4512	248.7216433	39593.4512	248.7216433
HACD3_num2	77.0722	39593.4512	513.7189701	39593.4512	513.7189701
NNDMBRT	73.974	31557.09164	426.5970697	31557.09164	426.5970697
THRA2_num1	90.6332	17869.47753	197.1626018	17869.47753	197.1626018
THRA2_num2	145.9788	17869.47753	122.4114565	17869.47753	122.4114565
PPK	160.863	29958.53743	186.2363466	29958.53743	186.2363466
ABUTD	203.32	38704.04507	190.3602453	38704.04507	190.3602453
E4PD_num1	149.1976	9322.452155	62.48392839	9322.452155	62.48392839
E4PD_num2	142.13	9322.452155	65.59102339	9322.452155	65.59102339
IG3PS	50.1073	32266.5357	643.9487999	32266.5357	643.9487999

DADK	23.586	15176.86818	643.4693541	15176.86818	643.4693541
DUTPDP_num1	48.4664	13211.96805	272.6005655	13211.96805	272.6005655
DUTPDP_num2	16.3707	13211.96805	807.0496708	13211.96805	807.0496708
NTP5	39.1933	30671.51304	782.5703128	30671.51304	782.5703128
MTAN	48.7079	9431.398224	193.6317974	9431.398224	193.6317974
DPR_num1	33.8708	9584.979242	282.9865029	9584.979242	282.9865029
DPR_num2	216.2761	9584.979242	44.3182545	9584.979242	44.3182545
THRD_L_num1	224.781	57035.45056	253.7378629	57035.45056	253.7378629
THRD_L_num2	140.9296	57035.45056	404.7088089	57035.45056	404.7088089
NAMNPP	45.8973	9308.69978	202.8158471	9308.69978	202.8158471
SELNPS	73.3745	91807.86884	1251.223093	91807.86884	1251.223093
CRNCAR	28.1902	203054.0799	7203.002458	203054.0799	7203.002458
ANHMK	39.4963	21852.99274	553.29215	21852.99274	553.29215
DHFR_num1	26.3483	17578.90389	667.1741209	17578.90389	667.1741209
DHFR_num2	17.9994	17578.90389	976.6383262	17578.90389	976.6383262
PRAIi	49.4923	18000	363.6929381	18000	363.6929381
TYRTA_num1	87.1467	69547.95976	798.0561485	69547.95976	798.0561485
TYRTA_num2	87.0756	69547.95976	798.7077868	69547.95976	798.7077868
TYRTA_num3	204.5619	69547.95976	339.9849129	69547.95976	339.9849129
SHKK_num1	19.538	61262.15929	3135.538914	61262.15929	3135.538914
SHKK_num2	19.1508	61262.15929	3198.934733	61262.15929	3198.934733
SADH	98.5971	62717.07204	636.09449	62717.07204	636.09449
GLUSy	861.24	135501.647	157.331558	135501.647	157.331558

	9				
GUAPRT_num1	80.461	17179.19996	213.5096501	17179.19996	213.5096501
GUAPRT_num2	67.8824	17179.19996	253.0729609	17179.19996	253.0729609
PDH	4586.6113	270261.5203	58.92400786	3596400	784.1083024
DMSOR2pp	129.2504	79462.90645	614.7981473	79462.90645	614.7981473
HYD3pp_num1	482.9335	29005.97007	60.06203767	29005.97007	60.06203767
HYD3pp_num2	181.7482	29005.97007	159.5942632	29005.97007	159.5942632
NO3R1pp_num1	250.963	401469.1895	1599.714657	401469.1895	1599.714657
NO3R1pp_num2	474.5552	401469.1895	845.990497	401469.1895	845.990497
AACPS5	89.3395	9300.08354	104.0982269	9300.08354	104.0982269
NTD8_num1	25.3988	9939.384184	391.3328261	9939.384184	391.3328261
NTD8_num2	26.9004	9939.384184	369.4883416	9939.384184	369.4883416
NTD8_num3	45.4161	9939.384184	218.8515567	9939.384184	218.8515567
NTD8_num4	20.2554	9939.384184	490.7029327	9939.384184	490.7029327
GALCTD	56.4016	72941.9405	1293.260129	72941.9405	1293.260129
MLTG4	69.041	40858.37928	591.7987758	40858.37928	591.7987758
UDPGDC	445.7333	10429.01417	23.39743108	10429.01417	23.39743108
PUNP4_num1	179.0077	15875.68535	88.68716457	15875.68535	88.68716457
PUNP4_num2	155.6995	15875.68535	101.9636245	15875.68535	101.9636245
AMAOTr	94.6712	3.6	0.038026348	3.6	0.038026348
NTD11pp_num1	60.824	16148.02293	265.4876846	16148.02293	265.4876846
NTD11pp_num2	104.4142	16148.02293	154.6535139	16148.02293	154.6535139
DKGLCNR2x	70.791	19157.06075	270.6143542	19157.06075	270.6143542
ACGS	295.1711	820800	2780.760041	820800	2780.760041

PDE1_num1	180.5208	123619.6494	684.7944915	123619.6494	684.7944915
PDE1_num2	30.938	123619.6494	3995.722071	123619.6494	3995.722071
MCITD	53.9518	37457.91464	694.2847995	37457.91464	694.2847995
GLCATr	60.288	10408.43895	172.6452851	10408.43895	172.6452851
ALAALAr_num1	39.3158	17736.16695	451.1205916	17736.16695	451.1205916
ALAALAr_num2	65.6794	17736.16695	270.0415496	17736.16695	270.0415496
ACACT1r_num1	46.5305	64745.49694	1391.463598	64745.49694	1391.463598
ACACT1r_num2	161.4099	64745.49694	401.1246952	64745.49694	401.1246952
ACACT1r_num3	81.7524	64745.49694	791.9705958	64745.49694	791.9705958
G2PPpp_num1	104.4142	26350.74038	252.3674019	26350.74038	252.3674019
G2PPpp_num2	98.8772	26350.74038	266.499662	26350.74038	266.499662
AGM3PApp_num1	45.6344	23614.90817	517.4804132	23614.90817	517.4804132
AGM3PApp_num2	47.9852	23614.90817	492.1289932	23614.90817	492.1289932
AGM3PApp_num3	31.4121	23614.90817	751.7774414	23614.90817	751.7774414
AGM3PApp_num4	31.0724	23614.90817	759.9962722	23614.90817	759.9962722
SUCOAS	142.3403	28613.05984	201.0186844	28613.05984	201.0186844
NTP1_num1	61.9863	27027.32662	436.0209694	27027.32662	436.0209694
NTP1_num2	39.1933	27027.32662	689.5904815	27027.32662	689.5904815
GALKr	41.4421	44343.2048	1070.003808	44343.2048	1070.003808
PANTS	63.1953	72	1.139325235	72	1.139325235
YUMPS	98.7297	17972.16522	182.0340305	17972.16522	182.0340305
DHQS	38.881	280800	7222.03647	280800	7222.03647
PGCD	176.7031	72000	407.4631401	72000	407.4631401

ADSL1r	206.1712	36000	174.612167	36000	174.612167
SERD_D	47.9006	27507.13332	574.2544629	27507.13332	574.2544629
AICART	57.3292	36000	627.9522477	36000	627.9522477
PFK_num1	139.3682	1000800	7180.978157	1000800	7180.978157
PFK_num2	64.912	1000800	15417.7964	1000800	15417.7964
PGM_num1	57.1128	164087.7094	2873.046138	164087.7094	2873.046138
PGM_num2	56.1939	164087.7094	2920.027075	164087.7094	2920.027075
TPI	53.9436	259200	4805.018575	259200	4805.018575
RPI_num1	45.7208	71002.38272	1552.955826	71002.38272	1552.955826
RPI_num2	32.1466	71002.38272	2208.705827	71002.38272	2208.705827
PERD	82.7353	72	0.870245228	72	0.870245228
PYDAMK	61.6948	9300.695926	150.75332	9300.695926	150.75332
FADRx2	530.1587	51368.76695	96.89318868	51368.76695	96.89318868
PDE4	180.5208	64875.80671	359.3813384	64875.80671	359.3813384
AGM4PH	37.5947	9954.435204	264.7829403	9954.435204	264.7829403
AM4PA	20.5362	20712.18859	1008.569677	20712.18859	1008.569677
ATHRDHr	108.9957	13509.71589	123.9472373	13509.71589	123.9472373
LSERDHr	108.9957	14125.37111	129.5956732	14125.37111	129.5956732
DSERDHr	108.9957	15835.52183	145.2857482	15835.52183	145.2857482
OBTFL_num1	198.9187	21920.42484	110.1979092	21920.42484	110.1979092
OBTFL_num2	213.2029	21920.42484	102.8148531	21920.42484	102.8148531
OBTFL_num3	114.1398	21920.42484	192.0489158	21920.42484	192.0489158

G6PP_num1	29.7077	9870.743437	332.2621218	9870.743437	332.2621218
G6PP_num2	30.4128	9870.743437	324.5588514	9870.743437	324.5588514
G6PP_num3	23.0075	9870.743437	429.0228594	9870.743437	429.0228594
MMCD	175.0355	22988.03684	131.3335685	22988.03684	131.3335685
GLNS	622.8451	119723.2776	192.2199879	3596400	5774.14834
GTHS	142.2436	42649.46597	299.8339888	42649.46597	299.8339888
UDPKAAT	41.6498	10842.16108	260.3172422	10842.16108	260.3172422
ULA4NFT	445.7333	14287.53528	32.05400019	14287.53528	32.05400019
UPLA4FNT	36.3391	32384.40508	891.1724583	32384.40508	891.1724583
UPLA4FNF	33.1118	772257.6065	23322.73107	772257.6065	23322.73107
ULA4Ntppi	26.2771	36865.93205	1402.968061	36865.93205	1402.968061
HEPT1	35.5438	10345.03235	291.0502633	10345.03235	291.0502633
ALR2_num1	30.9866	10013.59023	323.1587277	10013.59023	323.1587277
ALR2_num2	31.1096	10013.59023	321.8810345	10013.59023	321.8810345
ALR2_num3	29.4368	10013.59023	340.172513	10013.59023	340.172513
ALR2_num4	38.8322	10013.59023	257.8682184	10013.59023	257.8682184
AMALT2	78.5035	43134.18746	549.4555971	43134.18746	549.4555971
MLTP1	181.0448	24684.35694	136.3439156	24684.35694	136.3439156
HEPT2	39.0421	10836.90288	277.5696717	10836.90288	277.5696717
HXAND	129.7994	64608.61918	497.7574563	64608.61918	497.7574563
MOAT3C	32.9201	11125.6214	337.9583112	11125.6214	337.9583112
RHAT1	36.7305	34002.42145	925.72716	34002.42145	925.72716

GALT1	40.8259	95078.50661	2328.877174	95078.50661	2328.877174
GLCTR2	39.4232	15230.70238	386.3385615	15230.70238	386.3385615
UDPGPpp	60.824	13977.35334	229.7999695	13977.35334	229.7999695
PPPGO	84.9052	9913.181558	116.7558825	9913.181558	116.7558825
ADNK1	23.586	28.8	1.221063343	28.8	1.221063343
HEPK1	30.8723	20974.87796	679.4076878	20974.87796	679.4076878
HEPT3	38.7307	22408.29661	578.5667859	22408.29661	578.5667859
HEPK2	27.461	26010.86203	947.19282	26010.86203	947.19282
ALDD3y_num1	36.5018	23752.9732	650.734298	23752.9732	650.734298
ALDD3y_num2	225.2254	23752.9732	105.4631192	23752.9732	105.4631192
ALDD3y_num3	84.194	23752.9732	282.1219232	23752.9732	282.1219232
R5PP_num1	23.0075	9881.341639	429.4835006	9881.341639	429.4835006
R5PP_num2	20.7798	9881.341639	475.5263111	9881.341639	475.5263111
R5PP_num3	30.4128	9881.341639	324.9073298	9881.341639	324.9073298
R5PP_num4	27.1631	9881.341639	363.7781269	9881.341639	363.7781269
R5PP_num5	29.7211	9881.341639	332.4689072	9881.341639	332.4689072
CYSSADS_num1	86.4684	13159.47439	152.188249	13159.47439	152.188249
CYSSADS_num2	88.8675	13159.47439	148.0797186	13159.47439	148.0797186
CPPPGO2	52.729	9383.577178	177.9585651	9383.577178	177.9585651
AGM3PH	37.5947	9954.457142	264.7835238	9954.457142	264.7835238
LDH_D2	64.6123	11899.87868	184.1735812	11899.87868	184.1735812
SHCHF	99.9028	1224	12.25190886	1224	12.25190886
SUCBZL	200.7387	16127.31824	80.33985596	16127.31824	80.33985596
CYSDDS	70.3066	27651.34377	393.2965578	27651.34377	393.2965578

NTP10_num1	39.1933	10200.0983	260.251071	10200.0983	260.251071
NTP10_num2	36.4253	10200.0983	280.027846	10200.0983	280.027846
PRAIS	73.7081	36000	488.4130781	36000	488.4130781
GDMANE	72.2823	130011.786	1798.666976	130011.786	1798.666976
GPDDA4	27.4096	9300.271378	339.3070814	9300.271378	339.3070814
LPLIPAL2E140	38.9783	9300.115008	238.5972453	9300.115008	238.5972453
LPLIPAL2E180	38.9783	21392.64662	548.8347778	21392.64662	548.8347778
LPLIPAL2G161	38.9783	11808.07575	302.939732	11808.07575	302.939732
LPLIPAL2G181	38.9783	10096.07048	259.0177221	10096.07048	259.0177221
AACPS6	89.3395	16920.24286	189.3926299	16920.24286	189.3926299
AACPS7	89.3395	19220.43512	215.1392734	19220.43512	215.1392734
QMO2	23.0648	24600.84318	1066.596857	24600.84318	1066.596857
QMO3	23.0648	33447.80076	1450.166521	33447.80076	1450.166521
LPLIPAL2E141	38.9783	9300.051788	238.5956234	9300.051788	238.5956234
LPLIPAL2E161	38.9783	10543.91586	270.5073299	10543.91586	270.5073299
LPLIPAL2E181	38.9783	9300.052674	238.5956461	9300.052674	238.5956461
LPLIPAL2G180	38.9783	10096.05001	259.017197	10096.05001	259.017197
LPLIPAL2G141	38.9783	9300.047494	238.5955132	9300.047494	238.5955132
IPDDI	20.5083	16631.67284	810.972769	16631.67284	810.972769
2AGPGAT180	80.7	9737.355798	120.6611623	9737.355798	120.6611623
2AGPGAT181	80.7	25994.16807	322.1086502	25994.16807	322.1086502
LPLIPAL2ATG120	38.9783	15263.39692	391.5870349	15263.39692	391.5870349
PLIPA1E140pp	66.326	9300.046259	140.2165696	9300.046259	140.2165696

	3				
PLIPA1E160pp	66.326 3	9319.15782	140.5047141	9319.15782	140.5047141
PLIPA1E161pp	66.326 3	12158.28134	183.3101099	12158.28134	183.3101099
AACPS8	89.339 5	19300.13799	216.0314082	19300.13799	216.0314082
AACPS9	89.339 5	9300.081582	104.098205	9300.081582	104.098205
HXCT	92.971 7	30493.60812	327.9880665	30493.60812	327.9880665
2AGPEAT160	80.7	83842.72261	1038.943279	83842.72261	1038.943279
DURADx	178.79 66	38271.10824	214.0482998	38271.10824	214.0482998
ECOAH7_num1	77.072 2	37020.63211	480.3370361	37020.63211	480.3370361
ECOAH7_num2	159.18 78	37020.63211	232.5594808	37020.63211	232.5594808
G1PPpp_num1	91.365 8	34600.56248	378.7036558	34600.56248	378.7036558
G1PPpp_num2	98.877 2	34600.56248	349.9346915	34600.56248	349.9346915
GALCTND	42.522 9	20418.46137	480.1756552	20418.46137	480.1756552
GCALDD	209.09 12	72	0.344347347	72	0.344347347
HISTP	80.555 9	13680	169.8199635	13680	169.8199635
LDH_D	146.13 92	41919.84126	286.8487118	41919.84126	286.8487118
AMALT4	78.503 5	32028.92891	407.9936425	32028.92891	407.9936425
NTD11_num1	26.900 4	9325.752358	346.6770887	9325.752358	346.6770887
NTD11_num2	25.398 8	9325.752358	367.1729514	9325.752358	367.1729514
ORNDC_num1	158.83 24	35745.33522	225.0506523	35745.33522	225.0506523
ORNDC_num2	164.83 16	35745.33522	216.8597236	35745.33522	216.8597236
UPP3MT	99.902 8	9455.38735	94.64586928	9455.38735	94.64586928
KAS15	67.030	22049.2422	328.9448965	22049.2422	328.9448965

	2				
FACOAL181t2pp _num1	124.66 4	20613.48472	165.3523449	20613.48472	165.3523449
FACOAL181t2pp _num2	60.773 1	20613.48472	339.1876459	20613.48472	339.1876459
T2DECAI	37.938 1	9300.050359	245.1374834	9300.050359	245.1374834
3OAS161	85.226 6	9300.049776	109.1214454	9300.049776	109.1214454
EAR141y	111.45 58	9385.00781	84.2038531	9385.00781	84.2038531
EAR161x	111.45 58	9300.026639	83.44138788	9300.026639	83.44138788
DASYN160	31.453 8	237600	7553.936249	237600	7553.936249
DASYN161	31.453 8	280800	8927.379204	280800	8927.379204
DASYN181	31.453 8	9600.275596	305.2183073	9600.275596	305.2183073
CDAPPA161	28.450 8	10309.94605	362.3780721	10309.94605	362.3780721
PGSA180	20.700 6	21211.67154	1024.688731	21211.67154	1024.688731
PSSA140	52.801 8	18624.88315	352.7319742	18624.88315	352.7319742
2AGPGAT141	80.7	9645.640135	119.5246609	9645.640135	119.5246609
FACOAE180	127.86 53	44832.33864	350.6216201	44832.33864	350.6216201
FACOAE100	127.86 53	148841.6558	1164.050417	148841.6558	1164.050417
FACOAE80	127.86 53	9300.085783	72.73346078	9300.085783	72.73346078
FACOAE60	127.86 53	10237.32554	80.06335993	10237.32554	80.06335993
PLIPA1E120pp	66.326 3	9300.055576	140.21671	9300.055576	140.21671
3OAS141	85.226 6	9300.05716	109.121532	9300.05716	109.121532
3OAR121	102.24 12	9300.02693	90.96163709	9300.02693	90.96163709
CFAS160G	87.818	114927.3368	1308.699092	114927.3368	1308.699092
FACOAE141	127.86 53	22065.15168	172.5655958	22065.15168	172.5655958

FACOAE161	127.8653	154800	1210.649019	154800	1210.649019
ALAALAD	21.213	21385.38081	1008.126187	21385.38081	1008.126187
2AGPEAT180	80.7	22628.36988	280.4011138	22628.36988	280.4011138
2AGPEAT181	80.7	21320.30109	264.1920829	21320.30109	264.1920829
2AGPGAT140	80.7	26004.84427	322.2409451	26004.84427	322.2409451
PLIPA1E181pp	66.3263	9300.044405	140.2165416	9300.044405	140.2165416
PLIPA1G180pp	66.3263	10731.71028	161.8017329	10731.71028	161.8017329
PLIPA1G181pp	66.3263	9331.151328	140.6855399	9331.151328	140.6855399
2AGPGAT161	80.7	9645.635293	119.5246009	9645.635293	119.5246009
AMPTASEPG_num1	329.2789	29612.1696	89.93035873	29612.1696	89.93035873
AMPTASEPG_num2	277.081	29612.1696	106.871888	29612.1696	106.871888
AMPTASEPG_num3	98.9189	29612.1696	299.3580559	29612.1696	299.3580559
AMPTASEPG_num4	105.8308	29612.1696	279.8067254	29612.1696	279.8067254
2AGPEAT140	80.7	33704.87601	417.6564561	33704.87601	417.6564561
2AGPEAT120	80.7	17213.08318	213.2971894	17213.08318	213.2971894
2AGPEAT161	80.7	16922.37939	209.6949119	16922.37939	209.6949119
2AGPEAT141	80.7	18684.28389	231.5276814	18684.28389	231.5276814
2AGPGAT160	80.7	9649.160262	119.5682808	9649.160262	119.5682808
LPLIPAL2ATG140	38.9783	14782.19516	379.2416591	14782.19516	379.2416591
LPLIPAL2ATG141	38.9783	9726.546006	249.5374607	9726.546006	249.5374607
LPLIPAL2ATG160	38.9783	41172.89897	1056.303096	41172.89897	1056.303096
LPLIPAL2ATG180	38.9783	14302.36908	366.9315767	14302.36908	366.9315767
LPLIPAL2ATG161	38.9783	28569.38076	732.9560489	28569.38076	732.9560489
LPLIPAL2ATG181	38.9783	19600.74689	502.8630518	19600.74689	502.8630518
CLPNS180pp_num1	54.8223	18171.28853	331.4579748	18171.28853	331.4579748
CLPNS180pp_num2	47.6335	18171.28853	381.4812795	18171.28853	381.4812795
CLPNS181pp_nu	54.822	21182.30828	386.3812405	21182.30828	386.3812405

m1	3				
CLPNS181pp_num2	47.6335	21182.30828	444.6935094	21182.30828	444.6935094
PLIPA1G120pp	66.3263	9300.04673	140.2165767	9300.04673	140.2165767
CLPNS120pp_num1	47.6335	27301.68336	573.1613961	27301.68336	573.1613961
CLPNS120pp_num2	54.8223	27301.68336	498.0032462	27301.68336	498.0032462
ALPATE160pp	82.0363	16171.43159	197.1253164	16171.43159	197.1253164
ALPATG160pp	82.0363	27918.59867	340.3200616	27918.59867	340.3200616
2AGPGAT120	80.7	17444.69856	216.1672684	17444.69856	216.1672684
LPLIPAL2ATE120	38.9783	15214.42477	390.3306395	15214.42477	390.3306395
LPLIPAL2ATE140	38.9783	9795.36019	251.3029093	9795.36019	251.3029093
LPLIPAL2ATE160	38.9783	27150.84664	696.5631298	27150.84664	696.5631298
LPLIPAL2ATE180	38.9783	9726.60245	249.5389088	9726.60245	249.5389088
LPLIPAL2ATE141	38.9783	24162.76916	619.903104	24162.76916	619.903104
LPLIPAL2ATE161	38.9783	16457.64902	422.2259314	16457.64902	422.2259314
LPLIPAL2ATE181	38.9783	9769.098535	250.6291587	9769.098535	250.6291587
FACOAL141t2pp_num1	60.7731	18524.51186	304.8143317	18524.51186	304.8143317
FACOAL141t2pp_num2	124.664	18524.51186	148.5955196	18524.51186	148.5955196
FACOAL161t2pp_num1	124.664	21999.51044	176.470436	21999.51044	176.470436
FACOAL161t2pp_num2	60.7731	21999.51044	361.9942119	21999.51044	361.9942119
PGPP140pp_num1	19.4181	9509.043636	489.7000034	9509.043636	489.7000034
PGPP140pp_num2	29.0213	9509.043636	327.6573977	9509.043636	327.6573977
PGPP141pp_num1	19.4181	17501.08267	901.2767815	17501.08267	901.2767815
PGPP141pp_num	29.021	17501.08267	603.0426849	17501.08267	603.0426849

2	3				
PGPP160pp_num1	19.4181	9538.650497	491.2247077	9538.650497	491.2247077
PGPP160pp_num2	29.0213	9538.650497	328.6775746	9538.650497	328.6775746
PGPP180pp_num1	19.4181	9522.983344	490.4178753	9522.983344	490.4178753
PGPP180pp_num2	29.0213	9522.983344	328.1377245	9522.983344	328.1377245
PLIPA1E180pp	66.3263	11091.56683	167.2272813	11091.56683	167.2272813
PLIPA1G140pp	66.3263	9331.088461	140.6845921	9331.088461	140.6845921
PLIPA1G161pp	66.3263	9331.310401	140.6879383	9331.310401	140.6879383
CLPNS160pp_num1	47.6335	33484.59677	702.9631828	33484.59677	702.9631828
CLPNS160pp_num2	54.8223	33484.59677	610.7842387	33484.59677	610.7842387
CLPNS161pp_num1	54.8223	23978.23534	437.3810538	23978.23534	437.3810538
CLPNS161pp_num2	47.6335	23978.23534	503.3901633	23978.23534	503.3901633
PAPA120pp	29.0213	15536.05477	535.3328339	15536.05477	535.3328339
PAPA140pp	29.0213	9492.458216	327.0859064	9492.458216	327.0859064
PAPA141pp	29.0213	17942.63231	618.2573595	17942.63231	618.2573595
PAPA160pp	29.0213	9448.303558	325.5644495	9448.303558	325.5644495
PAPA161pp	29.0213	14822.92706	510.7602711	14822.92706	510.7602711
PAPA180pp	29.0213	16282.17018	561.0420684	16282.17018	561.0420684
HPYRI	58.7549	50196.10612	854.3305514	50196.10612	854.3305514
TPRDCOAS	31.6444	23356.11375	738.0804738	23356.11375	738.0804738
FLVRx	26.242	10178.43535	387.8681256	10178.43535	387.8681256
GGPTRCO	47.1695	59111.19011	1253.165501	59111.19011	1253.165501
HYD1pp_num1	181.74	5271939.547	29006.83224	5271939.547	29006.83224

	82				
HYD1pp_num2	482.9335	5271939.547	10916.49171	5271939.547	10916.49171
HYD1pp_num3	232.2686	5271939.547	22697.59902	5271939.547	22697.59902
ECAP3pp	135.966	54306.83617	399.4148256	54306.83617	399.4148256
FMNRx_num1	42.5067	23127.606	544.09319	23127.606	544.09319
FMNRx_num2	26.242	23127.606	881.32025	23127.606	881.32025
DHBS	118.2243	20871.0962	176.5381246	20871.0962	176.5381246
MDDCP1pp_num1	41.849	54493.1509	1302.137468	54493.1509	1302.137468
MDDCP1pp_num2	87.2178	54493.1509	624.793917	54493.1509	624.793917
MDDCP1pp_num3	88.8879	54493.1509	613.0547678	54493.1509	613.0547678
MDDCP1pp_num4	51.7983	54493.1509	1052.025856	54493.1509	1052.025856
MDDCP1pp_num5	43.3461	54493.1509	1257.163872	54493.1509	1257.163872
MDDCP1pp_num6	47.7524	54493.1509	1141.160463	54493.1509	1141.160463
GUI2	53.9872	34845.04678	645.4316353	34845.04678	645.4316353
OMBZLM	28.0732	37665.374	1341.684382	37665.374	1341.684382
GLYCK	38.7345	29775.87608	768.7171922	29775.87608	768.7171922
MLTG5	69.041	51313.46702	743.2318046	51313.46702	743.2318046
GPDDA3	27.4096	16136.67087	588.7233259	16136.67087	588.7233259
2DOXG6PP_num1	23.0075	9865.296	428.7860915	9865.296	428.7860915
2DOXG6PP_num2	29.7077	9865.296	332.078754	9865.296	332.078754
2DOXG6PP_num3	24.3302	9865.296	405.4753352	9865.296	405.4753352
2DOXG6PP_num4	30.3709	9865.296	324.8272524	9865.296	324.8272524
AGMH	37.5947	17554.43253	466.939024	17554.43253	466.939024

MPTG_num1	186.3215	18797.02545	100.8848976	18797.02545	100.8848976
MPTG_num2	128.5962	18797.02545	146.1709246	18797.02545	146.1709246
MPTG_num3	194.8907	18797.02545	96.44906324	18797.02545	96.44906324
MPTG_num4	195.5471	18797.02545	96.12530919	18797.02545	96.12530919
MPTG2_num1	194.8907	11151.79658	57.2207734	11151.79658	57.2207734
MPTG2_num2	195.5471	11151.79658	57.02869837	11151.79658	57.02869837
MPTG2_num3	128.5962	11151.79658	86.71948769	11151.79658	86.71948769
MPTG2_num4	186.3215	11151.79658	59.85244098	11151.79658	59.85244098
MCTP1App_num1	228.3702	42697.94069	186.9680925	42697.94069	186.9680925
MCTP1App_num2	229.0266	42697.94069	186.432234	42697.94069	186.432234
MCTP2App_num1	228.3702	125796.3953	550.8441789	125796.3953	550.8441789
MCTP2App_num2	229.0266	125796.3953	549.265436	125796.3953	549.265436
MCTP1Bpp_num1	36.0824	374951.4102	10391.53189	374951.4102	10391.53189
MCTP1Bpp_num2	67.8125	374951.4102	5529.237385	374951.4102	5529.237385
SFGTHi_num1	125.0371	65737.54739	525.7443382	65737.54739	525.7443382
SFGTHi_num2	31.4245	65737.54739	2091.920234	65737.54739	2091.920234
DSBCGT	51.2433	35098.21691	684.9327993	35098.21691	684.9327993
DSBGGT	54.99	176926.5936	3217.432143	176926.5936	3217.432143
DHNAOT4	33.594	28638.76395	852.4963965	28638.76395	852.4963965
AMPMS2	70.8504	25.2	0.355679008	25.2	0.355679008
THIORDXi_num1	20.4696	21554.20653	1052.986211	21554.20653	1052.986211
THIORDXi_num2	29.4406	21554.20653	732.1252466	21554.20653	732.1252466
THIORDXi_num3	33.188	21554.20653	649.4441341	21554.20653	649.4441341

	7				
PPPGO3	84.905 2	432	5.088027588	432	5.088027588
PDX5POi	51.090 3	108	2.113904205	108	2.113904205
PLIPA1A181pp	66.326 3	9331.086136	140.684557	9331.086136	140.684557
LPLIPAL2A141	38.978 3	9300.07818	238.5963005	9300.07818	238.5963005
CYTBO3_4pp	143.93 13	2052552.006	14260.63689	2052552.006	14260.63689
EAR141x	111.45 58	14822.30279	132.9881692	14822.30279	132.9881692
EAR181x	111.45 58	24255.17127	217.6214362	24255.17127	217.6214362
EAR181y	111.45 58	16968.83979	152.2472567	16968.83979	152.2472567
G3PAT141	100.02 1	9708.960668	97.06922215	9708.960668	97.06922215
G3PAT181	100.02 1	18222.06537	182.1823954	18222.06537	182.1823954
DASYN140	31.453 8	24327.98376	773.4513402	24327.98376	773.4513402
DASYN180	31.453 8	17275.5872	549.2368871	17275.5872	549.2368871
DASYN141	31.453 8	23858.22041	758.5163132	23858.22041	758.5163132
CDAPPA160	28.450 8	33511.40562	1177.872173	33511.40562	1177.872173
CDAPPA141	28.450 8	30059.94402	1056.558832	30059.94402	1056.558832
PGSA141	20.700 6	18750.23108	905.7820101	18750.23108	905.7820101
5DOAN	48.707 9	36	0.739099817	36	0.739099817
FACOAE120	127.86 53	79200	619.4018236	79200	619.4018236
3OAR120	102.24 12	9300.029854	90.96166568	9300.029854	90.96166568
3HAD80_num1	37.938 1	9300.030919	245.136971	9300.030919	245.136971
3HAD80_num2	102.19 77	9300.030919	91.00039354	9300.030919	91.00039354

3HAD120_num1	102.1977	9300.045229	91.00053357	9300.045229	91.00053357
3HAD120_num2	37.9381	9300.045229	245.1373482	9300.045229	245.1373482
EAR180x	111.4558	12882.05534	115.5799459	12882.05534	115.5799459
EAR180y	111.4558	9300.032341	83.44143904	9300.032341	83.44143904
AGPAT120	27.4532	9300.241951	338.7671365	9300.241951	338.7671365
PGPP120_num1	19.4181	9300.055518	478.9374613	9300.055518	478.9374613
PGPP120_num2	29.0213	9300.055518	320.4562	9300.055518	320.4562
DAGK120	39.734	18231.9674	458.8505411	18231.9674	458.8505411
PSSA180	52.8018	22589.43123	427.8155524	22589.43123	427.8155524
PSSA161	52.8018	64800	1227.23089	64800	1227.23089
PSSA141	52.8018	10725.55609	203.1286072	10725.55609	203.1286072
PSD160	35.9344	24120	671.2231177	24120	671.2231177
PSD161	35.9344	28440	791.4421835	28440	791.4421835
PSD180	35.9344	11039.19848	307.2041965	11039.19848	307.2041965
COLIPAabcpp	128.9214	9847.991952	76.38756601	9847.991952	76.38756601
PLIPA2A140pp	66.3263	9319.145036	140.5045214	9319.145036	140.5045214
PLIPA2A141pp	66.3263	9319.146347	140.5045411	9319.146347	140.5045411
PLIPA2A180pp	66.3263	9300.042994	140.2165203	9300.042994	140.2165203
CU1Opp	56.5562	40900.17532	723.1775706	40900.17532	723.1775706
SUCDi	355.0752	383163.8026	1079.106067	383163.8026	1079.106067
LPLIPAL1E141pp	94.4886	9609.131398	101.6961982	9609.131398	101.6961982
LALDO2x	309.6976	30808.04378	99.47782541	30808.04378	99.47782541

LPLIPAL1E160pp	94.4886	15013.90298	158.8964487	15013.90298	158.8964487
LPLIPAL1E180pp	94.4886	9300.057685	98.42518235	9300.057685	98.42518235
LPLIPAL1E181pp	94.4886	9300.051662	98.42511861	9300.051662	98.42511861
LPLIPAL1G160pp	94.4886	9751.655563	103.2045724	9751.655563	103.2045724
LPLIPAL1G161pp	94.4886	15394.04218	162.9195711	15394.04218	162.9195711
LPLIPAL1G120pp	94.4886	9607.452793	101.6784331	9607.452793	101.6784331
PLIPA2E140pp	66.3263	9652.145285	145.525158	9652.145285	145.525158
PLIPA2E160pp	66.3263	9379.556975	141.4153507	9379.556975	141.4153507
PLIPA2A161pp	66.3263	9331.848079	140.6960448	9331.848079	140.6960448
PLIPA2A181pp	66.3263	12083.64247	182.1847815	12083.64247	182.1847815
ALR4x	309.6976	45141.94591	145.7613682	45141.94591	145.7613682
LPLIPAL1G141pp	94.4886	9300.056692	98.42517184	9300.056692	98.42517184
LPLIPAL1G180pp	94.4886	14240.04696	150.7065081	14240.04696	150.7065081
LPLIPAL1G181pp	94.4886	9300.05235	98.42512589	9300.05235	98.42512589
ACONTb_num1	186.9962	102219.9572	546.6418955	102219.9572	546.6418955
ACONTb_num2	195.3544	102219.9572	523.2539283	102219.9572	523.2539283
GLCP2_num1	181.0448	256439.2924	1416.441082	256439.2924	1416.441082
GLCP2_num2	186.3451	256439.2924	1376.152592	256439.2924	1376.152592
PLIPA2E141pp	66.3263	11806.14603	178.0009744	11806.14603	178.0009744
PLIPA2E181pp	66.3263	9300.059374	140.2167673	9300.059374	140.2167673
PLIPA2G140pp	66.3263	9300.054092	140.2166877	9300.054092	140.2166877
3HAD161_num1	37.9381	9300.028072	245.1368959	9300.028072	245.1368959

3HAD161_num2	102.1977	9300.028072	91.00036568	9300.028072	91.00036568
PLIPA2G160pp	66.3263	9331.087626	140.6845795	9331.087626	140.6845795
PLIPA2G181pp	66.3263	9300.262651	140.2198321	9300.262651	140.2198321
ASNS1	125.318	11068.89723	88.32647532	11068.89723	88.32647532
EAR80y	111.4558	9300.029087	83.44140984	9300.029087	83.44140984
EAR120y	111.4558	9300.025246	83.44137538	9300.025246	83.44137538
3OAR181	102.2412	16993.44901	166.2094049	16993.44901	166.2094049
3HAD121_num1	37.9381	9300.039797	245.137205	9300.039797	245.137205
3HAD121_num2	102.1977	9300.039797	91.00048041	9300.039797	91.00048041
EAR121y	111.4558	9300.032309	83.44143875	9300.032309	83.44143875
GTHRDHpp	61.768	75119.99162	1216.163574	75119.99162	1216.163574
MDDCP2pp_num1	47.7524	26499.7787	554.9412952	26499.7787	554.9412952
MDDCP2pp_num2	87.2178	26499.7787	303.8345235	26499.7787	303.8345235
MDDCP2pp_num3	88.8879	26499.7787	298.1258271	26499.7787	298.1258271
MDDCP2pp_num4	51.7983	26499.7787	511.5955293	26499.7787	511.5955293
MDDCP2pp_num5	43.3461	26499.7787	611.3532406	26499.7787	611.3532406
MDDCP2pp_num6	41.849	26499.7787	633.223702	26499.7787	633.223702
MDDCP3pp_num1	47.7524	26609.07923	557.2301964	26609.07923	557.2301964
MDDCP3pp_num2	87.2178	26609.07923	305.0877141	26609.07923	305.0877141
MDDCP3pp_num3	88.8879	26609.07923	299.3554717	26609.07923	299.3554717
MDDCP3pp_num4	51.7983	26609.07923	513.7056473	26609.07923	513.7056473
MDDCP3pp_num5	43.3461	26609.07923	613.8748176	26609.07923	613.8748176

MDDCP3pp_num6	41.849	26609.07923	635.8354855	26609.07923	635.8354855
EAR140x	111.4558	18506.45411	166.0429884	18506.45411	166.0429884
EAR40y	111.4558	15779.792	141.5789219	15779.792	141.5789219
3OAS181	86.0915	13204.15682	153.3735249	13204.15682	153.3735249
EAR161y	111.4558	9387.626342	84.22734701	9387.626342	84.22734701
G3PAT140	100.021	21089.53063	210.8510276	21089.53063	210.8510276
G3PAT160	100.021	19657.24406	196.5311691	19657.24406	196.5311691
G3PAT161	100.021	21670.12086	216.6557109	21670.12086	216.6557109
AGPAT160	27.4532	489600	17833.98657	489600	17833.98657
CDAPPA140	28.4508	9300.229927	326.8881693	9300.229927	326.8881693
MLDCP1App_num1	36.0824	12050.76394	333.9790019	12050.76394	333.9790019
MLDCP1App_num2	34.4111	12050.76394	350.1999046	12050.76394	350.1999046
MLDCP1App_num3	67.8125	12050.76394	177.707118	12050.76394	177.707118
MLDCP1App_num4	34.6359	12050.76394	347.9269757	12050.76394	347.9269757
MLDCP1App_num5	33.3253	12050.76394	361.6100661	12050.76394	361.6100661
EAR160y	111.4558	9300.034724	83.44146042	9300.034724	83.44146042
EAR100y	111.4558	9300.030649	83.44142386	9300.030649	83.44142386
3OAR141	102.2412	9300.034418	90.96171033	9300.034418	90.96171033
3OAR161	102.2412	9300.055921	90.96192065	9300.055921	90.96192065
3HAD141_num1	37.9381	9300.023712	245.136781	9300.023712	245.136781
3HAD141_num2	102.1977	9300.023712	91.00032302	9300.023712	91.00032302
EAR121x	111.4558	14092.40007	126.4393605	14092.40007	126.4393605

PSD140	35.9344	23485.34589	653.5616538	23485.34589	653.5616538
MICITDr	186.9962	24735.85224	132.2799727	24735.85224	132.2799727
CDAPPA181	28.4508	32332.59317	1136.438806	32332.59317	1136.438806
PGSA140	20.7006	9537.624374	460.7414459	9537.624374	460.7414459
LPLIPAL1A141pp	94.4886	10375.35393	109.8053515	10375.35393	109.8053515
LPLIPAL1A180pp	94.4886	9300.046054	98.42505925	9300.046054	98.42505925
LPLIPAL1E140pp	94.4886	9607.460155	101.678511	9607.460155	101.678511
LPLIPAL1E161pp	94.4886	13796.97292	146.0173283	13796.97292	146.0173283
LPLIPAL1G140pp	94.4886	9300.039358	98.42498839	9300.039358	98.42498839
ACONTa_num1	186.9962	77882.09375	416.4902482	77882.09375	416.4902482
ACONTa_num2	195.3544	77882.09375	398.6707939	77882.09375	398.6707939
MLDCP2App_num1	67.8125	61771.37288	910.9142545	61771.37288	910.9142545
MLDCP2App_num2	36.0824	61771.37288	1711.952999	61771.37288	1711.952999
MLDCP2App_num3	34.6359	61771.37288	1783.449337	61771.37288	1783.449337
MLDCP2App_num4	33.3253	61771.37288	1853.587901	61771.37288	1853.587901
MLDCP2App_num5	34.4111	61771.37288	1795.100211	61771.37288	1795.100211
PGPP120pp_num1	19.4181	39502.05577	2034.29047	39502.05577	2034.29047
PGPP120pp_num2	29.0213	39502.05577	1361.14012	39502.05577	1361.14012
PGPP181pp_num1	19.4181	9508.83052	489.6890283	9508.83052	489.6890283
PGPP181pp_num2	29.0213	9508.83052	327.6500543	9508.83052	327.6500543
PGSA181	20.7006	9766.998331	471.8219922	9766.998331	471.8219922
PGPP140_num1	19.4181	9921.214537	510.9261224	9921.214537	510.9261224

PGPP140_num2	29.0213	9921.214537	341.859756	9921.214537	341.859756
PGPP141_num1	19.4181	9300.062779	478.9378353	9300.062779	478.9378353
PGPP141_num2	29.0213	9300.062779	320.4564502	9300.062779	320.4564502
MDDCP5pp_num1	47.7524	152010.8959	3183.314261	152010.8959	3183.314261
MDDCP5pp_num2	87.2178	152010.8959	1742.888446	152010.8959	1742.888446
MDDCP5pp_num3	88.8879	152010.8959	1710.141605	152010.8959	1710.141605
MDDCP5pp_num4	51.7983	152010.8959	2934.669592	152010.8959	2934.669592
MDDCP5pp_num5	43.3461	152010.8959	3506.910562	152010.8959	3506.910562
MDDCP5pp_num6	41.849	152010.8959	3632.366268	152010.8959	3632.366268
MLTGY3pp_num1	49.4175	26411.44996	534.4554047	26411.44996	534.4554047
MLTGY3pp_num2	40.1125	26411.44996	658.4344023	26411.44996	658.4344023
MLTGY3pp_num3	40.2558	26411.44996	656.09055	26411.44996	656.09055
MLTGY3pp_num4	73.3532	26411.44996	360.0585927	26411.44996	360.0585927
MLTGY3pp_num5	40.4106	26411.44996	653.5772783	26411.44996	653.5772783
MLTGY3pp_num6	22.2266	26411.44996	1188.281157	26411.44996	1188.281157
MLTGY3pp_num7	58.3022	26411.44996	453.0094913	26411.44996	453.0094913
MLTGY3pp_num8	38.2474	26411.44996	690.5423626	26411.44996	690.5423626
G3PAT180	100.021	9300.061724	92.98109121	9300.061724	92.98109121
AGPAT140	27.4532	11008.28694	400.9837445	11008.28694	400.9837445
PGSA160	20.7006	10557.80693	510.0241986	10557.80693	510.0241986
MLDCP2Bpp_num1	36.0824	71515.31566	1981.999968	71515.31566	1981.999968
MLDCP2Bpp_num2	33.3253	71515.31566	2145.97665	71515.31566	2145.97665

MLDCP2Bpp_num3	34.6359	71515.31566	2064.774285	71515.31566	2064.774285
MLDCP2Bpp_num4	34.4111	71515.31566	2078.262992	71515.31566	2078.262992
MLDCP2Bpp_num5	67.8125	71515.31566	1054.603733	71515.31566	1054.603733
3OAS140_num1	85.2266	15220.88429	178.5931187	15220.88429	178.5931187
3OAS140_num2	86.0915	15220.88429	176.7989208	15220.88429	176.7989208
3OAR140	102.2412	4680	45.77411063	4680	45.77411063
3OAR160	102.2412	9300.030959	90.96167649	9300.030959	90.96167649
PGSA161	20.7006	23465.32539	1133.557742	23465.32539	1133.557742
3HAD181_num1	37.9381	16748.6226	441.4723615	16748.6226	441.4723615
3HAD181_num2	102.1977	16748.6226	163.8845355	16748.6226	163.8845355
PGPP160_num1	19.4181	9509.750323	489.7363966	9509.750323	489.7363966
PGPP160_num2	29.0213	9509.750323	327.6817483	9509.750323	327.6817483
PGPP180_num1	19.4181	17375.97622	894.8340064	17375.97622	894.8340064
PGPP180_num2	29.0213	17375.97622	598.7318356	17375.97622	598.7318356
PGPP161_num1	19.4181	9547.694856	491.6904772	9547.694856	491.6904772
PGPP161_num2	29.0213	9547.694856	328.9892202	9547.694856	328.9892202
PGPP181_num1	29.0213	17233.97969	593.8389973	17233.97969	593.8389973
PGPP181_num2	19.4181	17233.97969	887.5214203	17233.97969	887.5214203
CDAPPA180	28.4508	63141.67922	2219.328779	63141.67922	2219.328779
NTP12	36.4253	9852.661984	270.4895219	9852.661984	270.4895219
PSD141	35.9344	26441.03704	735.8140679	26441.03704	735.8140679
PAPA181pp	29.0213	9300.075757	320.4568974	9300.075757	320.4568974

MDDCP4pp_num1	47.7524	11770.52921	246.4908405	11770.52921	246.4908405
MDDCP4pp_num2	87.2178	11770.52921	134.9555849	11770.52921	134.9555849
MDDCP4pp_num3	88.8879	11770.52921	132.4199268	11770.52921	132.4199268
MDDCP4pp_num4	51.7983	11770.52921	227.2377513	11770.52921	227.2377513
MDDCP4pp_num5	43.3461	11770.52921	271.5475951	11770.52921	271.5475951
MDDCP4pp_num6	41.849	11770.52921	281.261899	11770.52921	281.261899
CLPNS140pp_num1	54.8223	10794.75856	196.904518	10794.75856	196.904518
CLPNS140pp_num2	47.6335	10794.75856	226.6211502	10794.75856	226.6211502
CLPNS141pp_num1	54.8223	32295.07812	589.0865235	32295.07812	589.0865235
CLPNS141pp_num2	47.6335	32295.07812	677.9908703	32295.07812	677.9908703
CFAS160E	87.818	52560.31961	598.5141954	52560.31961	598.5141954
CFAS180E	87.818	1612231.501	18358.78181	1612231.501	18358.78181
CFAS180G	87.818	107777.6323	1227.284068	107777.6323	1227.284068
PAPA160	29.0213	16190.81334	557.8941445	16190.81334	557.8941445
PAPA180	29.0213	15173.4659	522.8389459	15173.4659	522.8389459
PAPA181	29.0213	19467.0968	670.786519	19467.0968	670.786519
PETNT161pp	63.8044	23599.62422	369.8745576	23599.62422	369.8745576
PSSA160	52.8018	54000	1022.692408	54000	1022.692408
PSSA181	52.8018	18305.1176	346.6760149	18305.1176	346.6760149
DAGK140	39.734	9418.972615	237.0507026	9418.972615	237.0507026
DAGK160	39.734	9418.996138	237.0512945	9418.996138	237.0512945
DAGK141	39.734	9300.306841	234.0641979	9300.306841	234.0641979
DAGK161	39.734	18097.76754	455.4730846	18097.76754	455.4730846
AGPAT181	27.4532	71164.94731	2592.227766	71164.94731	2592.227766
MDDEP3pp_num1	29.9166	33111.5862	1106.796434	33111.5862	1106.796434

MDDEP3pp_num 2	21.039 9	33111.5862	1573.752071	33111.5862	1573.752071
MDDEP3pp_num 3	60.273 3	33111.5862	549.3574469	33111.5862	549.3574469
MDDEP3pp_num 4	51.798 3	33111.5862	639.2407898	33111.5862	639.2407898
MDDEP3pp_num 5	33.887 4	33111.5862	977.106128	33111.5862	977.106128
MDDEP3pp_num 6	41.849	33111.5862	791.2157089	33111.5862	791.2157089
MDDEP3pp_num 7	49.057 8	33111.5862	674.9504911	33111.5862	674.9504911
MDDEP4pp_num 1	29.916 6	26327.99993	880.0465271	26327.99993	880.0465271
MDDEP4pp_num 2	41.849	26327.99993	629.1189738	26327.99993	629.1189738
MDDEP4pp_num 3	60.273 3	26327.99993	436.8103279	26327.99993	436.8103279
MDDEP4pp_num 4	51.798 3	26327.99993	508.279228	26327.99993	508.279228
MDDEP4pp_num 5	33.887 4	26327.99993	776.9259351	26327.99993	776.9259351
MDDEP4pp_num 6	21.039 9	26327.99993	1251.336743	26327.99993	1251.336743
MDDEP4pp_num 7	49.057 8	26327.99993	536.6730659	26327.99993	536.6730659
MLTGY1pp_num 1	49.417 5	31677.55986	641.0190693	31677.55986	641.0190693
MLTGY1pp_num 2	22.226 6	31677.55986	1425.209427	31677.55986	1425.209427
MLTGY1pp_num 3	40.410 6	31677.55986	783.8923416	31677.55986	783.8923416
MLTGY1pp_num 4	40.112 5	31677.55986	789.7179148	31677.55986	789.7179148
MLTGY1pp_num 5	73.353 2	31677.55986	431.8497333	31677.55986	431.8497333
MLTGY1pp_num 6	40.255 8	31677.55986	786.9067279	31677.55986	786.9067279
MLTGY1pp_num 7	58.302 2	31677.55986	543.3338683	31677.55986	543.3338683
MLTGY1pp_num 8	38.247 4	31677.55986	828.2277974	31677.55986	828.2277974
3HAD40_num1	37.938 1	13622.60957	359.0746392	13622.60957	359.0746392

3HAD40_num2	102.1977	13622.60957	133.2966355	13622.60957	133.2966355
PLIPA1E141pp	66.3263	9300.040567	140.2164838	9300.040567	140.2164838
3OAS60_num1	86.0915	14210.82631	165.0665433	14210.82631	165.0665433
3OAS60_num2	85.2266	14210.82631	166.7416782	14210.82631	166.7416782
3OAS160_num1	85.2266	9300.048851	109.1214345	9300.048851	109.1214345
3OAS160_num2	86.0915	9300.048851	108.0251692	9300.048851	108.0251692
3OAR60	102.2412	14676.62331	143.5490126	14676.62331	143.5490126
3OAR80	102.2412	9300.033619	90.96170252	9300.033619	90.96170252
MLDCP3App_num1	36.0824	11472.2901	317.9469798	11472.2901	317.9469798
MLDCP3App_num2	34.4111	11472.2901	333.3892292	11472.2901	333.3892292
MLDCP3App_num3	67.8125	11472.2901	169.1766283	11472.2901	169.1766283
MLDCP3App_num4	34.6359	11472.2901	331.2254078	11472.2901	331.2254078
MLDCP3App_num5	33.3253	11472.2901	344.2516678	11472.2901	344.2516678
MLDCP1Bpp_num1	36.0824	38207.63549	1058.899505	38207.63549	1058.899505
MLDCP1Bpp_num2	34.4111	38207.63549	1110.328803	38207.63549	1110.328803
MLDCP1Bpp_num3	67.8125	38207.63549	563.4305694	38207.63549	563.4305694
MLDCP1Bpp_num4	34.6359	38207.63549	1103.122352	38207.63549	1103.122352
MLDCP1Bpp_num5	33.3253	38207.63549	1146.505372	38207.63549	1146.505372
PLIPA1G160pp	66.3263	9331.083133	140.6845118	9331.083133	140.6845118
PLIPA1G141pp	66.3263	9300.055363	140.2167068	9300.055363	140.2167068
LPLIPAL2E160	38.9783	22686.53673	582.0299173	22686.53673	582.0299173
LPLIPAL2G140	38.9783	16311.88717	418.4863673	16311.88717	418.4863673

LPLIPAL2G160	38.9783	10136.74444	260.0612248	10136.74444	260.0612248
LPLIPAL2G120	38.9783	9300.050474	238.5955897	9300.050474	238.5955897
PGPP161pp_num1	19.4181	9508.609454	489.6776438	9508.609454	489.6776438
PGPP161pp_num2	29.0213	9508.609454	327.6424369	9508.609454	327.6424369
PAPA140	29.0213	33006.15625	1137.307986	33006.15625	1137.307986
PAPA141	29.0213	9542.900549	328.8240206	9542.900549	328.8240206
PAPA161	29.0213	17767.63809	612.2275048	17767.63809	612.2275048
DSBAO1	43.2467	20873.21805	482.6545853	20873.21805	482.6545853
DSBAO2	43.2467	11874.51671	274.5762499	11874.51671	274.5762499
DSBDR_num1	77.3499	37200.94625	480.943689	37200.94625	480.943689
DSBDR_num2	73.6017	37200.94625	505.4359648	37200.94625	505.4359648
PLIPA2A120pp	66.3263	9319.130597	140.5043037	9319.130597	140.5043037
PLIPA2A160pp	66.3263	14809.58551	223.2837579	14809.58551	223.2837579
LPLIPAL2E120	38.9783	9300.089592	238.5965933	9300.089592	238.5965933
EAR100x	111.4558	13324.87919	119.5530353	13324.87919	119.5530353
EAR120x	111.4558	15973.78694	143.3194768	15973.78694	143.3194768
EAR160x	111.4558	9300.034296	83.44145658	9300.034296	83.44145658
EAR140y	111.4558	15246.01464	136.7897825	15246.01464	136.7897825
3OAS121	85.2266	9300.067402	109.1216522	9300.067402	109.1216522
3OAS80_num1	85.2266	9300.057325	109.1215339	9300.057325	109.1215339
3OAS80_num2	86.0915	9300.057325	108.0252676	9300.057325	108.0252676
3OAS100_num1	85.2266	9300.069292	109.1216744	9300.069292	109.1216744

3OAS100_num2	86.091 5	9300.069292	108.0254066	9300.069292	108.0254066
3OAS120_num1	86.091 5	9300.044909	108.0251234	9300.044909	108.0251234
3OAS120_num2	85.226 6	9300.044909	109.1213883	9300.044909	109.1213883
3OAR40	102.24 12	11866.89778	116.0676692	11866.89778	116.0676692
3OAR100	102.24 12	9300.038342	90.96174871	9300.038342	90.96174871
3HAD60_num1	37.938 1	35394.22481	932.9466898	35394.22481	932.9466898
3HAD60_num2	102.19 77	35394.22481	346.3309332	35394.22481	346.3309332
3HAD100_num1	37.938 1	9300.031517	245.1369867	9300.031517	245.1369867
3HAD100_num2	102.19 77	9300.031517	91.00039939	9300.031517	91.00039939
3HAD140_num1	37.938 1	9689.000825	255.3897223	9689.000825	255.3897223
3HAD140_num2	102.19 77	9689.000825	94.80644696	9689.000825	94.80644696
3HAD160_num1	37.938 1	9300.049708	245.1374662	9300.049708	245.1374662
3HAD160_num2	102.19 77	9300.049708	91.00057739	9300.049708	91.00057739
AGPAT180	27.453 2	9300.222929	338.7664436	9300.222929	338.7664436
AGPAT141	27.453 2	18651.92002	679.4078658	18651.92002	679.4078658
AGPAT161	27.453 2	576000	20981.16067	576000	20981.16067
DASYN120	31.453 8	9544.492519	303.4448149	9544.492519	303.4448149
EAR40x	111.45 58	44179.31434	396.3841661	44179.31434	396.3841661
EAR60y	111.45 58	14300.64672	128.3077841	14300.64672	128.3077841
MLDEP1pp	60.273 3	42680.98235	708.1242001	42680.98235	708.1242001
MLDEP2pp	60.273 3	29895.31519	495.9959915	29895.31519	495.9959915
MLTGY2pp_num 1	49.417 5	72155.37283	1460.117829	72155.37283	1460.117829

MLTGY2pp_num2	22.2266	72155.37283	3246.352246	72155.37283	3246.352246
MLTGY2pp_num3	40.4106	72155.37283	1785.555593	72155.37283	1785.555593
MLTGY2pp_num4	40.1125	72155.37283	1798.825125	72155.37283	1798.825125
MLTGY2pp_num5	73.3532	72155.37283	983.670417	72155.37283	983.670417
MLTGY2pp_num6	40.2558	72155.37283	1792.421783	72155.37283	1792.421783
MLTGY2pp_num7	58.3022	72155.37283	1237.609779	72155.37283	1237.609779
MLTGY2pp_num8	38.2474	72155.37283	1886.543212	72155.37283	1886.543212
MLTGY4pp_num1	49.4175	14600.99625	295.4620581	14600.99625	295.4620581
MLTGY4pp_num2	40.1125	14600.99625	364.0011531	14600.99625	364.0011531
MLTGY4pp_num3	40.2558	14600.99625	362.7054053	14600.99625	362.7054053
MLTGY4pp_num4	73.3532	14600.99625	199.0505698	14600.99625	199.0505698
MLTGY4pp_num5	40.4106	14600.99625	361.3159977	14600.99625	361.3159977
MLTGY4pp_num6	22.2266	14600.99625	656.9154191	14600.99625	656.9154191
MLTGY4pp_num7	58.3022	14600.99625	250.4364545	14600.99625	250.4364545
MLTGY4pp_num8	38.2474	14600.99625	381.7513414	14600.99625	381.7513414
EAR60x	111.4558	13560.4688	121.6667845	13560.4688	121.6667845
EAR80x	111.4558	14706.89696	131.9527289	14706.89696	131.9527289
NTP11	36.4253	10027.83356	275.2985852	10027.83356	275.2985852
3OAS180_num1	86.0915	16822.95266	195.4078237	16822.95266	195.4078237
3OAS180_num2	85.2266	16822.95266	197.3908693	16822.95266	197.3908693
3HAD180_num1	37.9381	9449.861839	249.0863232	9449.861839	249.0863232
3HAD180_num2	102.1977	9449.861839	92.46648251	9449.861839	92.46648251

CDAPPA120	28.4508	31900.48775	1121.250993	31900.48775	1121.250993
PSSA120	52.8018	21806.89222	412.9952429	21806.89222	412.9952429
PSD120	35.9344	38651.46836	1075.611903	38651.46836	1075.611903
PAPA120	29.0213	36805.00327	1268.206568	36805.00327	1268.206568
MDDEP1pp_num1	21.0399	30020.38301	1426.831069	30020.38301	1426.831069
MDDEP1pp_num2	29.9166	30020.38301	1003.469078	30020.38301	1003.469078
MDDEP1pp_num3	60.2733	30020.38301	498.0710034	30020.38301	498.0710034
MDDEP1pp_num4	51.7983	30020.38301	579.563094	30020.38301	579.563094
MDDEP1pp_num5	33.8874	30020.38301	885.8862885	30020.38301	885.8862885
MDDEP1pp_num6	49.0578	30020.38301	611.9390395	30020.38301	611.9390395
MDDEP1pp_num7	41.849	30020.38301	717.3500684	30020.38301	717.3500684
MDDEP2pp_num1	21.0399	194468.9023	9242.862479	194468.9023	9242.862479
MDDEP2pp_num2	29.9166	194468.9023	6500.367765	194468.9023	6500.367765
MDDEP2pp_num3	60.2733	194468.9023	3226.451883	194468.9023	3226.451883
MDDEP2pp_num4	51.7983	194468.9023	3754.349125	194468.9023	3754.349125
MDDEP2pp_num5	33.8874	194468.9023	5738.67875	194468.9023	5738.67875
MDDEP2pp_num6	49.0578	194468.9023	3964.077115	194468.9023	3964.077115
MDDEP2pp_num7	41.849	194468.9023	4646.918738	194468.9023	4646.918738
PSD181	35.9344	18069.53214	502.8477485	18069.53214	502.8477485
DAGK180	39.734	9300.360269	234.0655426	9300.360269	234.0655426
DAGK181	39.734	13406.26531	337.4003451	13406.26531	337.4003451
PLIPA2E120pp	66.3263	10490.57744	158.166179	10490.57744	158.166179
PLIPA2G141pp	66.326	9300.051727	140.216652	9300.051727	140.216652

	3				
PLIPA2G161pp	66.3263	11859.91282	178.811615	11859.91282	178.811615
LPLIPAL1A120pp	94.4886	9607.451501	101.6784194	9607.451501	101.6784194
LPLIPAL1A140pp	94.4886	21414.67088	226.6376142	21414.67088	226.6376142
LPLIPAL1A181pp	94.4886	15755.14779	166.7412555	15755.14779	166.7412555
LPLIPAL1E120pp	94.4886	18049.24557	191.0203514	18049.24557	191.0203514
3OAR180	102.2412	9346.605887	91.41721622	9346.605887	91.41721622
G3PAT120	100.021	9886.455119	98.84379399	9886.455119	98.84379399
PGSA120	20.7006	9537.672938	460.7437919	9537.672938	460.7437919
NADHPO	319.9686	10193.77953	31.85868715	10193.77953	31.85868715
ACOAD7f	89.2244	168849.2482	1892.411137	168849.2482	1892.411137
ACOAD8f	89.2244	76148.30066	853.4470466	76148.30066	853.4470466
ACOAD5f	89.2244	223003.5589	2499.356217	223003.5589	2499.356217
ACOAD3f	89.2244	374885.7826	4201.606092	374885.7826	4201.606092
ACOAD2f	89.2244	426233.6705	4777.097638	426233.6705	4777.097638
ACOAD4f	89.2244	185797.065	2082.357124	185797.065	2082.357124
ACOAD6f	89.2244	208640.9014	2338.383911	208640.9014	2338.383911
NADH16pp	540.4142	700176.037	1295.628496	5400000	9992.335509
NADH18pp	540.4142	12049.1586	22.29615469	12049.1586	22.29615469
CTBTCAL2	58.5591	10877.64559	185.7549995	10877.64559	185.7549995
BGLA1_num1	53.9349	37406.12216	693.5420695	37406.12216	693.5420695
BGLA1_num2	202.05	37406.12216	185.1329976	37406.12216	185.1329976
BGLA1_num3	53.161	37406.12216	703.6344515	37406.12216	703.6344515

	3				
GDPMNP	43.497<br>3	58494.91914	1344.794255	58494.91914	1344.794255
AMMQLT8	28.073<br>2	25407.58414	905.047666	25407.58414	905.047666
FA120ACPHi	22.961<br>1	17684.13687	770.1781217	17684.13687	770.1781217
FA80ACPHi	22.961<br>1	9507.786649	414.0823675	9507.786649	414.0823675
FA140ACPHi	22.961<br>1	100253.0484	4366.212784	100253.0484	4366.212784
FA100ACPHi	22.961<br>1	9507.909798	414.0877309	9507.909798	414.0877309
CRNDCAL2	58.559<br>1	20216.42014	345.2310596	20216.42014	345.2310596
CRNCAL2	58.559<br>1	10143.28302	173.2144623	10143.28302	173.2144623
FRULYSE	249.35<br>06	20347.52166	81.60205614	20347.52166	81.60205614
OXCDC	242.32<br>49	11287.64711	46.58063248	11287.64711	46.58063248
LPLIPAL2A140	38.978<br>3	9300.044372	238.5954332	9300.044372	238.5954332
LPLIPAL2A160	38.978<br>3	10123.72369	259.7271736	10123.72369	259.7271736
LPLIPAL2A161	38.978<br>3	25406.02561	651.7992219	25406.02561	651.7992219
LPLIPAL2A180	38.978<br>3	18839.70967	483.3384132	18839.70967	483.3384132
LPLIPAL2A181	38.978<br>3	13091.78521	335.8736838	13091.78521	335.8736838
UM3PL	49.874<br>1	9421.28154	188.9012842	9421.28154	188.9012842
UM4PL	49.874<br>1	9308.520536	186.6403712	9308.520536	186.6403712
2MAHMP_num1	30.370<br>9	10170.48834	334.8760931	10170.48834	334.8760931
2MAHMP_num2	17.432<br>9	10170.48834	583.4077139	10170.48834	583.4077139
PLIPA1A120pp	66.326<br>3	9300.040452	140.216482	9300.040452	140.216482
PLIPA1A140pp	66.326<br>3	9300.04132	140.2164951	9300.04132	140.2164951

PLIPA1A141pp	66.3263	9300.070328	140.2169325	9300.070328	140.2169325
PLIPA1A160pp	66.3263	12745.09621	192.1575032	12745.09621	192.1575032
PLIPA1A161pp	66.3263	21566.39233	325.1559687	21566.39233	325.1559687
PLIPA1A180pp	66.3263	12246.46773	184.639694	12246.46773	184.639694
LPLIPAL2A120	38.9783	9300.07543	238.5962299	9300.07543	238.5962299
KDUI	186.4581	45006.1627	241.3741355	45006.1627	241.3741355
NADH17pp	540.4142	134714.4577	249.280011	134714.4577	249.280011
FA161ACPHi	22.9611	72014.93431	3136.388688	72014.93431	3136.388688
FA141ACPHi	22.9611	467514.5519	20361.15656	467514.5519	20361.15656
FA160ACPHi	22.9611	34673.13241	1510.081503	34673.13241	1510.081503
DTARTD	120.2106	45441.45666	378.0153885	45441.45666	378.0153885
PLIPA2E161pp	66.3263	9331.080116	140.6844663	9331.080116	140.6844663
PLIPA2E180pp	66.3263	9300.046288	140.21657	9300.046288	140.21657
PLIPA2G120pp	66.3263	9331.096327	140.6847107	9331.096327	140.6847107
PLIPA2G180pp	66.3263	11670.73496	175.9593851	11670.73496	175.9593851
LPLIPAL1A161pp	94.4886	14398.00521	152.3782256	14398.00521	152.3782256
LPLIPAL1A160pp	94.4886	313499.2089	3317.852195	313499.2089	3317.852195
GALCTLO	36.4479	21845.1942	599.3539875	21845.1942	599.3539875
THRA_num1	90.6332	53168.68951	586.6359073	53168.68951	586.6359073
THRA_num2	145.9788	53168.68951	364.2219933	53168.68951	364.2219933
XYLK2_num1	122.1788	44532.4874	364.4862071	44532.4874	364.4862071
XYLK2_num2	110.3097	44532.4874	403.7041838	44532.4874	403.7041838

GLDBRAN2	73.5767	31696.23254	430.7917118	31696.23254	430.7917118
POR5_num1	148.5614	16213.79769	109.1386975	16213.79769	109.1386975
POR5_num2	148.5246	16213.79769	109.1657388	16213.79769	109.1657388
HACD8_num1	159.1878	30181.25367	189.5952684	30181.25367	189.5952684
HACD8_num2	77.0722	30181.25367	391.5971475	30181.25367	391.5971475
CTECOAI8	159.1878	29862.20361	187.5910315	29862.20361	187.5910315
CTECOAI6	159.1878	24446.50672	153.5702278	24446.50672	153.5702278
MALCOAMT	28.2762	9301.412639	328.9484669	9301.412639	328.9484669
OGMEACPS	67.0302	0.2448	0.003652085	0.2448	0.003652085
OGMEACPR	102.2412	0.1224	0.001197169	0.1224	0.001197169
OPMEACPS	85.2266	0.0864	0.001013768	0.0864	0.001013768
DGUNC_num1	51.7825	17685.97519	341.5434788	17685.97519	341.5434788
DGUNC_num2	38.5456	17685.97519	458.8325306	17685.97519	458.8325306
DGUNC_num3	185.8088	17685.97519	95.1837329	17685.97519	95.1837329
DGUNC_num4	67.727	17685.97519	261.1362557	17685.97519	261.1362557
DGUNC_num5	106.3554	17685.97519	166.2912761	17685.97519	166.2912761
CDGUNPD_num1	74.665	126921.3273	1699.877149	126921.3273	1699.877149
CDGUNPD_num2	180.5208	126921.3273	703.0842282	126921.3273	703.0842282
CDGUNPD_num3	171.2153	126921.3273	741.2966443	126921.3273	741.2966443
CDGUNPD_num4	29.6012	126921.3273	4287.708854	126921.3273	4287.708854
CDGUNPD_num5	81.4498	126921.3273	1558.276722	126921.3273	1558.276722
LDGUNPD	74.665	44519.35518	596.2546733	44519.35518	596.2546733
MPTAT	63.667	2448	38.45006047	2448	38.45006047

MOADSUx	107.6949	36042.55733	334.6728334	36042.55733	334.6728334
REPHACCOAI	28.4046	19156.91578	674.4300494	19156.91578	674.4300494
ACPPAT141	46.854	36607.03398	781.3000807	36607.03398	781.3000807
ACPPAT161	46.854	40006.80572	853.8610519	40006.80572	853.8610519
ACPPAT180	46.854	9699.07613	207.006363	9699.07613	207.006363
APG3PAT160	22.1934	18407.65507	829.4202361	18407.65507	829.4202361
APG3PAT161	22.1934	9694.949292	436.8392987	9694.949292	436.8392987
APG3PAT180	22.1934	47472.73186	2139.047278	47472.73186	2139.047278
ICYSDS	90.179	36	0.399206024	36	0.399206024
I4FE4SR	13.8486	7.2	0.51990815	7.2	0.51990815
S2FE2ST	183.333	9301.245664	50.7341595	9301.245664	50.7341595
BMOCOS	88.1345	72	0.81693321	72	0.81693321
BWCOS	88.1345	10232.07313	116.096116	10232.07313	116.096116
BWCOGDS1	21.6429	25790.66276	1191.645424	25790.66276	1191.645424
BWCOGDS2	21.6429	29065.07453	1342.938078	29065.07453	1342.938078
DHNPTE	108.9562	19512.83723	179.0888195	19512.83723	179.0888195
DHMPTR	26.3483	37100.23214	1408.069293	37100.23214	1408.069293
I2FE2SS	104.0276	9421.285561	90.56524962	9421.285561	90.56524962
I2FE2ST	60.0728	9353.641428	155.7051016	9353.641428	155.7051016
I2FE2SS2	104.0276	9497.407543	91.29699756	9497.407543	91.29699756
I4FE4ST	60.0728	9505.115485	158.2266098	9505.115485	158.2266098
S4FE4ST	183.333	45270.43078	246.9300714	45270.43078	246.9300714
S2FE2SS2	277.201	25109.73651	90.58313825	25109.73651	90.58313825
BTS5	77.296	7.2	0.093148175	7.2	0.093148175

	2				
3AMACHYD	28.8981	21702.14096	750.9885066	21702.14096	750.9885066
CCGS	25.5142	18471.57179	723.9722112	18471.57179	723.9722112
CDGR	65.175	18930.24966	290.4526224	18930.24966	290.4526224
MSAR_num1	21.5704	16893.06136	783.1593924	16893.06136	783.1593924
MSAR_num2	108.9957	16893.06136	154.9883285	16893.06136	154.9883285
ACPPAT120	46.854	14929.35902	318.6357411	14929.35902	318.6357411
CPH4S	82.6373	15951.34398	193.0283779	15951.34398	193.0283779
SHCHCS3	27.6824	47273.15239	1707.697035	47273.15239	1707.697035
URACPAH	25.2087	23399.76966	928.2418236	23399.76966	928.2418236
POAACR	13.7629	17716.71901	1287.280952	17716.71901	1287.280952
ACPPAT160	46.854	20129.32772	429.6181269	20129.32772	429.6181269
ACPPAT181	46.854	14737.83642	314.5480946	14737.83642	314.5480946
APH141	10.2998	9504.151621	922.7510846	9504.151621	922.7510846
APH161	10.2998	10164.79521	986.8924847	10164.79521	986.8924847
APH180	10.2998	11053.15404	1073.142589	11053.15404	1073.142589
APH181	10.2998	17581.02807	1706.929073	17581.02807	1706.929073
GTHPi	20.4696	21215.21356	1036.425409	21215.21356	1036.425409
SEPHCHCS	122.7341	24093.61572	196.3074298	24093.61572	196.3074298
DHNCOAS	189.7985	26258.64932	138.3501414	26258.64932	138.3501414
DHNCOAT	59.781	11519.68616	192.6981174	11519.68616	192.6981174
I2FE2SR	104.0276	9301.724046	89.41592468	9301.724046	89.41592468
TYRL	26.8961	21.6	0.803090411	21.6	0.803090411
THZPSN3	600.7306	9779.036108	16.27857164	9779.036108	16.27857164
APG3PAT120	22.193	9785.075695	440.9002539	9785.075695	440.9002539

	4				
APH120	10.2998	12612.9599	1224.582992	12612.9599	1224.582992
MOCDS	21.5138	18106.42146	841.6189356	18106.42146	841.6189356
FORCT	91.6564	37350.27356	407.5031701	37350.27356	407.5031701
PACCOAE	131.963	22170.16111	168.0028577	22170.16111	168.0028577
OXDHCOAT	70.6812	25910.35692	366.580603	25910.35692	366.580603
HADPCOADH3	51.7326	21560.11817	416.7607692	21560.11817	416.7607692
PYROX	59.9682	22380.99194	373.214336	22380.99194	373.214336
BMOGDS1	21.6429	27337.89485	1263.134554	27337.89485	1263.134554
MPTS	51.478	9753.689326	189.4729656	9753.689326	189.4729656
MOCOS	88.1345	72	0.81693321	72	0.81693321
BMOGDS2	21.6429	10255.58751	473.8545903	10255.58751	473.8545903
S2FE2SR	277.201	21311.91823	76.88254454	21311.91823	76.88254454
ACACT8r_num1	46.5305	23737.87625	510.157343	23737.87625	510.157343
ACACT8r_num2	81.7524	23737.87625	290.3630505	23737.87625	290.3630505
CTECOAI7	159.1878	24212.59768	152.1008374	24212.59768	152.1008374
FDH4pp_num1	170.1061	32853.99749	193.1382678	32853.99749	193.1382678
FDH4pp_num2	511.2616	32853.99749	64.26063975	32853.99749	64.26063975
FDH5pp_num1	511.2616	29922.56645	58.5269194	29922.56645	58.5269194
FDH5pp_num2	170.1061	29922.56645	175.9053112	29922.56645	175.9053112
ACPPAT140	46.854	9697.481201	206.9723226	9697.481201	206.9723226
APG3PAT140	22.1934	38886.36066	1752.15878	38886.36066	1752.15878
APG3PAT141	22.1934	25623.64564	1154.56152	25623.64564	1154.56152

APG3PAT181	22.1934	9481.372614	427.2158666	9481.372614	427.2158666
APH140	10.2998	9504.202723	922.7560461	9504.202723	922.7560461
APH160	10.2998	19803.85527	1922.74173	19803.85527	1922.74173
OPMEACPD	102.1977	0.0792	0.000774969	0.0792	0.000774969
EPMEACPR	111.4558	0.108	0.000968994	0.108	0.000968994
AOXSr2	83.1885	3.6	0.043275212	3.6	0.043275212
SCYSDS	120.4682	30061.01064	249.5348203	30061.01064	249.5348203
S2FE2SS	277.201	9300.815543	33.55260458	9300.815543	33.55260458
S4FE4SR	156.7328	74931.37607	478.0835669	74931.37607	478.0835669
SELR	233.0425	13273.52372	56.95752373	13273.52372	56.95752373
OGMEACPD	102.1977	0.0792	0.000774969	0.0792	0.000774969
EGMEACPR	111.4558	0.108	0.000968994	0.108	0.000968994
OPMEACPR	102.2412	0.1224	0.001197169	0.1224	0.001197169
PMEACPE	28.505	14.4	0.505174531	14.4	0.505174531
MECDPDH5_num1	60.3838	9667.011089	160.0927913	9667.011089	160.0927913
MECDPDH5_num2	60.4206	9667.011089	159.9952845	9667.011089	159.9952845
RNTR1c2_num1	179.7821	49229.87641	273.8308008	49229.87641	273.8308008
RNTR1c2_num2	242.4248	49229.87641	203.0727731	49229.87641	203.0727731
RNTR1c2_num3	242.3881	49229.87641	203.1035204	49229.87641	203.1035204
RNTR1c2_num4	179.7453	49229.87641	273.8868633	49229.87641	273.8868633
RNTR2c2_num1	242.4248	30111.14447	124.2081853	30111.14447	124.2081853
RNTR2c2_num2	242.3881	30111.14447	124.2269916	30111.14447	124.2269916

RNTR2c2_num3	179.7453	30111.14447	167.5211784	30111.14447	167.5211784
RNTR2c2_num4	179.7821	30111.14447	167.4868881	30111.14447	167.4868881
RNTR3c2_num1	179.7453	34677.14107	192.9237709	34677.14107	192.9237709
RNTR3c2_num2	242.4248	34677.14107	143.0428779	34677.14107	143.0428779
RNTR3c2_num3	242.3881	34677.14107	143.064536	34677.14107	143.064536
RNTR3c2_num4	179.7821	34677.14107	192.8842809	34677.14107	192.8842809
RNTR4c2_num1	179.7821	16403.76147	91.24246223	16403.76147	91.24246223
RNTR4c2_num2	242.4248	16403.76147	67.66536043	16403.76147	67.66536043
RNTR4c2_num3	242.3881	16403.76147	67.67560565	16403.76147	67.67560565
RNTR4c2_num4	179.7453	16403.76147	91.26114268	16403.76147	91.26114268
FLDR2_num1	47.4511	47286.67079	996.534765	47286.67079	996.534765
FLDR2_num2	47.4879	47286.67079	995.7625161	47286.67079	995.7625161
FESR	24.8826	71840.53476	2887.179586	71840.53476	2887.179586
OXCOAHDH	73.0026	73883.66933	1012.069013	73883.66933	1012.069013
DHACOAH	27.2374	23116.39998	848.7006831	23116.39998	848.7006831
THFAT	90.6332	30546.82446	337.0379117	30546.82446	337.0379117
FOMETRi	21.1054	13204.69489	625.6548034	13204.69489	625.6548034
GDPTPDP	79.3424	184851.3609	2329.792909	184851.3609	2329.792909
COLIPAKpp	26.7591	30763.48947	1149.645895	30763.48947	1149.645895
MPTSS	53.4377	6120	114.5258872	6120	114.5258872
CPMPS	142.1499	19871.43998	139.7921489	19871.43998	139.7921489
LIPOCT	23.8825	9300.465425	389.4259573	9300.465425	389.4259573

LIPOS	72.1434	9300.554147	128.9176023	9300.554147	128.9176023
OCTNLL	37.9259	22382.26906	590.1578884	22382.26906	590.1578884
LIPATPT	37.9259	25688.03264	677.3216361	25688.03264	677.3216361
LIPAMPL	37.9259	15754.36386	415.3985497	15754.36386	415.3985497
ALDD19xr	214.7965	24043.86239	111.9378686	24043.86239	111.9378686
MMM	155.7425	728300.542	4676.31213	728300.542	4676.31213
MOGDS	21.6429	20487.08051	946.595905	20487.08051	946.595905
WCOS	88.1345	78540.19837	891.1402274	78540.19837	891.1402274
VPAMTr	46.7112	20916.2244	447.7775009	20916.2244	447.7775009
3OXCOAT	42.2765	24123.2729	570.6071435	24123.2729	570.6071435
2DGULRGx	70.791	17496.28312	247.1540609	17496.28312	247.1540609
2DGULRGy	70.791	9611.080776	135.766987	9611.080776	135.766987
AP4AS	115.6531	23738.36358	205.2548836	23738.36358	205.2548836
13PPDH2	84.194	9783.379271	116.2004332	9783.379271	116.2004332
APCS	64.6428	13709.81887	212.0857832	13709.81887	212.0857832
GHBDHx	124.6326	21755.70782	174.5587256	21755.70782	174.5587256
APPLDHr	309.6976	10147.77107	32.76670877	10147.77107	32.76670877
G2PP	29.1721	10610.83138	363.732175	10610.83138	363.732175
DHNPA2r	108.9562	6120	56.1693598	6120	56.1693598
SHGO	46.0819	22357.77247	485.17471	22357.77247	485.17471
NADHXD	54.6503	65061.63673	1190.508318	65061.63673	1190.508318
NADPHHR	142.13	36321.85019	255.5537197	36321.85019	255.5537197
NADPHHS	142.13	34672.70059	243.9506128	34672.70059	243.9506128
NADHHS	142.13	309125.5263	2174.949176	309125.5263	2174.949176
NADHXE	54.650	13737.72351	251.3750795	13737.72351	251.3750795

	3				
PYK2	205.42 86	27680.85888	134.7468604	27680.85888	134.7468604
PYK3	205.42 86	45020.63808	219.1546751	45020.63808	219.1546751
PYK4	205.42 86	23979.27789	116.7280403	23979.27789	116.7280403
NADPHXD	54.650 3	10822.86747	198.0385737	10822.86747	198.0385737
NADHHR	142.13	18063.29674	127.0899651	18063.29674	127.0899651
NADPHXE	54.650 3	21725.95795	397.5450812	21725.95795	397.5450812
FE3DHBZS3R	28.871 6	10690.26984	370.2693943	10690.26984	370.2693943
DXYLTD_num1	69.399 2	77138.03722	1111.511908	77138.03722	1111.511908
DXYLTD_num2	70.016 8	77138.03722	1101.70755	77138.03722	1101.70755
2DDARAA_num1	32.721 7	28422.21991	868.6046236	28422.21991	868.6046236
2DDARAA_num2	130.12 16	28422.21991	218.4281465	28422.21991	218.4281465
GNP_num1	24.664 3	22285.81762	903.5657861	22285.81762	903.5657861
GNP_num2	20.779 8	22285.81762	1072.475078	22285.81762	1072.475078
ALLTAMH2	91.388	218031.9588	2385.783241	218031.9588	2385.783241
UGCIAMH	28.730 4	257942.8563	8978.046124	257942.8563	8978.046124
METGLCUR	273.78 8	48441.85139	176.9319743	48441.85139	176.9319743
ACOAD1fr	43.002 1	42460.6505	987.4087662	42460.6505	987.4087662
FEENTERES	45.651 9	22628.10623	495.6662533	22628.10623	495.6662533
FEENTERR4	28.871 6	16687.14684	577.9779037	16687.14684	577.9779037
ALR2x	36.328 8	36956.8215	1017.287152	36956.8215	1017.287152
OMPHHXy	42.288 3	15108.67816	357.2779743	15108.67816	357.2779743
SQVOSI	284.59 58	53182.41289	186.8699851	53182.41289	186.8699851

6D6SPA	31.983	39442.55994	1233.235154	39442.55994	1233.235154
AI2K	57.5448	9941.061582	172.7534301	9941.061582	172.7534301
ACOXT	83.3415	16076.64152	192.9007939	16076.64152	192.9007939
2HESR_num1	64.7832	27159.73869	419.2404619	27159.73869	419.2404619
2HESR_num2	49.0318	27159.73869	553.9208981	27159.73869	553.9208981
CHOLID	107.1144	20571.09954	192.047937	20571.09954	192.047937
4ABZGLUH	197.5637	9451.478628	47.84015802	9451.478628	47.84015802
PHDA	204.1006	32025.20046	156.9088991	32025.20046	156.9088991
TEO2M	45.0615	30564.75775	678.2898428	30564.75775	678.2898428
PACOAT	59.403	9300.979224	156.574234	9300.979224	156.574234
DHPTDNR	47.8104	15829.23579	331.0835257	15829.23579	331.0835257
DHPTDNRN	47.8104	25764.69374	538.8930806	25764.69374	538.8930806
PCNO	69.4475	20632.68563	297.0976008	20632.68563	297.0976008
HPACOAT	59.403	20199.22046	340.0370429	20199.22046	340.0370429
DC6PDA	27.7737	24868.36004	895.3924051	24868.36004	895.3924051
5DGLCNR	27.5627	10326.6768	374.6612923	10326.6768	374.6612923
5DKGR_num1	27.07	10950.24371	404.515837	10950.24371	404.515837
5DKGR_num2	27.5627	10950.24371	397.2848707	10950.24371	397.2848707
PYK6	205.4286	23858.79456	116.1415429	23858.79456	116.1415429
PAI2I	11.2548	9678.496108	859.9438557	9678.496108	859.9438557
CURR	75.2195	9968.899921	132.5307922	9968.899921	132.5307922
LKDRA	173.4961	14748.35399	85.00683297	14748.35399	85.00683297
METNA	38.4954	9848.755829	255.8424079	9848.755829	255.8424079
XYHDL	528.47	174757.0033	330.6809337	174757.0033	330.6809337

	62				
E4PP_num1	29.7077	23567.45492	793.3113274	23567.45492	793.3113274
E4PP_num2	23.0075	23567.45492	1024.33793	23567.45492	1024.33793
E4PP_num3	30.4128	23567.45492	774.918946	23567.45492	774.918946
E4PP_num4	20.7798	23567.45492	1134.152154	23567.45492	1134.152154
E4PP_num5	24.3302	23567.45492	968.650275	23567.45492	968.650275
PUACGAMS	66.8476	21535.02252	322.1510199	21535.02252	322.1510199
RU5PP	23.0075	9743.191204	423.4789179	9743.191204	423.4789179
6D6SFK	31.7277	24036.90232	757.5998992	24036.90232	757.5998992
3SLAR	124.6326	32163.07972	258.0631369	32163.07972	258.0631369
ARHGDx	176.7031	18504.6326	104.721607	18504.6326	104.721607
CPL	31.8451	55622.71588	1746.664821	55622.71588	1746.664821
INOSTO	61.1644	30029.78275	490.9683207	30029.78275	490.9683207
HPYRP	21.4356	20546.33029	958.5143541	20546.33029	958.5143541
PSURIK	33.573	18771.63941	559.1290445	18771.63941	559.1290445
QUINDHyi	62.4556	9660.842064	154.6833601	9660.842064	154.6833601
PNSPA	26.4826	20269.59981	765.3931191	20269.59981	765.3931191
4HTHRK	67.2473	25833.20273	384.1522668	25833.20273	384.1522668
PETNT181pp	63.8044	70069.97275	1098.199697	70069.97275	1098.199697
ARMEPNS	101.1252	96018.1492	949.4977434	96018.1492	949.4977434
RPNTPH	42.0104	863642.4721	20557.82549	863642.4721	20557.82549
PRCPD	55.6956	35200.69678	632.0193477	35200.69678	632.0193477
UDPGPT	52.408	67455.14386	1287.10312	67455.14386	1287.10312

	5				
OMMBLHXy	42.953<br>5	21532.4983	501.2978756	21532.4983	501.2978756
OPHHXy	44.244<br>6	9301.138592	210.2208765	9301.138592	210.2208765
PROD3	287.63<br>03	61607.80649	214.1909475	61607.80649	214.1909475
F1PP_num1	30.412<br>8	30106.44206	989.9266774	30106.44206	989.9266774
F1PP_num2	20.779<br>8	30106.44206	1448.832138	30106.44206	1448.832138
F1PP_num3	23.007<br>5	30106.44206	1308.549041	30106.44206	1308.549041
F1PP_num4	29.721<br>1	30106.44206	1012.965269	30106.44206	1012.965269
ACP1p	98.877<br>2	17920.62895	181.2412664	17920.62895	181.2412664
3HPADHi	53.418<br>6	29369.09112	549.7914794	29369.09112	549.7914794
2HPTCOAT	63.973<br>7	50248.12219	785.44968	50248.12219	785.44968
MC6PH	202.05	1075510.261	5322.990653	1075510.261	5322.990653
CYSTA	87.146<br>7	14883.03104	170.7813496	14883.03104	170.7813496
FE3DCITR5	28.871<br>6	12098.3979	419.0414766	12098.3979	419.0414766
4HTHRA	145.97<br>88	168654.009	1155.332205	168654.009	1155.332205
GAPP	24.330<br>2	71411.82725	2935.110572	71411.82725	2935.110572
PAI2T	318.92<br>74	9523.524841	29.86110582	9523.524841	29.86110582
DHCURR	75.219<br>5	9968.97298	132.5317634	9968.97298	132.5317634
RHMND	353.81<br>17	28119.95353	79.47717255	28119.95353	79.47717255
3HBZCT	59.880<br>4	29600.99634	494.335314	29600.99634	494.335314
CXSAMS	55.553<br>3	25116.46412	452.11471	25116.46412	452.11471
ACOAD1f	89.224<br>4	434993.0386	4875.269977	434993.0386	4875.269977
AMPNTAT	16.569	23261.95502	1403.876632	23261.95502	1403.876632

	8				
SQGH	77.275	31386.49569	406.1662335	31386.49569	406.1662335
QUINDH	62.4556	9439.253176	151.1354174	9439.253176	151.1354174
LCARSyi	37.9784	9500.890252	250.1656271	9500.890252	250.1656271
GTHOr_reverse	97.545	10041.29653	102.9401459	10041.29653	102.9401459
MAN6PI_reverse	42.85	59947.56896	1399.009777	59947.56896	1399.009777
TMDPP_reverse	94.4142	12184.75745	129.0564073	12184.75745	129.0564073
HCO3E_reverse_num1	100.3871	17055.96902	169.9019996	17055.96902	169.9019996
HCO3E_reverse_num2	47.5285	17055.96902	358.8577174	17055.96902	358.8577174
IMPC_reverse	57.3292	36000	627.9522477	36000	627.9522477
ECOAH4_reverse_num1	77.0722	34389.94493	446.2042725	34389.94493	446.2042725
ECOAH4_reverse_num2	159.1878	34389.94493	216.0337974	34389.94493	216.0337974
NDPK5_reverse_num1	23.586	10223.94078	433.4749758	10223.94078	433.4749758
NDPK5_reverse_num2	61.8538	10223.94078	165.2920399	10223.94078	165.2920399
DHORTS_reverse	77.6548	24480	315.2412987	24480	315.2412987
ICDHyr_reverse	91.5134	30368.82282	331.851104	30368.82282	331.851104
ACALD_reverse_num1	33.4425	142688.5307	4266.682534	142688.5307	4266.682534
ACALD_reverse_num2	3845.0896	142688.5307	37.10928626	142688.5307	37.10928626
ALAR_reverse_num1	77.6892	104292.6285	1342.434064	104292.6285	1342.434064
ALAR_reverse_num2	78.3059	104292.6285	1331.861692	104292.6285	1331.861692
ALATA_L_reverse_num1	91.0347	48870.10901	536.8294618	48870.10901	536.8294618
ALATA_L_reverse_num2	92.4324	48870.10901	528.7118912	48870.10901	528.7118912
PPM_reverse	44.37	32423.2523	730.7471783	32423.2523	730.7471783
ASPTA_reverse_num1	87.1467	75600	867.5027282	75600	867.5027282

ASPTA_reverse_num2	87.0756	75600	868.2110718	75600	868.2110718
A5PISO_reverse_num1	140.7843	33180.19039	235.681041	33180.19039	235.681041
A5PISO_reverse_num2	136.1257	33180.19039	243.7467017	33180.19039	243.7467017
IPPMIb_reverse	72.3693	123242.3232	1702.964147	123242.3232	1702.964147
PSCVT_reverse	46.0958	43650.2925	946.9472815	43650.2925	946.9472815
ECOAH6_reverse_num1	77.0722	46056.25922	597.5729151	46056.25922	597.5729151
ECOAH6_reverse_num2	159.1878	46056.25922	289.3202822	46056.25922	289.3202822
ENO_reverse	91.3099	51877.32098	568.1456335	51877.32098	568.1456335
FBA_reverse_num1	78.2945	34326.33312	438.4258552	34326.33312	438.4258552
FBA_reverse_num2	381.0919	34326.33312	90.0736361	34326.33312	90.0736361
PGI_reverse	123.0595	323462.6992	2628.506529	323462.6992	2628.506529
PGK_reverse	41.1182	151200	3677.203769	151200	3677.203769
RPE_reverse	24.5542	108366.9572	4413.377637	108366.9572	4413.377637
TALA_reverse_num1	70.4385	17007.88862	241.4572801	17007.88862	241.4572801
TALA_reverse_num2	35.6588	17007.88862	476.9618894	17007.88862	476.9618894
TKT1_reverse_num1	144.4235	21768.90264	150.7296433	21768.90264	150.7296433
TKT1_reverse_num2	73.0427	21768.90264	298.0298187	21768.90264	298.0298187
ALCD2x_reverse_num1	3845.0896	86341.98017	22.45512827	86341.98017	22.45512827
ALCD2x_reverse_num2	141.5183	86341.98017	610.1117677	86341.98017	610.1117677
RMI_reverse	188.7978	18114.75932	95.94793647	18114.75932	95.94793647
NDPK6_reverse_num1	23.586	9881.818517	418.9696649	9881.818517	418.9696649
NDPK6_reverse_num2	61.8538	9881.818517	159.7608961	9881.818517	159.7608961

NDPK8_reverse_num1	23.586	14417.88494	611.2899576	14417.88494	611.2899576
NDPK8_reverse_num2	61.8538	14417.88494	233.0961871	14417.88494	233.0961871
PYNP2r_reverse	162.9545	10466.8948	64.23200833	10466.8948	64.23200833
DURIPP_reverse_num1	94.4142	9672.277903	102.4451608	9672.277903	102.4451608
DURIPP_reverse_num2	155.6995	9672.277903	62.12144485	9672.277903	62.12144485
TRPAS2_reverse	211.0938	18000	85.27015005	18000	85.27015005
ILETA_reverse	204.5619	21960	107.3513689	21960	107.3513689
VALTA_reverse	204.5619	31680	154.8675486	31680	154.8675486
ORPT_reverse	47.134	17956.83112	380.9740552	17956.83112	380.9740552
PTAr_reverse_num1	463.0325	420628.6411	908.4214199	420628.6411	908.4214199
PTAr_reverse_num2	72.1335	420628.6411	5831.252346	420628.6411	5831.252346
ACKr_reverse_num1	42.4337	178357.4108	4203.201955	178357.4108	4203.201955
ACKr_reverse_num2	43.2904	178357.4108	4120.02224	178357.4108	4120.02224
ACKr_reverse_num3	43.3841	178357.4108	4111.123909	178357.4108	4111.123909
AIRC3_reverse	142.2426	46800	329.0153583	46800	329.0153583
ADSL2r_reverse	206.1712	15978.08037	77.49908994	15978.08037	77.49908994
PUNP5_reverse_num1	179.0077	9894.50248	55.27417245	9894.50248	55.27417245
PUNP5_reverse_num2	155.6995	9894.50248	63.54871069	9894.50248	63.54871069
PUNP6_reverse_num1	155.6995	9464.634994	60.78783165	9464.634994	60.78783165
PUNP6_reverse_num2	179.0077	9464.634994	52.87278141	9464.634994	52.87278141
PUNP7_reverse	179.0077	32703.33227	182.6923214	32703.33227	182.6923214
PUNP1_reverse	155.6995	36	0.231214615	36	0.231214615

PUNP2_reverse	155.6995	9843.444108	63.22078175	9843.444108	63.22078175
PUNP3_reverse_num1	179.0077	9803.384518	54.76515545	9803.384518	54.76515545
PUNP3_reverse_num2	155.6995	9803.384518	62.96349389	9803.384518	62.96349389
LYSAM_reverse	38.7496	25606.02051	660.8073504	25606.02051	660.8073504
IPPMIa_reverse	72.3693	106566.4071	1472.536104	106566.4071	1472.536104
MDH_reverse	64.6746	11663.48336	180.3410204	11663.48336	180.3410204
FUM_reverse_num1	120.2106	2156.14877	17.93642799	2156.14877	17.93642799
FUM_reverse_num2	120.5972	2156.14877	17.87892895	2156.14877	17.87892895
FUM_reverse_num3	201.9555	2156.14877	10.67635578	2156.14877	10.67635578
OHPBAT_reverse	79.5666	20025.08515	251.6770247	20025.08515	251.6770247
AGPR_reverse	35.9521	86400	2403.197588	86400	2403.197588
G1SAT_reverse	90.7322	38145.11774	420.4143374	38145.11774	420.4143374
GUI1_reverse	53.9872	18227.31552	337.6229091	18227.31552	337.6229091
PGAMT_reverse	47.5436	14400	302.8798829	14400	302.8798829
GLUR_reverse	31.002	75600	2438.552351	75600	2438.552351
UAG2E_reverse	84.4892	64758.73007	766.4734672	64758.73007	766.4734672
HACD5_reverse_num1	159.1878	28543.86811	179.309395	28543.86811	179.309395
HACD5_reverse_num2	77.0722	28543.86811	370.3523205	28543.86811	370.3523205
GHMT2r_reverse	90.6332	36640.10614	404.2680401	36640.10614	404.2680401
ADK4_reverse	23.586	27584.11545	1169.51223	27584.11545	1169.51223
KARA1_reverse	216.2761	8280	38.28439666	8280	38.28439666
KARA2_reverse	216.2761	11785.94812	54.49491701	11785.94812	54.49491701
ICHORS_copy2_r	97.529	11143.4815	114.2580164	11143.4815	114.2580164

everse	1				
DHBD_reverse	104.9986	15751.76703	150.0188292	15751.76703	150.0188292
RBP4E_reverse_num1	25.2777	22153.75973	876.4151693	22153.75973	876.4151693
RBP4E_reverse_num2	102.0756	22153.75973	217.0328631	22153.75973	217.0328631
RBP4E_reverse_num3	25.5609	22153.75973	866.7049957	22153.75973	866.7049957
HACD6_reverse_num1	159.1878	28885.7992	181.457368	28885.7992	181.457368
HACD6_reverse_num2	77.0722	28885.7992	374.788824	28885.7992	374.788824
ECOAH1_reverse_num1	77.0722	55686.49906	722.5238031	55686.49906	722.5238031
ECOAH1_reverse_num2	159.1878	55686.49906	349.8163745	55686.49906	349.8163745
ECOAH2_reverse_num1	77.0722	45311.23192	587.9062998	45311.23192	587.9062998
ECOAH2_reverse_num2	159.1878	45311.23192	284.6401038	45311.23192	284.6401038
ECOAH3_reverse_num1	77.0722	38981.49761	505.7789658	38981.49761	505.7789658
ECOAH3_reverse_num2	159.1878	38981.49761	244.877419	38981.49761	244.877419
UGLT_reverse	79.2914	10608.99987	133.7976107	10608.99987	133.7976107
XYLI2_reverse	198.968	58957.93451	296.3186769	58957.93451	296.3186769
PMANM_reverse	50.4626	15462.38488	306.4127666	15462.38488	306.4127666
UDPG4E_reverse	74.5302	59585.79647	799.4852619	59585.79647	799.4852619
XYLI1_reverse	198.968	12143.93448	61.03461097	12143.93448	61.03461097
LCARS_reverse	81.0267	20440.77639	252.2721078	20440.77639	252.2721078
ALCD19_reverse_num1	141.5183	147353.7592	1041.234662	147353.7592	1041.234662
ALCD19_reverse_num2	3845.0896	147353.7592	38.32258142	147353.7592	38.32258142
RMPA_reverse	120.582	10127.96802	83.99237053	10127.96802	83.99237053

SBTPD_reverse	111.4315	43431.09995	389.756038	43431.09995	389.756038
GLTPD_reverse	74.7803	26014.78708	347.8828927	26014.78708	347.8828927
TGBPA_reverse_num1	172.3671	11962.53261	69.40148445	11962.53261	69.40148445
TGBPA_reverse_num2	77.9206	11962.53261	153.5220803	11962.53261	153.5220803
MANAO_reverse	53.5802	66372.21763	1238.745239	66372.21763	1238.745239
TAGURr_reverse	54.8083	48756.38965	889.5804039	48756.38965	889.5804039
HACD4_reverse_num1	159.1878	31565.19803	198.2890525	31565.19803	198.2890525
HACD4_reverse_num2	77.0722	31565.19803	409.5536138	31565.19803	409.5536138
HACD7_reverse_num1	77.0722	23943.32058	310.6609203	23943.32058	310.6609203
HACD7_reverse_num2	159.1878	23943.32058	150.4092687	23943.32058	150.4092687
ADK1_reverse	23.586	16170.04165	685.5779551	16170.04165	685.5779551
SERASr_reverse	141.991	133230.4814	938.3022967	133230.4814	938.3022967
MLTP2_reverse	181.0448	20152.04116	111.3096933	20152.04116	111.3096933
CYTK1_reverse	24.7463	22437.44545	906.6989996	22437.44545	906.6989996
CYTK2_reverse	24.7463	10897.88195	440.3842979	10897.88195	440.3842979
GK1_reverse	47.1854	27785.55742	588.8592111	27785.55742	588.8592111
HACD1_reverse_num1	159.1878	41623.13358	261.4718815	41623.13358	261.4718815
HACD1_reverse_num2	77.0722	41623.13358	540.0537883	41623.13358	540.0537883
HACD2_reverse_num1	159.1878	39702.90928	249.4092467	39702.90928	249.4092467
HACD2_reverse_num2	77.0722	39702.90928	515.1391718	39702.90928	515.1391718
NDPK7_reverse_num1	23.586	10352.40646	438.9216678	10352.40646	438.9216678
NDPK7_reverse_num2	61.8538	10352.40646	167.3689645	10352.40646	167.3689645

GLYAT_reverse	86.2341	50081.79355	580.7655388	50081.79355	580.7655388
CRNBTCT_reverse	90.2535	128941.3418	1428.657523	128941.3418	1428.657523
CRNCBCT_reverse	90.2535	23925.28542	265.0898349	23925.28542	265.0898349
CRNCDH_reverse	28.1902	14573.28508	516.9628127	14573.28508	516.9628127
NDPK2_reverse_num1	23.586	13143.65765	557.2652273	13143.65765	557.2652273
NDPK2_reverse_num2	61.8538	13143.65765	212.4955565	13143.65765	212.4955565
NDPK3_reverse_num1	61.8538	23476.2708	379.5445194	23476.2708	379.5445194
NDPK3_reverse_num2	23.586	23476.2708	995.3476976	23476.2708	995.3476976
NDPK4_reverse_num1	23.586	25751.50973	1091.813352	25751.50973	1091.813352
NDPK4_reverse_num2	61.8538	25751.50973	416.328661	25751.50973	416.328661
DGK1_reverse	47.1854	10053.54912	213.0648277	10053.54912	213.0648277
DTMPK_reverse	23.7831	22465.87078	944.6149062	22465.87078	944.6149062
NDPK1_reverse_num1	23.586	9805.867045	415.7494719	9805.867045	415.7494719
NDPK1_reverse_num2	61.8538	9805.867045	158.5329769	9805.867045	158.5329769
PGMT_reverse_num1	58.361	29407.45925	503.8888855	29407.45925	503.8888855
PGMT_reverse_num2	20.7798	29407.45925	1415.194528	29407.45925	1415.194528
PGMT_reverse_num3	23.5649	29407.45925	1247.93482	29407.45925	1247.93482
UMPK_reverse_num1	24.7463	27813.81124	1123.958379	27813.81124	1123.958379
UMPK_reverse_num2	155.8216	27813.81124	178.49779	27813.81124	178.49779
DDPGALA_reverse	21.3906	53869.17244	2518.357243	53869.17244	2518.357243
F6PA_reverse_num1	229.9682	19796.27081	86.0826445	19796.27081	86.0826445
F6PA_reverse_num2	23.5552	19796.27081	840.4204085	19796.27081	840.4204085

IDOND_reverse	37.1466	19846.42749	534.2730556	19846.42749	534.2730556
MCOATA_reverse	41.0567	9518.869696	231.8469262	9518.869696	231.8469262
DAPE_reverse	60.4171	109969.7887	1820.176552	109969.7887	1820.176552
SDPTA_reverse_num1	91.5493	27000	294.9230633	27000	294.9230633
SDPTA_reverse_num2	44.7291	27000	603.6338759	27000	603.6338759
SDPTA_reverse_num3	87.5339	27000	308.4519255	27000	308.4519255
SDPTA_reverse_num4	87.3307	27000	309.1696276	27000	309.1696276
SDPTA_reverse_num5	79.5666	27000	339.3383656	27000	339.3383656
ARAI_reverse	336.4439	37923.43532	112.7184512	37923.43532	112.7184512
M1PD_reverse	41.139	24491.61456	595.3381113	24491.61456	595.3381113
G3PD2_reverse	72.7232	122400	1683.094253	122400	1683.094253
FCI_reverse	389.86	60125.13198	154.222367	60125.13198	154.222367
FCLPA_reverse_num1	23.2225	19739.33073	850.0088589	19739.33073	850.0088589
FCLPA_reverse_num2	95.1013	19739.33073	207.561103	19739.33073	207.561103
UAGAAT_reverse	92.8795	9875.081682	106.3214346	9875.081682	106.3214346
PRPPS_reverse	34.2183	9727.446006	284.2761331	9727.446006	284.2761331
PRMICI_reverse	26.0326	38487.18989	1478.422819	38487.18989	1478.422819
SERAT_reverse	175.8998	56837.93429	323.1267704	56837.93429	323.1267704
ASPK_reverse_num1	177.7754	131867.8614	741.7666415	131867.8614	741.7666415
ASPK_reverse_num2	356.481	131867.8614	369.9155394	131867.8614	369.9155394
ASPK_reverse_num3	97.0635	131867.8614	1358.573113	131867.8614	1358.573113
ASAD_reverse	80.0358	22680	283.3731905	22680	283.3731905
CBMKr_reverse	31.644	10062.30195	317.9835087	10062.30195	317.9835087

	1				
AMANAPEr_reverse	24.0737	586980.0616	24382.62758	586980.0616	24382.62758
NNATr_reverse	24.528	41349.30422	1685.800074	41349.30422	1685.800074
GAPD_reverse	142.13	56839.88531	399.9147633	56839.88531	399.9147633
ARGSL_reverse	50.3182	10424.18161	207.1652327	10424.18161	207.1652327
ACOATA_reverse	75.6698	9801.989935	129.5363531	9801.989935	129.5363531
MLTP3_reverse	181.0448	10299.24688	56.88783594	10299.24688	56.88783594
OCBT_reverse_num1	110.7213	19557.83972	176.6402645	19557.83972	176.6402645
OCBT_reverse_num2	110.4814	19557.83972	177.0238223	19557.83972	177.0238223
GARFT_reverse	23.2383	40052.3476	1723.548951	40052.3476	1723.548951
PHETA1_reverse_num1	87.1467	31007.86386	355.8122553	31007.86386	355.8122553
PHETA1_reverse_num2	87.0756	31007.86386	356.1027873	31007.86386	356.1027873
PHETA1_reverse_num3	204.5619	31007.86386	151.5818139	31007.86386	151.5818139
MCITL2_reverse	128.5386	20380.45042	158.5550988	20380.45042	158.5550988
ACOTA_reverse_num1	91.5493	32625.37856	356.3695032	32625.37856	356.3695032
ACOTA_reverse_num2	87.3307	32625.37856	373.5843015	32625.37856	373.5843015
ACOTA_reverse_num3	87.5339	32625.37856	372.717068	32625.37856	372.717068
ACOTA_reverse_num4	44.7291	32625.37856	729.3993968	32625.37856	729.3993968
PRAGSr_reverse	45.9404	17654.10147	384.2827112	17654.10147	384.2827112
PPAKr_reverse	43.3841	9938.898806	229.0908145	9938.898806	229.0908145
PPM2_reverse	44.37	26740.7499	602.6763555	26740.7499	602.6763555
G6PDH2r_reverse	55.7044	27844.11588	499.8548746	27844.11588	499.8548746
FBA3_reverse	78.2945	10772.90842	137.5947023	10772.90842	137.5947023
ADK3_reverse	23.586	12279.19346	520.6136462	12279.19346	520.6136462

HSDy_reverse_num1	177.7754	638760.4157	3593.075396	638760.4157	3593.075396
HSDy_reverse_num2	356.481	638760.4157	1791.84982	638760.4157	1791.84982
ACACT7r_reverse_num1	46.5305	26653.29045	572.8133256	26653.29045	572.8133256
ACACT7r_reverse_num2	81.7524	26653.29045	326.0245626	26653.29045	326.0245626
ACACT2r_reverse_num1	46.5305	48623.593	1044.983247	48623.593	1044.983247
ACACT2r_reverse_num2	81.7524	48623.593	594.7665511	48623.593	594.7665511
ACACT3r_reverse_num1	46.5305	36960.01261	794.3179766	36960.01261	794.3179766
ACACT3r_reverse_num2	81.7524	36960.01261	452.0969734	36960.01261	452.0969734
ACACT4r_reverse_num1	46.5305	34554.87723	742.6285388	34554.87723	742.6285388
ACACT4r_reverse_num2	81.7524	34554.87723	422.6772208	34554.87723	422.6772208
AHGDx_reverse	176.7031	28540.16407	161.5147899	28540.16407	161.5147899
TKT2_reverse_num1	144.4235	149806.8491	1037.274745	149806.8491	1037.274745
TKT2_reverse_num2	73.0427	149806.8491	2050.948954	149806.8491	2050.948954
ATPS4rpp_reverse_num1	543.6444	394887.4078	726.3707816	394887.4078	726.3707816
ATPS4rpp_reverse_num2	530.0127	394887.4078	745.0527275	394887.4078	745.0527275
ECOAH8_reverse_num1	77.0722	24530.3731	318.2778369	24530.3731	318.2778369
ECOAH8_reverse_num2	159.1878	24530.3731	154.0970671	24530.3731	154.0970671
ECOAH5_reverse_num1	77.0722	31162.6381	404.3304602	31162.6381	404.3304602
ECOAH5_reverse_num2	159.1878	31162.6381	195.7602159	31162.6381	195.7602159
TRSARr_reverse_num1	123.2023	21843.31589	177.296332	21843.31589	177.296332
TRSARr_reverse_num2	30.4274	21843.31589	717.8830886	21843.31589	717.8830886
ACACT5r_reverse_num1	81.7524	35501.71811	434.2590323	35501.71811	434.2590323

ACACT5r_reverse_num2	46.5305	35501.71811	762.9773614	35501.71811	762.9773614
MTHFC_reverse	62.0875	53981.9901	869.450213	53981.9901	869.450213
MTHFD_reverse	62.0875	28796.28731	463.8016881	28796.28731	463.8016881
ACACT6r_reverse_num1	46.5305	29980.30132	644.3150476	29980.30132	644.3150476
ACACT6r_reverse_num2	81.7524	29980.30132	366.7207486	29980.30132	366.7207486
GGGABADr_reverse	53.4186	20083.16025	375.9581915	20083.16025	375.9581915
MALTATr_reverse	60.288	10202.84671	169.2351166	10202.84671	169.2351166
ALAGLUE_reverse	34.674	22236.80583	641.3106602	22236.80583	641.3106602
CBLAT_reverse	21.9989	27183.75746	1235.687124	27183.75746	1235.687124
ARABDI_reverse	389.86	45079.5164	115.6300118	45079.5164	115.6300118
FRULYSDG_reverse	462.8308	10651.82164	23.01450474	10651.82164	23.01450474
ALLPI_reverse	32.1466	10258.17606	319.106097	10258.17606	319.106097
ALLULPE_reverse	26.1092	110777.1111	4242.838199	110777.1111	4242.838199
DHPTPE_reverse	112.6563	17788.09141	157.8969965	17788.09141	157.8969965
FALDH2_reverse	78.718	22410.33031	284.691307	22410.33031	284.691307
CBIAT_reverse	21.9989	21148.31387	961.3350609	21148.31387	961.3350609
GLUDy_reverse_num1	291.4882	666000	2284.826624	666000	2284.826624
GLUDy_reverse_num2	861.249	666000	773.2955278	666000	773.2955278
HACD3_reverse_num1	159.1878	36814.26424	231.2631008	36814.26424	231.2631008
HACD3_reverse_num2	77.0722	36814.26424	477.6594445	36814.26424	477.6594445
E4PD_reverse_num1	149.1976	19375.48784	129.864608	19375.48784	129.864608
E4PD_reverse_num2	142.13	19375.48784	136.3222954	19375.48784	136.3222954
DADK_reverse	23.586	13343.77709	565.7498978	13343.77709	565.7498978

CRNCAR_reverse	28.1902	20738.59238	735.6667346	20738.59238	735.6667346
DHFR_reverse_num1	26.3483	20559.74064	780.3061543	20559.74064	780.3061543
DHFR_reverse_num2	17.9994	20559.74064	1142.245888	20559.74064	1142.245888
TYRTA_reverse_num1	87.1467	24103.01587	276.5797886	24103.01587	276.5797886
TYRTA_reverse_num2	87.0756	24103.01587	276.8056248	24103.01587	276.8056248
TYRTA_reverse_num3	204.5619	24103.01587	117.8274931	24103.01587	117.8274931
PUNP4_reverse_num1	179.0077	9382.349419	52.41310524	9382.349419	52.41310524
PUNP4_reverse_num2	155.6995	9382.349419	60.259342	9382.349419	60.259342
AMAOTr_reverse	94.6712	32523.36129	343.5401821	32523.36129	343.5401821
GLCATr_reverse	60.288	10000.12983	165.8726418	10000.12983	165.8726418
ALAALAr_reverse_num1	39.3158	20079.23664	510.7167256	20079.23664	510.7167256
ALAALAr_reverse_num2	65.6794	20079.23664	305.7158963	20079.23664	305.7158963
ACACT1r_reverse_num1	46.5305	49056.7815	1054.293023	49056.7815	1054.293023
ACACT1r_reverse_num2	161.4099	49056.7815	303.9267201	49056.7815	303.9267201
ACACT1r_reverse_num3	81.7524	49056.7815	600.0653375	49056.7815	600.0653375
SUCOAS_reverse	142.3403	36798.90163	258.5276386	36798.90163	258.5276386
GALKr_reverse	41.4421	27093.8564	653.7761455	27093.8564	653.7761455
YUMPS_reverse	98.7297	117059.4818	1185.656208	117059.4818	1185.656208
ADSL1r_reverse	206.1712	16835.37942	81.65728006	16835.37942	81.65728006
AICART_reverse	57.3292	30129.89223	525.5592652	30129.89223	525.5592652
PGM_reverse_num1	57.1128	242966.3202	4254.148285	242966.3202	4254.148285
PGM_reverse_num2	56.1939	242966.3202	4323.713431	242966.3202	4323.713431

TPI_reverse	53.9436	132458.5014	2455.499844	132458.5014	2455.499844
RPI_reverse_num1	45.7208	85593.19064	1872.084273	85593.19064	1872.084273
RPI_reverse_num2	32.1466	85593.19064	2662.589221	85593.19064	2662.589221
PERD_reverse	82.7353	29134.95894	352.1466526	29134.95894	352.1466526
ATHRDHr_reverse	108.9957	28339.93201	260.0096335	28339.93201	260.0096335
LSERDHr_reverse	108.9957	11184.7652	102.616573	11184.7652	102.616573
DSERDHr_reverse	108.9957	17622.70624	161.6825823	17622.70624	161.6825823
UDPKAAT_reverse	41.6498	44134.30026	1059.652153	44134.30026	1059.652153
MLTP1_reverse	181.0448	78285.20605	432.4079236	78285.20605	432.4079236
IPDDI_reverse	20.5083	55097.27431	2686.584179	55097.27431	2686.584179
DURADx_reverse	178.7966	14611.84152	81.72326273	14611.84152	81.72326273
ECOAH7_reverse_num1	77.0722	25171.19057	326.5923455	25171.19057	326.5923455
ECOAH7_reverse_num2	159.1878	25171.19057	158.122611	25171.19057	158.122611
LDH_D_reverse	146.1392	11290.79911	77.26057836	11290.79911	77.26057836
T2DECAI_reverse	37.9381	17543.29767	462.4189843	17543.29767	462.4189843
CLPNS180pp_reverse_num1	54.8223	9824.361941	179.2037536	9824.361941	179.2037536
CLPNS180pp_reverse_num2	47.6335	9824.361941	206.2490042	9824.361941	206.2490042
CLPNS181pp_reverse_num1	54.8223	10525.55087	191.9939672	10525.55087	191.9939672
CLPNS181pp_reverse_num2	47.6335	10525.55087	220.969504	10525.55087	220.969504
CLPNS120pp_reverse_num1	47.6335	17185.48773	360.7857439	17185.48773	360.7857439
CLPNS120pp_reverse_num2	54.8223	17185.48773	313.4762265	17185.48773	313.4762265
CLPNS160pp_reverse_num1	47.6335	19150.79185	402.0446083	19150.79185	402.0446083

CLPNS160pp_reverse_num2	54.8223	19150.79185	349.3248522	19150.79185	349.3248522
CLPNS161pp_reverse_num1	54.8223	13654.34394	249.0655069	13654.34394	249.0655069
CLPNS161pp_reverse_num2	47.6335	13654.34394	286.6542232	13654.34394	286.6542232
HPYRI_reverse	58.7549	10252.32424	174.4930931	10252.32424	174.4930931
GUI2_reverse	53.9872	1106757.021	20500.35972	1106757.021	20500.35972
3OAR120_reverse	102.2412	168004.9209	1643.221332	168004.9209	1643.221332
ACONTb_reverse_num1	186.9962	58615.67725	313.4591893	58615.67725	313.4591893
ACONTb_reverse_num2	195.3544	58615.67725	300.0478989	58615.67725	300.0478989
MICITDr_reverse	186.9962	42684.56006	228.264318	42684.56006	228.264318
ACONTa_reverse_num1	186.9962	34269.90152	183.2652296	34269.90152	183.2652296
ACONTa_reverse_num2	195.3544	34269.90152	175.4242624	34269.90152	175.4242624
3OAR140_reverse	102.2412	10447.04323	102.1803659	10447.04323	102.1803659
3OAR160_reverse	102.2412	15760.16442	154.1469038	15760.16442	154.1469038
CLPNS140pp_reverse_num1	54.8223	25952.24174	473.3884157	25952.24174	473.3884157
CLPNS140pp_reverse_num2	47.6335	25952.24174	544.8317201	25952.24174	544.8317201
CLPNS141pp_reverse_num1	54.8223	18706.27168	341.21647	18706.27168	341.21647
CLPNS141pp_reverse_num2	47.6335	18706.27168	392.7125171	18706.27168	392.7125171
3OAR60_reverse	102.2412	16171.49843	158.1700765	16171.49843	158.1700765
3OAR80_reverse	102.2412	41062.49348	401.6237435	41062.49348	401.6237435
3OAR40_reverse	102.2412	15479.59285	151.4026914	15479.59285	151.4026914
3OAR100_reverse	102.2412	21350.28	208.8226664	21350.28	208.8226664
3OAR180_reverse	102.2412	11764.09956	115.0622211	11764.09956	115.0622211

ACOAD7f_reverse	89.2244	460800	5164.50657	460800	5164.50657
ACOAD8f_reverse	89.2244	323145.3704	3621.715253	323145.3704	3621.715253
ACOAD5f_reverse	89.2244	1281600	14363.7839	1281600	14363.7839
ACOAD3f_reverse	89.2244	1281600	14363.7839	1281600	14363.7839
ACOAD2f_reverse	89.2244	1281600	14363.7839	1281600	14363.7839
ACOAD4f_reverse	89.2244	1281600	14363.7839	1281600	14363.7839
ACOAD6f_reverse	89.2244	1000800	11216.66271	1000800	11216.66271
FRULYSE_reverse	249.3506	26511.74296	106.3231569	26511.74296	106.3231569
POR5_reverse_num1	148.5614	52980.56892	356.6240552	52980.56892	356.6240552
POR5_reverse_num2	148.5246	52980.56892	356.7124161	52980.56892	356.7124161
HACD8_reverse_num1	159.1878	23805.65727	149.5444831	23805.65727	149.5444831
HACD8_reverse_num2	77.0722	23805.65727	308.8747599	23805.65727	308.8747599
CTECOAI8_reverse	159.1878	51918.91394	326.1488251	51918.91394	326.1488251
CTECOAI6_reverse	159.1878	29128.12964	182.9796607	29128.12964	182.9796607
REPHACCOAI_reverse	28.4046	10703.34754	376.8174006	10703.34754	376.8174006
DHNPTE_reverse	108.9562	10065.04768	92.37700728	10065.04768	92.37700728
FORCT_reverse	91.6564	129064.0685	1408.129367	129064.0685	1408.129367
HADPCOADH3_reverse	51.7326	18258.39275	352.9378526	18258.39275	352.9378526
ACACT8r_reverse_num1	46.5305	30393.77583	653.201144	30393.77583	653.201144
ACACT8r_reverse_num2	81.7524	30393.77583	371.7783922	30393.77583	371.7783922
CTECOAI7_reverse	159.1878	198000	1243.81391	198000	1243.81391
DHACOAH_reverse	27.2374	12132.60569	445.439201	12132.60569	445.439201

ALDD19xr_reverse	214.7965	9300.75871	43.3003271	9300.75871	43.3003271
VPAMTr_reverse	46.7112	10207.799	218.5300098	10207.799	218.5300098
13PPDH2_reverse	84.194	10451.44479	124.1352684	10451.44479	124.1352684
GHBDHx_reverse	124.6326	135145.7337	1084.353	135145.7337	1084.353
APPLDHr_reverse	309.6976	20318.82558	65.60859878	20318.82558	65.60859878
DHNPA2r_reverse	108.9562	11082.16412	101.7121019	11082.16412	101.7121019
NADHXE_reverse	54.6503	13156.18454	240.7339858	13156.18454	240.7339858
NADPHXE_reverse	54.6503	922865.2178	16886.73654	922865.2178	16886.73654
DXYLTD_reverse_num1	69.3992	71697.11216	1033.111508	71697.11216	1033.111508
DXYLTD_reverse_num2	70.0168	71697.11216	1023.9987	71697.11216	1023.9987
2DDARAA_reverse_num1	32.7217	9688.408474	296.0851201	9688.408474	296.0851201
2DDARAA_reverse_num2	130.1216	9688.408474	74.45657349	9688.408474	74.45657349
ACOXT_reverse	83.3415	10077.21241	120.9146993	10077.21241	120.9146993
4ABZGLUH_reverse	197.5637	31226.50825	158.0579238	31226.50825	158.0579238
PCNO_reverse	69.4475	18766.78821	270.2298601	18766.78821	270.2298601
5DKGR_reverse_num1	27.07	23521.17739	868.9020092	23521.17739	868.9020092
5DKGR_reverse_num2	27.5627	23521.17739	853.3698582	23521.17739	853.3698582
LKDRA_reverse	173.4961	9819.411113	56.59730168	9819.411113	56.59730168
METNA_reverse	38.4954	27085.97543	703.6158978	27085.97543	703.6158978
PUACGAMS_reverse	66.8476	82566.7816	1235.149528	82566.7816	1235.149528
ARHGDx_reverse	176.7031	9808.83436	55.51025624	9808.83436	55.51025624
INOSTO_reverse	61.1644	28380.97591	464.0113515	28380.97591	464.0113515

UDPGPT_reverse	52.4085	46403.30621	885.4156522	46403.30621	885.4156522
4HTHRA_reverse	145.9788	16492.0015	112.975319	16492.0015	112.975319
RHMND_reverse	353.8117	16526.48756	46.70983904	16526.48756	46.70983904

Table S4 Enzyme cost of energy metabolism in *E. coli*

	ATP produced per flux of glucose (mmol/gDW/h)	Enzyme required per flux of glucose (mg enzyme / gDW)	Enzyme cost (g enzyme for 1 mol ATP / h)
High-yield pathway	23.5	55.99	2.38
Low-yield pathway	11	6.83	0.62

Table S5 The maximum growth rates

**Carbon sources name**	**measured (h^{-1})**	**predicted by eciML1515 (h^{-1})**	**predicted by iML1515(h^{-1})**	**predicted by GECKO(h^{-1})**	**predicted by sMOMENT (h^{-1})**
N-Acetyl-D-glucosamine	0.61 ±0.03	0.618	1.122	0.215	0.215
Acetate	0.29 ±0.02	0.209	0.209	0.133	0.133
2-Oxoglutarate	0.24 ±0.04	0.473	0.543	0.189	0.189
L-Alanine	0.24 ±0.03	0.347	0.378	0.161	0.161
Fructose	0.54 ±0.04	0.677	0.870	0.202	0.202
Fumarate	0.47 ±0.03	0.395	0.409	0.199	0.199
Glucose 6-phosphate	0.78 ±0.04	0.693	0.898	0.275	0.275
Galactose	0.24 ±0.02	0.575	0.860	0.209	0.209
Glucosamine	0.4 ±0.03	0.653	0.870	0.197	0.197
Gluconate	0.68 ±0.03	0.626	0.792	0.206	0.206

Glucose	0.66 ±0.05	0.680	0.870	0.223	0.223
Glycerol	0.47 ±0.03	0.428	0.492	0.227	0.227
Guanosine	0.37 ±0.03	0.475	1.030	0.267	0.267
L-Lactate	0.41 ±0.03	0.347	0.347	0.168	0.168
Maltose	0.52 ±0.02	0.602	1.765	0.148	0.148
L-Malate	0.55 ±0.03	0.409	0.409	0.192	0.192
Mannose	0.35 ±0.03	0.652	0.870	0.20	0.20
Mannitol	0.61 ±0.03	0.657	0.935	0.22	0.22
Pyruvate	0.41 ±0.03	0.312	0.312	0.152	0.152
Ribose	0.41 ±0.01	0.538	0.683	0.171	0.171
Sorbitol	0.48 ±0.03	0.550	0.935	0.218	0.218
Succinate	0.5 ±0.02	0.426	0.447	0.195	0.195
Trehalose	0.48 ±0.04	0.672	1.765	0.245	0.245
Xylose	0.51 ±0.03	0.379	0.711	0.177	0.177
